# Catastrophic health expenditure, social protection coverage, and financial coping strategies in adults with symptoms of chronic respiratory diseases in Kenya: a cross-sectional study

**DOI:** 10.1016/S2214-109X(25)00061-0

**Published:** 2025-06-25

**Authors:** Stephen Mulupi, Caroline Waithera, Ewan M Tomeny, Uzochukwu Egere, Helen Meme, Beatrice Kirubi, Jeremiah Chakaya, Edwine Barasa, Miriam Taegtmeyer, Tom Wingfield, NIHR International Multidisciplinary Programme to Address Lung Health and TB in Africa Consortium

**Affiliations:** a Institute for Resilient Health Systems, Department of International Public Health, Liverpool School of Tropical Medicine, Liverpool, UK; b Centre for Respiratory Diseases Research, Kenya Medical Research Institute, Nairobi, Kenya; c Centre for Tuberculosis Research, Department of Clinical Sciences, Liverpool School of Tropical Medicine, Liverpool, UK; d Department of Global Public Health, Karolinska Institutet, Stockholm, Sweden; e Tropical and Infectious Diseases Unit, Liverpool University Hospital NHS Foundation Trust, Liverpool, UK; f Respiratory Society of Kenya, Nairobi, Kenya; g Health Economics Research Unit, KEMRI–Wellcome Trust Research Programme, Nairobi, Kenya

## Abstract

**Background:**

Despite the socioeconomic consequences of an increasing burden of chronic respiratory diseases, there is little evidence on the incidence and determinants of catastrophic health expenditure (CHE) in people with chronic respiratory disease symptoms in Kenya. We aimed to generate this evidence by collecting data on medical and non-medical costs, lost income, social protection coverage, and financial coping strategies for such people in Kenya.

**Methods:**

We conducted a cross-sectional survey of consecutively recruited adults seeking care for chronic respiratory disease symptoms at five public health-care facilities in Meru County, Kenya, between Sept 5, 2019, and Oct 1, 2020. Patient costs, National Health Insurance Fund (NHIF) coverage, financial coping strategies, and sociodemographic and clinical data were collected from surveys and medical records. The main study outcomes were the incidence of, and social and health factors associated with, CHE in this cohort. CHE incidence was calculated through the WHO threshold of direct out-of-pocket costs being greater than 10% of a household's monthly total expenditure. Multivariable logistic regression analyses generated adjusted odds ratios (aORs) with 95% CIs of health and social factors associated with CHE, including age, sex, education level, tobacco use, income, being accompanied, poverty level (with the first quintile being the richest to the fifth quintile being the poorest), NHIF usage, coping strategies, final diagnosis, and health system level at which they were seeking care.

**Findings:**

Of 319 eligible people invited, 296 (93%) consented to participate and completed surveys. Mean total cost was 1062 Kenyan shillings (KES; 95% CI 896–1228; US$9·1), of which 40·0% was due to direct non-medical costs (KES 425, 95% CI 361–489; $3·7), 36·7% to direct out-of-pocket medical costs (KES 390, 324–456; $3·1), and 23·3% to lost income (KES 247, 153–341, $2·1). 212 (72%) of 296 participants did not have NHIF, 282 (95%) of 296 used coping strategies during care-seeking, and 59 (20%) of 296 were accompanied by a carer during health-care seeking. 76 (26%) of 296 participants had CHE. CHE was associated with being aged 30–44 years old (aOR 2·6, 95% CI 1·3–5·3, p=0·010), being female (1·8, 1·3–2·7, p=0·0021), having higher than secondary school education (1·6, 1·1–2·3, p=0·0083), being accompanied during health-care-seeking visits (3·2, 1·7–5·9, p<0·0001), belonging to the second poverty quintile (2·0, 1·9–2·1, p<0·0001), and seeking care from subcounty hospitals (9·7, 9·6–9·8, p<0·0001) and county hospitals (25·1, 15·7–40·2, p<0·0001).

**Interpretation:**

These findings suggest a sizeable burden of CHE in people seeking care for chronic respiratory disease symptoms in Meru County in Kenya, driven by socioeconomic and sex inequalities and impaired access to health care and social protection.

**Funding:**

UK-Aid, UK National Institute for Health and Care Research, and UK Research and Innovation.

## Introduction

Chronic respiratory diseases, such as asthma and chronic obstructive pulmonary disease (COPD), are among the four main non-communicable diseases (NCDs) that account for 80% of premature deaths related to NCDs globally.^1^ People with chronic respiratory disease can have chronic impairment of health and wellbeing and negative socioeconomic consequences. Sustainable Development Goal (SDG) 3.4 is to reduce the burden of premature deaths due to NCDs by 30% by 2030 “through prevention and treatment”.^2^ The targets for SDG 3.4 include strengthening the primary health-care system, increasing the coverage of social protection, and working towards reaching universal health coverage (UHC) to reduce patients’ direct out-of-pocket (OOP) costs and catastrophic health expenditure (CHE).^2^, ^3^ Such strategies are urgently needed to tackle the clinical and socioeconomic consequences of the rising burden of NCDs (including chronic respiratory diseases) in Kenya, a lower-middle-income country in sub-Saharan Africa, where an estimated one-in-ten people have asthma^4^ but where population-based surveys across regions and age groups have not been done and the true burden of chronic respiratory diseases remains unknown.

**Research in context**

**Evidence before this study**

We did a PubMed and grey literature search with the terms chronic respiratory diseases, financial protection, social protection, social health insurance, and universal health coverage to identify primary research, reports, and policy documents published in English between 2000 and 2024. The search found scarce evidence on catastrophic health expenditure (CHE), financial coping strategies, and health and social protection coverage and usage in people seeking care for chronic respiratory disease symptoms, especially in low-income and middle-income countries. The single primary research study identified from Kenya involved estimates of costs of care for people with asthma based on expert opinion and market rates for drug prices rather than actual expenditure of those affected.

**Added value of this study**

Among people seeking care for chronic respiratory disease symptoms in five public health-care facilities in Kenya, direct out-of-pocket (OOP) non-medical costs (eg, food, travel, and accommodation of participants and their accompanying carers) contributed 40% of total costs. Direct OOP medical costs (eg, consultations, medicines, and tests) contributed 37% of total costs and lost income contributed 23%. Nearly three-quarters of participants were not covered by the National Health Insurance Fund (NHIF), with commonly cited reasons including being unaware of NHIF or perceiving NHIF to be unaffordable. Even in the minority of participants with NHIF, most did not use it. Reasons reported for not using NHIF were not being asked about NHIF, not being up-to-date with NHIF premium payments, or NHIF not being accepted by the health-care facility attended. Nearly all (95%) participants had resorted to coping strategies due to chronic respiratory disease symptoms and care seeking, with the most common strategies being use of savings or borrowing money. Independent drivers of CHE included being female, poverty, being accompanied by a carer during health-care seeking, and seeking care at subcounty or county hospitals. In addition to generating novel data on the individual-level and household-level socioeconomic effect of care-seeking for chronic respiratory disease symptoms and the reasons behind lack of membership or usage of the NHIF, to our knowledge, this is the first study to estimate the incidence and drivers of WHO-defined CHE in people with chronic respiratory disease symptoms in Kenya.

**Implications of all the available evidence**

These novel findings suggest a sizeable burden of CHE in people seeking care for chronic respiratory disease symptoms in Meru County, Kenya, driven by socioeconomic and gender inequalities, and impaired health-care and social protection access. Given the dearth of similar evidence, it is unclear whether these results are generalisable to other counties in Kenya or more broadly. However, the findings provide renewed emphasis on the importance of working towards the Sustainable Development Goal targets of greater recognition, research, and investment into tackling non-communicable diseases, mitigating CHE, and (especially in the context of the ongoing attempts to roll out the new Social Health Insurance Fund, introduced in 2023 to replace NHIF) achieving universal health coverage in Kenya and other low-income and middle-income countries.

The Kenyan health-care system was devolved to 47 semiautonomous subnational (county) governments in 2013, following constitutional change.^5^ The system remains heavily reliant on OOP payments, which in 2020 accounted for 24% of total health expenditure and expose households to CHE.^6^, ^7^ WHO defines CHE as OOP health-care payments greater than 10% of a household's monthly total expenditure.^8^ CHE disproportionately affects the most economically vulnerable households, causing them to resort to financial coping strategies including using savings, borrowing money, taking loans, and selling household assets—a medical poverty trap that compounds their impoverishment.^9^ Between 2015 and 2017, CHE incidence increased from 12·7% to 13·2%, globally.^10^

The Kenyan Government's social health insurance scheme, the National Health Insurance Fund (NHIF), was established to cover costs of consultation, laboratory tests, radiology services and drugs, and maternity services, with the aim of achieving UHC and mitigating CHE in Kenya.^11^, ^12^ In 2023, the Kenyan Government introduced a new social health insurance programme, the Social Health Insurance Fund (SHIF), and terminated the NHIF. However, at the time of writing (April, 2025), roll-out of SHIF has proven challenging, with inconsistent coverage and use, partly due to legal disputes regarding the contributions structure, benefits package, and governance issues in both the private and public sectors. Other social protection measures applicable within public health-care facilities in Kenya include waivers of user fees for the poor and vulnerable on the recommendation of social medical or health-care workers.^7^, ^13^, ^14^

Kenya's efforts to improve UHC and mitigate CHE, through the former NHIF and the current SHIF, should be informed by up-to-date, context-relevant evidence on the costs to people of accessing care and on the use of social protection. This study aimed to generate new evidence on direct OOP medical and non-medical costs, lost income, financial coping strategies, NHIF coverage and usage, and incidence and drivers of CHE for people with chronic respiratory disease symptoms accessing public health-care facility services in Kenya.

## Methods

### Study design

We conducted a multicentre cross-sectional survey, consisting of interviews with adults (age ≥18 years) with symptoms of chronic respiratory disease attending stationary outpatient services in five public health-care facilities in Meru County, Kenya. Kenya has a devolved health system structured across six levels. L1 (the lowest health-care level) to L5 are financed and managed at subcounty and county levels, and L6 at the national level.^15^, ^16^ Meru County was selected because it was considered by the project team to be representative of the surrounding central region of Kenya based on health, health system, and sociodemographic indicators, including population density, health-care access, and poverty levels. Further details on the Kenyan health system and Meru County sociodemographic and health information are provided in the appendix 1 (pp 4–6).

This study was nested within the International Multidisciplinary Programme to Address Lung Health and TB in Africa. Meru County is one of ten counties that collectively account for 50% of Kenya's tuberculosis burden and is a priority county for active identification and treatment of people with symptoms of chronic respiratory disease.^17^ Our research team purposefully selected subcounties and their health-care facilities in consultation with the county health records officials and a tuberculosis coordinator. We based selection on up-to-date estimates of tuberculosis prevalence and considerations of accessibility and security for the study team. Tuberculosis prevalence was used as a selection criterion because it has been shown in a meta-analysis to be strongly associated with chronic respiratory disease prevalence due to overlapping social, clinical (including post-tuberculosis lung disease), and environmental factors.^18^ The proportion of health-care visits from people presenting to facilities with symptoms of chronic respiratory disease but negative tuberculosis tests was predicted to be substantial. We selected two health centres (Mitunguu and Laare), two subcounty hospitals (Kanyakine in Imenti South and Mutuati in Igembe North), and the County hospital (Meru Teaching and Referral Hospital). Further details can be found in the appendix 1 (pp 4–6).

This study was authorised by the Liverpool School of Tropical Medicine Research Ethics Committee, the Scientific and Ethical Review Unit of the Kenya Medical Research Institute (protocol 3848), and Meru County Department of Health.

### Study population

The study included adults aged 18–64 years with symptoms of chronic respiratory disease attending the outpatient departments in the five sampled public health facilities. This economically productive age group contributes the main proportion of chronic respiratory disease burden. In line with WHO's Engage TB toolkit for integrating community-based activities for tuberculosis and chronic respiratory diseases, symptoms were defined as any of the following signs or symptoms regardless of duration (as reported by the participant or documented by a health-care professional in the participant's medical notes): a cough, difficulty in breathing, noisy breathing, wheeze, chest tightness, or blood in sputum.^19^ Severely ill adults and those with impaired awareness were not invited to participate in the study due to inability to give informed consent; children (age <18 years) were excluded due to challenges in providing household income or expenditure data. All study participants provided written informed consent.

### Procedures

Adaptation and piloting of the costs survey, the data collection tools, and consenting processes are described in detail in appendix 1 (pp 7–35).

In each sampled facility, clinical officers (n=12) were trained for 2 days to identify eligible participants during routine history-taking and physical examination in medical consultation rooms. Eligible participants were linked to six trained field workers (two men and four women), with each consenting participant assigned a unique identification number. Data were collected in the medical consultation rooms by clinical officers and at the point of exit by the field workers from facilities during standard outpatient opening hours (0800–1800 h), Monday to Friday. In each facility, data were collected for a cumulative period of 4 weeks. Study data collection was initiated on Sept 5, 2019, and completed across all health-care facilities on Oct 1, 2020. The social, health, and economic data collected are detailed in the panel. Data were not collected on race or ethnicity. Meru County is a rural area inhabited predominantly by the Ameru ethnic group and these variables were not considered to influence healthcare access or CHE incidence in this context and therefore not included in the analysis plan. Data on sex were collected through self-reporting with the options of male or female. The question was understood by interviewers and respondents to refer to biological sex at birth.

Panel **Social, health, and economic data collected**

**Social data**

• Age
• Sex
• Education level
• Monthly participant and household income
• Participant self-identification as the primary income earner for their household (ie, the participant estimates that they earn more than half of the total household income)
• Asset ownership, including household furniture, electronic equipment, and livestock
• Access to amenities, including electricity and water
• Household expenditure on food, rent, travel, amenities, and leisure
• National Health Insurance Fund (NHIF) membership

**Health data**

• Accompaniment of participant by a carer or other person while seeking care
• Final diagnosis given by the health-care facility for condition causing presentation with symptoms of chronic respiratory disease
• Health system level at which participant sought care as a three-factor term (ie, health centre, subcounty hospital, or county hospital) in the primary analysis and a five-factor term (ie, each of the five individual health-care facilities at which participants were recruited) in the secondary analysis

**Economic data**

• Direct (out-of-pocket) medical costs: medical consultations, diagnostic tests (eg, laboratory and radiological imaging), medicines, and any other medical procedures (eg, bronchoscopy or pulmonary function testing)
• Direct (out-of-pocket) non-medical costs: return travel to the health centre (estimated by doubling the travel costs reported by the participant to have been incurred to reach the health centre), food, and accommodation were collected for both the participant and any carer or other person accompanying them
• Lost income: calculated through a human capital approach of reported days of work lost multiplied by estimated daily income.^20^ An output approach was not used to calculate lost income because participant estimates of income before health-care seeking were not provided
• Coping strategies associated with chronic respiratory disease symptoms, illness, and concomitant care-seeking: use of savings, borrowing from friends or relatives, money from other sources, additional work, selling property or assets, taking out loans, and defaulting on rent
• Reported NHIF membership and usage in care seeking, including predefined categories for not being a member of or using NHIF and a free-form other reason option

### Outcome measures

Coprimary outcome measures were the incidence of CHE in people seeking care for symptoms of chronic respiratory disease and the social and health factors associated with incurring CHE, including age, sex, education level, tobacco use, income, being accompanied by a carer or other person when seeking care, poverty level, NHIF usage, coping strategies, diagnosis, and health-system level at which they were seeking care. Secondary outcome measures were (1) the estimated direct OOP medical costs, direct OOP non-medical costs, and lost income of people seeking care for symptoms of chronic respiratory disease and their carer or accompanying person among the entire cohort and for participants who incurred CHE versus participants who did not incur CHE; (2) the estimated total costs (direct OOP medical costs plus direct OOP non-medical costs plus lost income) of people seeking care for symptoms of chronic respiratory disease and their carer or accompanying person among the entire cohort and for participants who incurred CHE versus participants who did not incur CHE; and (3) the coping strategies used, including by type and number of different coping strategies, and NHIF membership and usage (and reasons for non-membership and non-usage) among the entire cohort and for participants who incurred CHE versus participants who did not incur CHE. The perspective of costs analysis was the participant and their household.

### Statistical analysis

The 2017 Kenya TB Patient Cost Survey found 294 (27%) of 1071 people with tuberculosis incurred catastrophic costs but there was no available evidence to inform estimates of likely CHE incurrence among adults with symptoms of chronic respiratory diseases in Meru County.^21^ A sample size of 100 or more participants is considered suitable to estimate data on patient costs.^22^ To facilitate meaningful analysis of the socioeconomic and health factors associated with CHE incurrence, we aimed to recruit 300 participants.

Data were analysed through descriptive statistics, including simple counts, proportions, and mean or medians (depending on data distribution). In line with WHO and other patient cost surveys, data on continuous costs were primarily described with arithmetic means and SDs, regardless of their distribution. The median and IQRs of such cost data were also calculated for comparison. Participant and household income and expenditure data were highly skewed, with some participants reporting no household income or expenditure and some unable to report household income or expenditure or household asset data to inform the PCA-derived poverty score. Therefore, income and expenditure data first underwent log transformation to address non-normality; then multiple imputation with chained equations incorporating sociodemographic, clinical, and health facility variables as predictors replacing missing values; and finally had imputed full income and expenditure datasets subsequently back-transformed through exponential transformation. In addition, for the 21 participants without household asset data, multiple imputation with chained equations was also used to impute poverty scores.^23^, ^24^ Mean total direct OOP costs (ie, total direct medical plus total direct non-medical costs) and total costs (ie, total direct costs plus lost income) were calculated. To facilitate generalisability, CHE was calculated according to WHO's definition of total direct costs of illness and care-seeking of more than 10% of total household monthly expenditure.^8^, ^25^ This binary CHE threshold was then used as the dependent variable in the main regression model. We used principal component analysis of asset ownership to derive a cohort-specific poverty score, which was converted into poverty quintiles (the richest being the first quintile and the poorest being the fifth quintile) and included as an independent variable in multivariable regression analyses.^26^ For further details on methods for principal component analysis and poverty score generation, see appendix 1 (p 36).

For non-parametric continuous variables, including age, participant income, household income, and household expenditure, we generated 95% bootstrapped CIs and compared these between participants whose households had versus had not incurred CHE via the Mann–Whitney U Test. For the remaining binary categorical variables, we generated 95% Wilson CIs of proportions and compared these between participants whose households had versus had not incurred CHE via Pearson's χ^2^ test or, when values were less than 5 (ie, the final diagnosis variable), Fisher's exact test.

Univariable and multivariable logistic regression models generated unadjusted and adjusted odds ratios (aORs) with 95% CIs of the association of social and health variables, including level of care sought (eg, health centre, subcounty hospital, or county hospital), with CHE. Variables or subcategories associated with CHE in the univariable model at a level of p<0·15 were included in the multivariable model,^27^, ^28^ which was adjusted for age, sex, and education level. Coping strategies were included in the model as an ordinal variable: no coping strategies, a single coping strategy used, more than one coping strategy used.

Analyses were not adjusted for multiplicity. Interaction terms were used to evaluate effect modification through the STATA mfpigen command and complemented with the likelihood ratio test (appendix 1 pp 37–38). We also did a priori sensitivity multivariable logistic regression analyses and post-hoc descriptive analyses, including analysis with a five-factor health-care facility term instead of a three-factor health-care level term, with the strict WHO CHE definition that includes only direct OOP medical costs (excluding direct OOP non-medical costs) in the numerator and including all costs (direct OOP medical and non-medical costs plus lost income) as per WHO TB Patient Cost Survey methods (appendix 1 pp 39–82).

Data were analysed in STATA (version 15) and R (version 4). Forex exchange rates for 18 June 2022 were applied in this analysis; 1 US$ was equal to 116·4 Kenya shillings, based on the OANDA currency converter.

### Role of the funding source

The funders of the study had no role in study design, data collection, data analysis, data interpretation, or writing of the report.

## Results

In this study, 319 people were invited to participate, of whom 23 (7%) declined (figure). Therefore, 296 participants were included and completed follow-up. However, 26 (9%) participants, all of whom were students, were unable to report household income or expenditure and 21 (7%) of these students were also unable to report household asset data to inform the poverty score.

**Figure:**
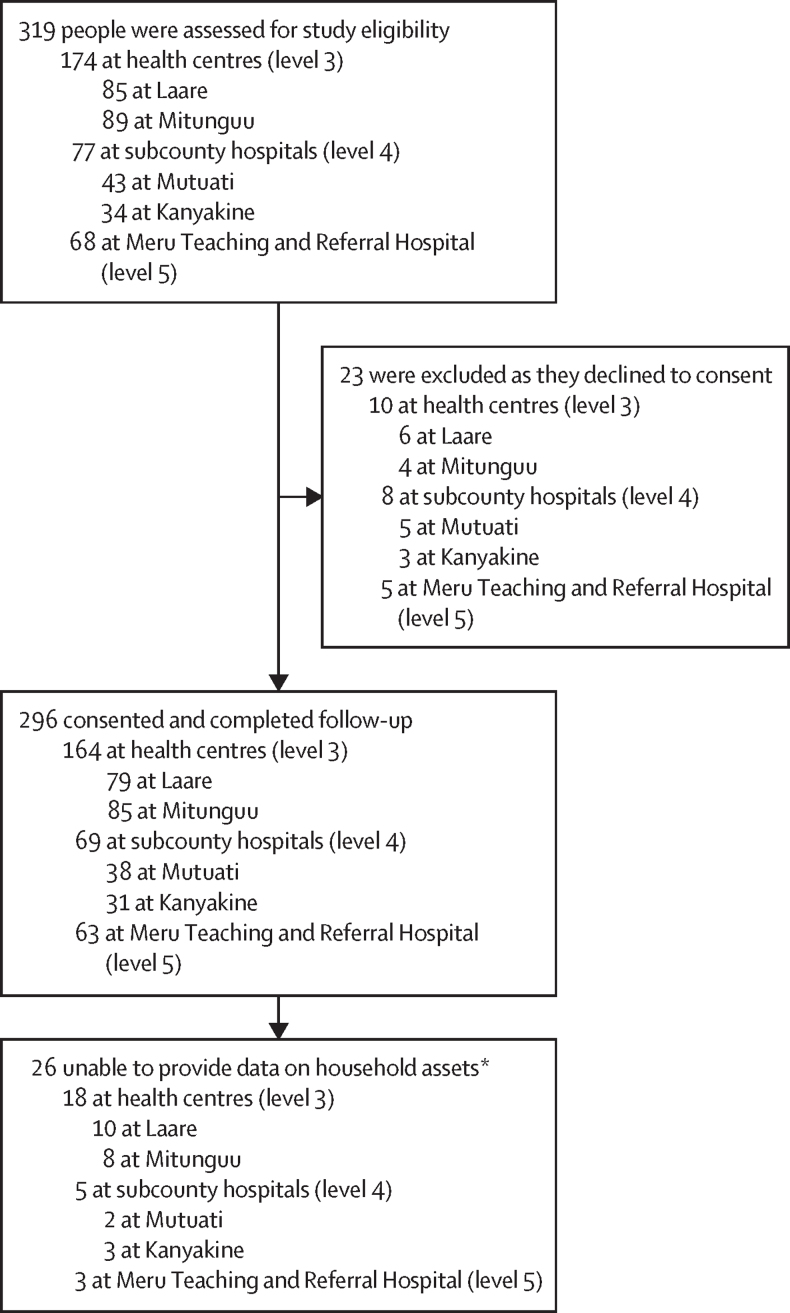
Participant inclusion flowchart *21 of these were students who were also unable to provide data on household assets from which to derive a poverty score. Income, expenditure, and where relevant poverty score were imputed for these 26 participants to provide a full dataset of 296 for analysis of the primary outcome measure of factors associated with catastrophic health expenditure.

Table 1 summarises the social and health characteristics by CHE incurrence. The participants had a median age of 34 years (95% CI 32–36), 173 (58%) of 296 participants were female and 123 (42%) were male, and 163 (55%) had only primary school education. 164 (55%) participants were seen at the health-centre level and 237 (80%) were unaccompanied. Excluding four (1%) participants who were newly diagnosed with tuberculosis, chronic respiratory disease was newly diagnosed in 36 (12%) participants and included 16 (5%) people with asthma, 14 (5%) with chronic bronchitis, two (1%) with COPD, two (1%) with post-tuberculosis lung disease, and two (1%) with bronchiectasis. No participant reported a known chronic respiratory disease diagnosis before their attendance. The mean monthly participant income was 4684 Kenyan shillings (KES), equivalent to US$40 (based on exchange rates on June 26, 2024), and the mean monthly household income was 17 482 KES, equivalent to US$150 (table 1). Appendix 1 (pp 43–51) shows the social and health characteristics of participants by the three health-care levels and five health-care facilities.

**Table 1:** Social and health characteristics of the study cohort by catastrophic health expenditure

		**All participants (n=296)**	**No catastrophic health expenditure (n=220)**	**Catastrophic health expenditure (n=76)**	**p value**
**Social factors**					
Age, years		34 (32–36)	33 (30–36)	37 (32–41)	0·047
Age groups, years		..	..	..	0·0060
	18–29	103 (35%; 30–40)	89 (40%; 34–47)	14 (18%; 11–29)	..
	30–44	117 (40%; 34–45)	78 (35%; 29–42)	39 (51%; 40–62)	..
	45–59	60 (20%;16–25)	41 (19%; 14–24)	19 (25%; 17–36)	..
	≥60	16 (5%; 0·3–8·5)	12 (5%; 3–9)	4 (5%; 2–13)	..
Sex		..	..	..	0·51
	Female	173 (58%; 53–64)	131 (60%; 53–66)	42 (55%; 44–66)	..
	Male	123 (42%; 36–47)	89 (40%; 36–47)	34 (45%; 34–56)	..
Education level		..	..	..	0·90
	Up to primary school completion	163 (55%; 49–61)	121 (55%; 48–61)	42 (55%; 44–66)	..
	Up to secondary school completion	94 (32%; 27–37)	71 (32%; 26–39)	23 (30%; 21–41)	..
	Higher than secondary school*	39 (13%; 10–18)	28 (13%; 9–18)	11 (14%; 8–24)	..
**Economic factors**					
Participant and household income and expenditure					
	Mean monthly participant income in Kenyan shillings	4684 (2633–6734)	4645 (1970–7321)	4794 (2714–6874)	0·56
	Mean monthly participant income in US$	40 (23–58)	40 (17–63)	41 (23–59)	..
	Mean monthly participant household income in Kenyan shillings	17 482 (14 955–20 009)	16 618 (13 829–19 407)	19 982 (14 744–25 220)	0·25
	Mean monthly participant household income in US$	150 (128–172)	143 (119–168)	172 (127–217)	..
	Participant is not primary household income earner	248 (84%; 79–88)	186 (85%; 79–89)	62 (82%; 71–89)	0·55
	Participant is primary household income earner†	48 (16%; 12–21)	34 (15%; 11–21)	14 (18%; 11–29)	..
	Mean monthly household expenditure in Kenyan shillings‡	12 848 (11 923–13 773)	13 815 (12 753–14 878)	10 047 (8780–11 313)	0·0002
Mean monthly household expenditure in US$		110 (102–118)	119 (110–128)	86 (75–97)	..
**Poverty score and NHIF coverage**					
Asset-based poverty score		..	..	..	0·049
	First poverty quintile (least poor)	60 (20%; 16–25)	47 (21%; 16–27)	13 (17%; 10–27)	..
	Second poverty quintile	60 (20%; 16–26)	45 (20%; 16–26)	15 (20%; 12–30)	..
	Third poverty quintile	58 (20%; 15–24)	48 (22%; 17–28)	10 (13%; 7–23)	..
	Fourth poverty quintile	59 (20%; 16–25)	45 (20%; 16–26)	14 (18%; 11–29)	..
	Fifth poverty quintile (most poor)	59 (20%; 16–25)	35 (16%; 12–21)	24 (32%; 22–43)	..
NHIF membership or registration		..	..	..	0·27
	NHIF members or registered	84 (28%; 24–34)	63 (29%; 23–35)	21 (28%; 19–39)	..
	Not NHIF members or registered	212 (72%; 66–76)	157 (71%; 65–77)	55 (72%; 61–81)	..
**Health and health-system factors**					
Accompanied while seeking care					
	No	237 (80%; 75–84)	194 (88%; 83–92)	43 (57%; 45–67)	<0·0001
	Yes	59 (20%; 16–25)	26 (12%; 8–17)	33 (43%; 33–55)	..
Final diagnosis		..	..	..	0·059
	Acute URTI or LRTI	188 (64%; 58–69)	148 (67%; 61–73)	40 (53%; 42–63)	..
	No diagnosis	67 (23%; 18–28)	45 (20%; 16–26)	22 (29%; 20–40)	..
	Asthma	16 (5%; 3–9)	12 (5%; 3–9)	4 (5%; 2–12)	..
	Chronic bronchitis	14 (5%; 3–8)	10 (5%; 2·5–8)	4 (5%; 2–13)	..
	Tuberculosis	4 (1%; 0–3)	2 (1%; 0–3)	2 (3%; 1–9)	..
	COPD	2 (1%; 0–2)	1 (<1%; 0–3)	1 (1%; 0–7)	..
	Post-tuberculosis lung disease	2 (1%; 0–2)	2 (1%; 0–3)	0	..
	Bronchiectasis	2 (1%; 0–2)	0	2 (3%; 1–9)	..
	Congestive cardiac failure	1 (<1%; 0–2)	0	1 (1%; 0–7)	..
Health-system level at which participants are seeking care		..	..		<0·0001
	Health centre (level 3)	164 (55%; 50–61)	153 (70%; 63–75)	11 (14%; 8–24)	..
	Subcounty hospital (level 4)	69 (23%; 19–28)	42 (19%; 14–25)	27 (36%; 26–47)	..
	County hospital (level 5)	63 (21%; 17–26)	25 (11%; 8–16)	38 (50%; 39–60)	..

Data are median (95% CI), mean (95% CI), or n (%; 95% CI). The catastrophic health expenditure threshold is calculated as total health expenditure (total direct medical costs of participant + total direct non-medical costs of participant and carer or accompanying person) of more than 10% of total monthly household expenditure. p values compare participants who had catastrophic health expenditure versus those who did not. URTI=upper respiratory tract infection. LRTI=lower respiratory tract infection. COPD=chronic obstructive pulmonary disease*.* NHIF=National Health Insurance Fund.

* Including higher college and university.

† Individual participant income contributes more than 50% of total household income.

‡ Mean total monthly household expenditure including food, travel, rent, leisure, amenities, and health-care costs of all household members.

For the incidence of CHE, 76 (26%) of 296 participants had CHE. Factors independently associated with CHE included the participant being aged 30–44 years (aOR 2·6, 95% CI 1·3–5·3, p=0·010), being female (1·8, 1·3–2·7, p=0·0021), having education beyond secondary school (1·6, 1·1–2·3, p=0·0083), being accompanied when seeking care (3·2, 1·7–5·9, p<0·0001), belonging to the second poverty quintile (2·0, 1·9–2·1, p<0·0001), and seeking care from subcounty (9·7, 9·6–9·8, p<0·0001) and county hospitals (25·1, 15·7–40·2, p<0·0001; table 2).

**Table 2:** Univariable and multivariable logistic regression of health and social factors associated with catastrophic health expenditure

	**Unadjusted univariable odds ratio (n=296)**	**p value**	**Adjusted multivariable odds ratio (n=296)**	**p value**
**Age groups, years**				
18–29	1 (ref)		1 (ref)	..
30–44	3·7 (2·9–4·6)	0·0010	2·6 (1·3–5·3)	0·010
45–59	3·4 (1·7–6·7)	0·0010	2·3 (0·5–11·0)	0·31
≥60	2·3 (0·7–7·5)	0·18	1·1 (0·2–6·2)	0·95
**Gender**				
Male	1 (ref)	..	1 (ref)	..
Female	0·7 (0·4–2·0)	0·71	1·8 (1·3–2·7)	0·0021
**Education level**				
Up to primary school completion	1 (ref)	..	1 (ref)	..
Up to secondary school completion	1·3 (1·2–1·5)	<0·0001	1·0 (0·5–1·9)	0·95
Above secondary school	1·7 (1·0–2·8)	0·053	1·6 (1·1–2·3)	0·0083
**Smoker**				
Never smoker	1 (ref)	..	..	..
Current or past smoker	0·9 (0·2–3·8)	0·93	..	..
**Primary income earner**				
No	1 (ref)	..	..	..
Yes	1·1 (0·7–1·7)	0·72	..	..
**Accompanied during health-care seeking and visit**				
No	1 (ref)	..	1 (ref)	..
Yes	6·5 (4·1–10)	<0·0001	3·2 (1·7–5·9)	<0·0001
**Asset-based poverty score**				
First quintile (least poor)	1 (ref)	..	1 (ref)	..
Second quintile	1·4 (0·8–2·4)	0·30	2·0 (1·9–2·1)	<0·0001
Third quintile	1·0 (0·8–1·3)	0·91	0·7 (0·6–0·9)	0·0009
Fourth quintile	1·2 (1·0–1·4)	0·13	0·5 (0·2–1·2)	0·11
Fifth quintile (poorest)	2·2 (1·1–4·7)	0·035	2·1 (0·6–7·7)	0·27
**National Health Insurance Fund member**				
Yes	1 (ref)	..	1 (ref)	..
No	1·3 (0·9–1·8)	0·11	1·5 (0·7–3·5)	0·30
**Number of coping strategies used**				
None or single coping strategy used	1 (ref)	..	..	..
Multiple coping strategies used	3·0 (1·4–6·4)	0·0041	..	..
**Diagnosis**				
Any diagnosis	1 (ref)	..	..	..
No diagnosis	1·6 (0·5–5·1)	0·47	..	..
**Health system level at which participant is seeking care**				
Health centre	1 (ref)	..	1 (ref)	..
Subcounty hospital	9·7 (8·4–11·1)	<0·0001	9·8 (9·7–9·9)	<0·0001
County hospital	24 (19·9–28·6)	<0·0001	25·1 (15·7–40·2)	<0·0001

Data are odds ratio (95% CI), adjusted odds ratio (95% CI), or p values. The catastrophic health expenditure threshold is calculated as total health expenditure (total direct medical costs of participant + total direct non-medical costs of participant and carer or accompanying person) of more than 10% of total monthly household expenditure. Interaction testing of factors associated with 10% catastrophic health expenditure showed an interaction between coping strategies and education level and coping strategies was dropped from the multivariable model. These interaction tables are shown in the appendix (pp 37–38).

Table 3 highlights the mean direct OOP medical costs, direct OOP non-medical costs, lost income, and total costs of the study cohort overall and by CHE incurrence. Mean laboratory costs were the largest individual contributors to medical costs (KES 133 of KES 391; 34%) and travel costs were the largest individual contributors to non-medical (KES 399 of KES 425; 94%) costs (table 3).

**Table 3:** Health-care costs, lost income, and catastrophic health expenditure

	**All participants (n=296)**	**No catastrophic health expenditure (n=220)**	**Catastrophic health expenditure (n=76)**	**p value**
**Direct medical costs**				
Consultation costs in Kenyan shillings	82 (74 to 89)	64 (57 to 71)	133 (117 to 150)	<0·0001
Consultation costs in US$	0·6 (0·6 to 0·7)	0·5 (0·5 to 0·6)	1·1 (1·0 to 1·2)	..
Costs of drugs in Kenyan shillings	73 (55 to 91)	50 (34 to 66)	142 (96 to 187)	<0·0001
Costs of drugs in US$	0·6 (0·4 to 0·7)	0·4 (0·3 to 0·6)	1·2 (0·8 to 1·6)	..
Radiography costs in Kenyan shillings	99 (67 to 131)	24 (6 to 41)	318 (221 to 416)	<0·0001
Radiography costs in US$	0·8 (0·5 to 1·0)	0·2 (0·1 to 0·3)	2·7 (1·9 to 3·6)	..
Laboratory costs in Kenyan shillings	133 (94 to 173)	53 (34 to 73)	364 (231 to 498)	<0·0001
Laboratory costs in US$	1·0 (0·7 to 1·4)	0·5 (0·4 to 0·7)	3·1 (2·0 to 4·3)	..
Other procedure costs in Kenyan shillings	3 (0 to 6)	2 (1 to 3)	7 (0 to 19)	0·40
Other procedure costs in US$	0·0 (0·0 to <0·1)	0·0 (0·0 to <0·1)	0·1 (0·0 to 0·2)	..
**Direct non-medical costs**				
Food costs in Kenyan shillings	16 (11 to 22)	15 (9 to 22)	18 (7 to 29)	0·18
Food costs in US$	0·1 (0·1 to 0·2)	0·1 (0·1 to 0·2)	0·1 (0·1 to 0·2)	..
Travel costs in Kenyan shillings	315 (272 to 357)	227 (192 to 263)	569 (470 to 669)	<0·0001
Travel costs in US$	2·4 (2·0 to 2·8)	2·0 (1·6 to 2·3)	4·9 (4·0 to 5·7)	..
Accommodation costs in Kenyan shillings	0 (0 to 1)	1 (0 to 1)	0	0·59
Accommodation costs in US$	<0·1 (0 to <0·1)	<0·1 (0 to <0·1)	0·0	..
Other costs in Kenyan shillings	3 (1 to 4)	2 (1 to 4)	4 (1 to 8)	0·45
Other costs in US$	0·0 (0·0 to <0·1)	0·0 (0·0 to <0·1)	0·0 (0·0 to 0·1)	..
**Direct non-medical costs of the carer or accompanying person**				
Participant not accompanied	237 (80%; 75 to 84)	194 (88%; 77 to 92)	43 (57%; 45 to 67)	<0·0001
Participant accompanied	59 (20%; 16 to 25)	26 (12%; 8 to 17)	33 (43%; 33 to 55)	..
Food costs in Kenyan shillings	6 (2 to 10)	5 (1 to 9)	11 (1 to 21)	0·090
Food costs in US$	0·0 (0·0 to 0·1)	0·0 (0·0 to 0·1)	0·1 (0·0 to 0·2)	..
Travel costs in Kenyan shillings	84 (55 to 113)	31 (14 to 47)	238 (147 to 330)	<0·0001
Travel costs in US$	0·6 (0·4 to 0·8)	0·3 (0·2 to 0·4)	2·0 (1·3 to 2·8)	..
**Total direct medical costs**				
Total direct medical costs in Kenyan shillings	390 (324 to 456)	192 (151 to 233)	964 (797 to 1130)	<0·0001
Total direct medical costs in US$	3·1 (2·6 to 3·6)	1·6 (1·3 to 2·0)	8·3 (6·8 to 9·7)	..
**Total direct non-medical costs (including direct non-medical costs for the carer or accompanying person)**				
Total direct non-medical costs in Kenyan shillings	425 (361 to 489)	280 (232 to 328)	843 (678 to 1008)	<0·0001
Total direct non-medical costs in US$	3·7 (3·1 to 4·2)	2·4 (2·0 to 2·8)	7·2 (5·8 to 8·7)	..
**Total health expenditure**				
Total direct costs in Kenyan shillings	815 (704 to 927)	472 (399 to 546)	1807 (1539 to 2075)	<0·0001
Total direct costs in US$	7·0 (6·0 to 8·0)	4·1 (3·4 to 4·7)	15·5 (13·2 to 17·8)	..
**Lost income and days of work**				
Lost income in Kenyan shillings	247 (153 to 341)	211 (98 to 324)	350 (193 to 506)	<0·0001
Lost income in US$	2·1 (1·3 to 2·9)	1·8 (0·8 to 2·8)	3·0 (1·7 to 4·3)	..
Days of work lost	1·7 (1·5 to 1·9)	1·3 (1·2 to 1·5)	2·8 (2·1 to 3·5)	<0·0001
**Total expenditure (total health expenditure + lost income)**				
Total costs in Kenyan shillings	1062 (896 to 1228)	684 (525 to 843)	2157 (1833 to 2481)	<0·0001
Total costs in US$	9·1 (7·7 to 10·5)	5·9 (4·5 to 7·2)	18·5 (15·7 to 21·3)	..

Data are mean (95% CI) or n (%; 95% CI). The catastrophic health expenditure threshold is calculated as total health expenditure (total direct medical costs of participant + total direct non-medical costs of participant and carer or accompanying person) of more than 10% of total monthly household expenditure. p values compare participants who had catastrophic health expenditure versus those who did not. For non-parametric continuous variables, including direct medical costs of participant (consultation, medicines, radiography, laboratory, or other procedure), direct non-medical costs of participant (food, travel, accommodation, or other, including mobile telecommunications airtime), direct non-medical costs of the carer or accompanying person (food or travel), total direct medical costs of participant, total direct non-medical costs of participant and carer or accompanying person, days of work lost, lost income of participant, and total expenditure, 95% bootstrapped CIs were generated and compared with Mann–Whitney U tests. For the remaining binary categorical variable (participant accompanied) 95% Wilson CIs of proportions were generated and compared with Pearson's χ^2^ test.

Mean total direct costs were KES 815 ($7·0), of which KES 425 (52%) were direct non-medical costs and KES 390 (48%) were direct medical costs. Mean total costs (ie, including lost income) were KES 1062 ($9·1), with mean total direct costs accounting for KES 815 (77%) and mean lost income for KES 247 (23%) of these total costs (table 3).

Participants whose households incurred CHE had higher mean total direct OOP medical costs, direct OOP non-medical costs, lost income, and days of work lost than those who did not (table 3). Appendix 1 (pp 52–58) shows costs and lost income of participants by three health-care levels and five health-care facilities visited.

Table 4 details coping strategies and NHIF coverage and usage. The main coping strategies to pay for care-seeking due to symptoms of chronic respiratory disease were use of savings and borrowing (table 4). Most (282 of 296, 95%) households used at least one coping strategy and 13% used two or more coping strategies (table 4). Less than a third of participants had NHIF membership, of whom only 11 used NHIF to pay for care-seeking costs (table 4). The main reasons stated for not using NHIF were lack of discussion about NHIF at the point of care and ineligibility for cover due to defaulting on monthly premiums (table 4). Among those without NHIF membership, the main reasons reported for non-membership were unaffordability, unawareness of NHIF or eligibility, and lack of information, including regarding enrolment processes (table 4). Having to use two or more coping strategies during illness and care seeking was seen in a higher proportion of participants whose households incurred CHE than participants whose households did not (table 4).

**Table 4:** Coping strategies and NHIF coverage and use by catastrophic health expenditure

		**All participants (%; n=296)**	**No catastrophic health expenditure (n=220)**	**Catastrophic health expenditure (n=76)**	**p value**
**Coping strategies**					
Use savings		176 (59%; 53–64)	123 (56%; 49–62)	50 (66%; 55–75)	0·13
Borrow		91 (31%; 26–36)	63 (29%; 23–35)	28 (37%; 27–48)	0·18
Money from other sources		22 (7%; 5–11)	21 (10%; 6–14)	1 (1%; 0–7)	0·018
Additional work		20 (7%; 4·5–10)	9 (4%; 2–8)	11 (14%; 8–24)	0·0020
Sell property		11 (4%; 2–7)	7 (3%; 2–6)	4 (5%; 2–13)	0·48
Loans		7 (2%; 1–5)	3 (1%; 0–4)	4 (5%; 2–13)	0·075
Default on rent		1 (<1%; 0–2)	0	1 (1·5%; 0–7)	0·26
**Number of coping strategies used**					
None		14 (5%; 3–8)	14 (6%; 4–10)	0	<0·0001
1		245 (83%; 78–87)	187 (85%; 80–89)	58 (76%; 66–84)	..
≥2		37 (13%; 9–17)	19 (9%; 6–13)	18 (24%; 16–34)	..
**NHIF member**					
No		212 (72%; 66–76)	157 (71%; 65–77)	55 (72%; 61–81)	0·27
Yes		84 (28%; 24–34)	63 (29%; 23–35)	21 (28%; 19–39)	..
If yes, was NHIF used to pay for care?					
	Yes, NHIF was used to pay for care	11/84 (13%; 7–22)	7/63 (11%; 5–21)	4/21 (19%; 8–40)	0·49
	No, NHIF was not used to pay for care	73/84 (87%; 78–93)	56/63 (89%; 79–95)	17/21 (81%; 60–92)	..
If no, main reason for not using NHIF to pay for care					
	Did not ask or was not asked about using NHIF	30/73 (41%; 31–53)	26/56 (46%; 34–59)	4/17 (24%; 10–47)	0·039
	Not up to date with NHIF premium payments	22/73 (30%; 21–41)	14/56 (25%; 16–38)	8/17 (47%; 26–69)	..
	Facility not signed up or NHIF card not taken	16/73 (22%; 14–33)	13/56 (23%; 14–36)	3/17 (18%; 6–41)	..
	Forgot card or other required identification	3/73 (4%; 1–11)	3/56 (5%; 2–15)	0/17	..
	Did not know services covered by NHIF	2/73 (3%; 0·5–9)	0/56	2/17 (12%; 3–34)	..
**Monetary value covered by NHIF when used to pay for care**					
Kenyan shillings covered by NHIF		1278 (499–2058)	837 (213–1461)	2050 (338–3762)	0·21
US$ covered by NHIF		11·0 (4·3–17·7)	7·2 (1·8–12·6)	17·6 (2·9–32·3)	..
**Main reason for not having NHIF**					
NHIF is unaffordable		92/212 (43%; 37–50)	65/157 (41%; 34–49)	27/55 (49%; 36–62)	0·49
Unaware of NHIF		56/212 (26%; 21–33)	40/157 (25%; 19–33)	16/55 (29%; 19–42)	..
Lack of information		44/212 (21%; 16–27)	34/157 (22%; 16–29)	10/55 (18%; 10–30)	..
Services or package offered is inadequate		2/212 (1%; 0–3)	2/157 (1%; 0–5)	0/55	..
Choice of facility is limited		0/212	0/157	0/55	..
Other*		18/212 (8%; 5–13)	16/157 (10%; 6–16)	2/55 (4%; 1–12)	..

Data are n (%; 95% CI) or mean (95% CI). The catastrophic health expenditure threshold is calculated as total health expenditure (total direct medical costs of participant + total direct non-medical costs of participant and carer or accompanying person) of more than 10% of total monthly household expenditure. p values compare participants who had catastrophic health expenditure *vs* those who did. NHIF=National Hospital Insurance Fund.

* Still plan to enrol (n=9), issues with required documents (n=2), issues with identity card (n=3), stopped submitting NHIF annual premiums (n=2), or no specific reason (n=2).

Appendix 1 (pp 59–64) shows coping strategies and NHIF coverage and usage of participants by three health-care levels and five health-care facilities visited.

Sensitivity regression analyses of CHE, including other CHE thresholds and separate analysis with health-care levels as a three-factor term and health-care facilities visited as a five-factor term, showed broadly similar associations as those in the primary analyses. A key exception was that when lost income was incorporated into the CHE threshold, 94 (32%) of 296 participants incurred CHE and being the primary income earner in the household was independently associated with incurring CHE (aOR 6·1, 95% CI 3·8–9·7, p<0·0001). Findings of the sensitivity and post-hoc descriptive analyses examining costs at different CHE thresholds, by health-care level or health-care facility visited, and interaction testing are shown in appendix 1 (pp 65–88).

## Discussion

To our knowledge, this is the first study to evaluate the incidence and drivers of CHE and the coverage and use of social protection in people with chronic respiratory disease symptoms in Kenya. CHE incidence in the cohort was high, at 26% of the cohort. Drivers of CHE included being female, belonging to poorer households, being accompanied when seeking care, and seeking higher levels of care such as subcounty or county hospitals. Less than a third of participants were NHIF members and only 13% of those with NHIF used it to pay for health care. These novel findings add to accumulating evidence on the severe socioeconomic effects of seeking and accessing health care in LMICs with little social protection coverage,^29^, ^30^ and will inform ongoing policy dialogue on health financing for NCDs and roll-out of SHIF in Kenya.

Our study found that direct OOP non-medical costs, predominantly related to travel, were the leading contributor (40%) of total expenditure, which mirrors findings from the 2017 Kenyan TB Patient Cost Survey.^30^ However, direct OOP medical costs contributed a substantial proportion of total costs (37%), as has been found in a household survey that estimated that 82% of Kenyans incurred OOP costs to purchase medicines used to treat NCDs.^31^ More specifically, in Meru County, a 2019 study on the self-management of NCDs found that 64% of patients reported difficulties in paying for their medicines.^32^ Although evidence in the field of chronic respiratory diseases in sub-Saharan Africa is scarce,^33^, ^34^, ^35^ our findings suggest the potential benefit of further subsidisation or lower prices for the most widely used medicines for chronic respiratory diseases and consideration of bringing care closer to the point-of-need to reduce travel costs of those seeking care and the people accompanying them.

Despite tuberculosis and chronic respiratory disease sharing similar symptoms and diagnostic pathways, there has been little recognition of the socioeconomic effect or CHE incurrence among the sizeable number of people with symptoms of chronic respiratory disease who seek care and are not subsequently diagnosed with tuberculosis.^36^ Studies from Uganda and Malawi have shown that costs of such care seeking are high.^37^, ^38^ Cost studies in Kenya have focused on cardiovascular diseases, cancer, and diabetes as NCDs rather than chronic respiratory disease.^39^ A single study by Subramanian and colleagues estimated patient costs of screening, diagnosis, and treatment services for all four main NCDs, including chronic respiratory disease, in public and private facilities in Nairobi.^40^ However, the findings of these studies are limited because they focused only on direct OOP medical costs estimated by health-care expert opinion, and based on estimates of drug-distributor prices, and did not collect empirical patient costs data nor consider either OOP non-medical costs or lost income.

Our study identified having lower socioeconomic status, being accompanied when seeking care, seeking care at higher levels of the health service, and being female as key drivers of CHE. The association between household impoverishment and CHE is well described elsewhere.^23^, ^41^ Subramanian and colleagues identified this medical poverty trap in people with NCDs, with the poorest households least likely to have health insurance and most likely to incur CHE.^40^ More broadly, Salari and colleagues used Kenya's 2018 Household Health Expenditures and Utilization Survey to show that 10·7% of Kenyans incurred CHE due to OOP costs of accessing care and estimated that more than 1 million people were pushed into poverty as a result.^29^ Our findings showed that poorer quintiles were more likely to incur CHE than the richest quintile, although the relationship across poverty quintiles did not always reach significance and appeared non-linear. Of note, our sensitivity analyses showed that, compared with including direct costs only, incorporating lost income into the CHE calculation increased the proportion of people with CHE (32% *vs* 26%) and identified being a primary income earner as an additional independent risk factor for CHE. This finding mirrors those of WHO TB Patient Cost Surveys and suggests the importance—in the context of UHC and social protection coverage—of comprehensive CHE measurement, including not only medical costs but also non-medical costs and lost income, to evaluate the economic effect of care-seeking on households.^20^

The finding that being female was associated with CHE highlights sex differentials in health-seeking behaviour, employment (including informal or unpaid jobs), and household or caregiver roles.^42^ The relationship between sex and CHE has been found in other studies, especially those that focus on care-seeking for maternal health,^43^, ^44^, ^45^, ^46^ and warrants additional investigation and consideration of a gender-responsive approach to the design, delivery, and evaluation of health and social protection strategies.

This study identified accessing health-care services at subcounty hospitals and the more distant but better resourced county hospital as predictors of CHE. Compared with primary health-care facilities, such hospitals have wider catchment areas, resulting in higher travel costs and can also charge for tests in addition to sputum tests for tuberculosis, such as other laboratory tests, radiology services, and drugs, which might be required for chronic respiratory disease diagnosis and treatment.^14^ These findings support further research to map and potentially redesign patient care pathways to directly reduce health-care costs at the point of access and complementary socioeconomic support strategies to mitigate OOP medical and non-medical costs of people seeking care for chronic respiratory disease symptoms, especially the most vulnerable.

Coverage of NHIF was low at 28%, which is lower than Meru County as a whole (33%) but similar to national coverage (27%; appendix 1 p 6). However, even in those covered by NHIF, use of NHIF to pay for health-care services was negligible. Little information and awareness, including about NHIF eligibility and processes, and issues with affordability and maintaining premiums were cited as the main barriers to NHIF membership and usage. For example, voluntary membership in the NHIF cost KES 500 per month ($3·9). Although family NHIF cover was available at other rates, this cost translates to about 10% of the mean monthly individual income (KES 4684, $40·0) for the study participants, and could be unaffordable to many people in similar contexts. Similar findings have previously been reported in Kenya and call into question the prospects of NHIF, and now SHIF in its current form, as the preferred vehicle for UHC nationally.^47^ The findings of this study are timely in considering ease of SHIF registration, coverage, and access to health-care services, especially for those in the informal labour market, as it continues to be rolled out. Indeed, there are increasing calls to identify alternative mechanisms for financial protection—eg, moving from contributory health insurance to tax-funded health-care purchasing models.^13^, ^48^

This study has several limitations. First, it was a cross-sectional study of a single care-seeking episode in adults with chronic respiratory disease symptoms of any reported duration, with sampling across study sites during standard opening hours, limiting generalisability. Our sample was 58% female with median age of 34 years, which is not dissimilar to participant demographics in studies of health-care seeking behaviour in Nairobi.^49^, ^50^ In the TB Patient Cost Surveys, household-level costs showed gender differentials driven by disparities in the economic position of men and women.^51^ Second, we were unable to estimate the costs or coping strategies of people with symptoms of chronic respiratory disease over time, many of whom need long-term management.^36^ Third, we did not recruit severely ill participants or stratify our analysis by disease severity, which could potentially affect the level of care sought. However, data were collected from consecutive eligible patients at each study site on actual costs incurred for that facility visit, minimising recall bias.^52^ Fourth, in line with WHO's TB Patient Cost Survey methods,^51^ we used multiple imputation with chained equations to estimate income, expenditure, and poverty score in 26 participants with missing data. Fifth, calculations did not consider lost income of caregivers or people accompanying patients, which could have underestimated cost contributions. However, we did include non-medical costs of accompanying people, which are considered essential to measure, have been included in other CHE thresholds such as WHO's TB Patient Costs Survey,^20^ and are recognised as a hidden cost and an important driver of CHE, as shown in our sensitivity analyses (appendix 1 pp 65–83).^53^ Sixth, the COVID-19 pandemic and a health-care workers’ strike disrupted health-care service provision and data collection for 6 months and both cost data and chronic respiratory disease symptoms and attendances could have been influenced by COVID-19 and seasonal variation.^54^, ^55^

Our novel findings suggest a sizeable burden of CHE and use of financial coping strategies in people seeking care for chronic respiratory disease symptoms in Meru County, Kenya, driven by sex and socioeconomic inequalities and differential access to health care and social protection. The findings suggest a pressing need to reconsider strategies aimed at optimising coverage and usage of the new SHIF programme (which will probably have similar access issues) and tackling NCDs, mitigating CHE, and working towards achieving UHC in Kenya.

### NIHR International Multidisciplinary Programme to Address Lung Health and TB in Africa Consortium

### Contributors

### Equitable partnership declaration

### Data sharing

Individual de-identified participant data are not available as permission for secondary data analyses was not specified in the original consent forms and application for ethics approval.

## Declaration of interests

TW is supported by grants from the Wellcome Trust, UK (209075/Z/17/Z); the Medical Research Council, Department for International Development, and Wellcome Trust (Joint Global Health Trials, MR/V004832/1); the Medical Research Council (PHIND, MR/Y503216/1); and the Medical Research Foundation (Dorothy Temple Cross International Collaboration Research Grant, MRF-131–0006-RG-KHOS-C0942) and receives consultancy fees from WHO for ad hoc consultancy related to tuberculosis. MT is a trustee on a registered UK charity called National Friendship Fund but does not receive fees or honoraria for this role. All other authors declare no competing interests.

## Supplementary Materials

Supplementary appendix 1

Equitable Partnership Declaration

## References

[1] WHO Noncommunicable diseases. https://www.who.int/news-room/fact-sheets/detail/noncommunicable-diseases#:~:text=Cardiovascular%20diseases%20account%20for%20most,of%20all%20premature%20NCD%20deaths.

[2] WHO Monitoring health for the SDGs. https://www.who.int/health-topics/sustainable-development-goals#tab=tab_2.

[3] Lagomarsino G, Garabrant A, Adyas A, Muga R, Otoo N (2012). Moving towards universal health coverage: health insurance reforms in nine developing countries in Africa and Asia. Lancet.

[4] Kenyan Ministry of Health (2014). National Strategic Plan for Tuberculosis, Leprosy and Lung Health, 2015–2018. https://nltp.co.ke/wp-content/uploads/2020/10/M-and-E-plan-2015–2018.pdf.

[5] Kenyan Government. Constitution of Kenya. 2010.

[6] WHO (2023). Global health expenditure database. https://apps.who.int/nha/database/Select/Indicators/en.

[7] Chuma J, Musimbi J, Okungu V, Goodman C, Molyneux C (2009). Reducing user fees for primary health care in Kenya: policy on paper or policy in practice?. Int J Equity Health.

[8] WHO (2005).

[9] McIntyre D (2012). What healthcare financing changes are needed to reach universal coverage in South Africa?. S Afr Med J.

[10] WHO, International Bank for Reconstruction and Development (2021).

[11] Kenyan Presidency (2022). The “Big Four”—immediate priorities and actions. https://kenyaembassy.nl/wp-content/uploads/2021/07/Government-of-Kenya-Big-Four-Agenda.pdf.

[12] The National Hospital Insurance Fund (2023). The UHC Supacover. https://www.nhif.or.ke/uhc-supa-cover/.

[13] Barasa E, Rogo K, Mwaura N, Chuma J (2018). Kenya National Hospital Insurance Fund Reforms: implications and lessons for universal health coverage. Health Syst Reform.

[14] Barasa EW, Maina T, Ravishankar N (2017). Assessing the impoverishing effects, and factors associated with the incidence of catastrophic health care payments in Kenya. Int J Equity Health.

[15] McCollum R, Theobald S, Otiso L (2018). Priority setting for health in the context of devolution in Kenya: implications for health equity and community-based primary care. Health Policy Plan.

[16] Kenyan Ministry of Health (2014). Kenya health policy 2014–2030: towards attaining the highest standard of health. https://repository.kippra.or.ke/handle/123456789/4681.

[17] Ministry of Health (2017). Annual report 2017: national tuberculosis, leprosy and lung disease program. https://nltp.co.ke/wp-content/uploads/2020/10/NTLP_Annual_Report_2017_Portrait_Final.pdf.

[18] Byrne AL, Marais BJ, Mitnick CD, Lecca L, Marks GB (2015). Tuberculosis and chronic respiratory disease: a systematic review. Int J Infect Dis.

[19] WHO (2012). ENGAGE-TB: integrating community-based tuberculosis activities into the work of nongovernmental and other civil society organizations. https://iris.who.int/bitstream/handle/10665/75997/9789241504508_eng.pdf.

[20] WHO (2017).

[21] Kirubi B, Ong'ang'o J, Nguhiu P, Lönnroth K, Rono A, Sidney-Annerstedt K (2021). Determinants of household catastrophic costs for drug sensitive tuberculosis patients in Kenya. Infect Dis Poverty.

[22] Gama E, Madan J, Langley I (2016). Economic evaluation of a shortened standardised treatment regimen of antituberculosis drugs for patients with multidrug-resistant tuberculosis (STREAM): study protocol. BMJ Open.

[23] Wingfield T, Tovar MA, Huff D (2016). The economic effects of supporting tuberculosis-affected households in Peru. Eur Respir J.

[24] Barber JA, Thompson SG (1998). Analysis and interpretation of cost data in randomised controlled trials: review of published studies. BMJ.

[25] Hsu J, Flores G, Evans D, Mills A, Hanson K (2018). Measuring financial protection against catastrophic health expenditures: methodological challenges for global monitoring. Int J Equity Health.

[26] Jolliffe IT, Cadima J (2016). Principal component analysis: a review and recent developments. Philos Trans A Math Phys Eng Sci.

[27] McDonald JH (2009). Handbook of biological statistics.

[28] Bursac Z, Gauss CH, Williams DK, Hosmer DW (2008). Purposeful selection of variables in logistic regression. Source Code Biol Med.

[29] Salari P, Di Giorgio L, Ilinca S, Chuma J (2019). The catastrophic and impoverishing effects of out-of-pocket healthcare payments in Kenya, 2018. BMJ Glob Health.

[30] Kenyan Ministry of Health. The first Kenya tuberculosis patient cost survey. National Tuberculosis, Leprosy, and Lung Diseases Program, 2017.

[31] Vialle-Valentin CE, Serumaga B, Wagner AK, Ross-Degnan D (2015). Evidence on access to medicines for chronic diseases from household surveys in five low- and middle-income countries. Health Policy Plan.

[32] Kim E, Ndege PK, Jackson E, Clauw DJ, Ellingrod VL (2019). Patient perspectives on medication self-management in rural Kenya: a cross-sectional survey. Int J Qual Health Care.

[33] Ait-Khaled N, Enarson DA, Bissell K, Billo N (2007). Access to inhaled corticosteroids is key to improving quality of care for asthma in developing countries. Allergy.

[34] Kibirige D, Kampiire L, Atuhe D (2017). Access to affordable medicines and diagnostic tests for asthma and COPD in sub Saharan Africa: the Ugandan perspective. BMC Pulm Med.

[35] Ozoh OB, Eze JN, Garba BI (2021). Nationwide survey of the availability and affordability of asthma and COPD medicines in Nigeria. Trop Med Int Health.

[36] Squire S, Thomson R, Namakhoma I, El Sony A, Kritski A, Madan J (2015). Catastrophic care-seeking costs as an indicator for lung health. BMC proc.

[37] Samuels THA, Shete PB, Ojok C (2021). Where will it end? Pathways to care and catastrophic costs following negative TB evaluation in Uganda. PLoS One.

[38] Sichali JM, Khan JA, Gama EM (2019). Direct costs of illness of patients with chronic cough in rural Malawi—Experiences from Dowa and Ntchisi districts. PLoS One.

[39] Oyando R, Njoroge M, Nguhiu P (2020). Patient costs of diabetes mellitus care in public health care facilities in Kenya. Int J Health Plann Manage.

[40] Subramanian S, Gakunga R, Kibachio J (2018). Cost and affordability of non-communicable disease screening, diagnosis and treatment in Kenya: patient payments in the private and public sectors. PLoS One.

[41] Xu K, Evans DB, Carrin G, Aguilar-Rivera AM, Musgrove P, Evans T (2007). Protecting households from catastrophic health spending. Health Aff.

[42] Pan American Health Organization (2021). Out of pocket expenditure in health: the need for a gender analysis. https://www.paho.org/en/documents/out-pocket-expenditure-need-gender-analysis.

[43] Bonu S, Bhushan I, Rani M, Anderson I (2009). Incidence and correlates of ‘catastrophic’ maternal health care expenditure in India. Health Policy Plan.

[44] Myint ANM, Liabsuetrakul T, Htay TT, Wai MM, Sundby J, Bjertness E (2018). Impoverishment and catastrophic expenditures due to out-of-pocket payments for antenatal and delivery care in Yangon region, Myanmar: a cross-sectional study. BMJ Open.

[45] Mahajan N, Kaur B (2021). Analysing the expenditure on childbearing: a community-based cross-sectional study in rural areas of Punjab (India). BMC Health Serv Res.

[46] Arsenault C, Fournier P, Philibert A (2013). Emergency obstetric care in Mali: catastrophic spending and its impoverishing effects on households. Bull World Health Organ.

[47] Mathauer I, Schmidt JO, Wenyaa M (2008). Extending social health insurance to the informal sector in Kenya. An assessment of factors affecting demand. Int J Health Plann Manage.

[48] Olalere N (2021). NHIF bill amendments: the devil is in the details. https://www.businessdailyafrica.com/bd/opinion-analysis/ideas-debate/nhif-bill-amendments-the-devil-is-in-the-details-3454578.

[49] Fonck K, Mwai C, Ndinya-Achola J, Bwayo J, Temmerman M (2002). Health-seeking and sexual behaviors among primary healthcare patients in Nairobi, Kenya. Sex Transm Dis.

[50] Otieno PO, Wambiya EO, Mohamed SM (2020). Access to primary healthcare services and associated factors in urban slums in Nairobi-Kenya. BMC Public Health.

[51] WHO (2022). National surveys of costs faced by tuberculosis patients and their households 2015–2021. https://www.who.int/publications/i/item/9789240065536.

[52] Jager KJ, Tripepi G, Chesnaye NC, Dekker FW, Zoccali C, Stel VS (2020). Where to look for the most frequent biases?. Nephrology.

[53] Wingfield T, Boccia D, Tovar M (2014). Defining catastrophic costs and comparing their importance for adverse tuberculosis outcome with multi-drug resistance: a prospective cohort study, Peru. PLoS Med.

[54] Ivey M, Simeon D, Monteil MA (2003). Climatic variables are associated with seasonal acute asthma admissions to accident and emergency room facilities in Trinidad, West Indies. Clin Exp Allergy.

[55] Johnston N, Sears M (2006). Asthma exacerbations . 1: epidemiology. Thorax.

