# Supplementary material for: Catastrophic health expenditure, social protection coverage, and financial coping strategies in adults with symptoms of chronic respiratory diseases in Kenya: a cross-sectional study
Source: Lancet Glob Health. 2025 Jun 25;13(7):e1301–13. doi: 10.1016/S2214-109X(25)00061-0 (PMC12208783; doi:10.1016/S2214-109X(25)00061-0)
Supplement: Supplementary appendix 1 [file mmc1.pdf]

THE LANCET
Global Health

Supplementary appendix 1

This appendix formed part of the original submission and has been peer reviewed. We post it as supplied by the authors.

Supplement to: Mulupi S, Waithera C, Tomeny EM, et al. Catastrophic health expenditure, social protection coverage, and financial coping strategies in adults with symptoms of chronic respiratory diseases in Kenya: a cross-sectional study. *Lancet Glob Health* 2025; **13:** e1301–13.

Supplementary file to Mulupi *et al* original research article:

"Catastrophic health expenditure, social protection coverage, and financial coping strategies amongst adults with symptoms of chronic respiratory diseases in Kenya: a cross-sectional study"

Published in Lancet Global Health

Table of Contents

Supplementary File 1: Kenya health system context and Meru County sociodemographic and health information 4

Supplementary Figure 1: Maps of Kenya and (inset) Meru County and selected study site subcounties . 5

Supplementary Table 1: Trends in key demographic and healthcare indicators in Meru County and at the national level between 2012-2015 6

Supplementary File 2: Adaptation and piloting of the costing tool 7

Supplementary File 3: Patient journey mapping tool for clinical consultation and health facility service access 9

Supplementary File 4: Exit interview questionnaires 20

Supplementary File 5: Details of Poverty score derived from Principal Component Analysis (PCA) 36

Supplementary File 6: Interaction outputs corresponding to Table 4 from main manuscript using Stata mfpigen function 37

Supplementary File 7a: Table showing mean direct medical costs amongst only participants who received or completed the test or procedure 39

Supplementary File 7b: Table showing median healthcare costs, lost income, and catastrophic health expenditure (n=296) 40

Supplementary File 8: Sociodemographic and clinical characteristics, costs and coping strategies by three healthcare levels visited and by five healthcare facilities visited 42

Supplementary Table 1a: Sociodemographic and health characteristics of the study cohort and by three healthcare levels (n=296) 43

Supplementary Table 1b: Sociodemographic and health characteristics of the study cohort and by five healthcare facilities visited (n=296) 47

Supplementary Table 2a: Healthcare costs, lost income, and catastrophic health expenditure by three healthcare levels visited (n=296) 52

Supplementary Table 2b: Healthcare costs, lost income, and catastrophic health expenditure by five healthcare facilities visited (n=296) 55

Supplementary Table 3a: Coping strategies and NHIF coverage and use by healthcare level visited (n=296) 59

Supplementary Table 3b: Coping strategies and NHIF coverage and use by five healthcare facilities visited (n=296) 62

Supplementary File 9: A priori sensitivity univariable and multivariable logistic regression analyses 65

Supplementary Table 4: Univariable and multivariable logistic regression of health and social factors associated with catastrophic health expenditure including by five healthcare facilities 66

Supplementary Table 5: “Medical costs only" sensitivity analysis showing univariable and multivariable logistic regression of health and social factors associated with catastrophic health expenditure by three health system levels 71

Supplementary Table 6: "Medical costs only" sensitivity analysis showing univariable and multivariable logistic regression of health and social factors associated with catastrophic health expenditure by five healthcare facilities 74
Supplementary Table 7: Total costs sensitivity analysis showing univariable and multivariable logistic regression of health and social factors associated with catastrophic health expenditure by healthcare level 77
Supplementary Table 8: Total costs sensitivity analysis showing univariable and multivariable logistic regression of health and social factors associated with catastrophic health expenditure by five healthcare facilities 80
Supplementary File 10: post-hoc exploratory, descriptive analysis 83
Supplementary Figure 2a: Catastrophic health expenditure at 10% of total monthly household expenditure threshold by facility level 83
Supplementary Figure 2b: Mean direct medical costs, direct non-medical costs, and lost income of participants by facility level 84
Supplementary Figure 2c: Direct medical costs, direct non-medical costs, and lost income as a proportion of total costs of participants by facility level 85
Supplementary Figure 3a: Catastrophic health expenditure at 10% of total monthly household expenditure threshold by healthcare facility 86
Supplementary Figure 3b: Mean direct medical costs, direct non-medical costs, and lost income of participants by healthcare facility 87
Supplementary Figure 3c: Direct medical costs, direct non-medical costs, and lost income as a proportion of total costs of participants by healthcare facility 88
References 89

Supplementary File 1: Kenya health system context and Meru County sociodemographic and health information

Following constitutional reforms in 2010, the Kenyan governance system was devolved with the subsequent devolution of healthcare services being one of the most significant and visible changes. Fiscal and administrative decision-making powers for health service provision became vested on the 47 subnational governments (counties). The devolved public health care delivery system is structured across six levels: Level 1 – community; Levels 2 and 3 – primary care, comprising dispensaries, and health centres, respectively providing basic outpatient services and some inpatient services for expectant women; Level 4 – Subcounty hospitals, which are the primary referral hospitals in the devolved system provide radiological services and a wider range of laboratory services, including more sensitive TB diagnostic services, with certain level 4 facilities providing sputum testing services using Gene Xpert technology; Level 5 – County referral hospitals serve as the apex referral facilities for the entire county region. Specialised care (medical outpatient clinics, MOPC) is provided at the hospital levels, including physician consultation and medical outpatient clinics on specific weekdays for chronic conditions like cardiovascular, cancer and diabetes, but – unless in the uncommon instance of being a specific “chest clinic” - not other conditions such as CRDs.

The subnational county governments are mandated to provide healthcare services from community level to Level 5 County referral hospitals. They budget for and oversee Level 1 to 3 services, often making challenging choices about what conditions and geographic areas are prioritised for services.[15] Healthcare services at the county level are managed through the County Department of Health (CDOH). County and subcounty health management teams oversee all the planning and decision-making and provision of support to health facilities at the county and subcounty levels. Level 6 – national referral hospitals and other specialised facilities, e.g. blood transfusion services, national laboratory services, are managed by the national government, which is constitutionally mandated to design policies, develop the capacity of health care workers and provide technical assistance to counties.

Meru County, located in Central Kenya, has a population of approximately 1.5 million people.[1] Meru County has nine administrative subcounties (Supplementary Figure 1), each with its own subcounty Health Management team. Meru County has 15 Subcounty hospitals and 1 County hospital. Estimated travel time by public transport from study site health centres or Subcounty hospitals to the County hospital was approximately 1-3 hours.

Health facilities were sampled in Igembe North (Laare Health Centre and Mutuati Subcounty Hospital); Meru Teaching and Referral Hospital (Imenti North), and Mitunguu Health Centre and Kanyakine Subcounty Hospital (South Imenti Subcounty). Health centres were selected based on closest proximity to the Subcounty hospitals. The Subcounty hospitals were selected because they had TB diagnostic services including Gene Xpert and tested samples collected in local healthcare facilities. Meru Teaching and Referral Hospital (MTRH), the only regional hospital and the highest healthcare service referral level in Meru County, serves patients referred from lower-level health facilities.

Supplementary Figure 1: Maps of Kenya and (inset) Meru County and selected study site subcounties

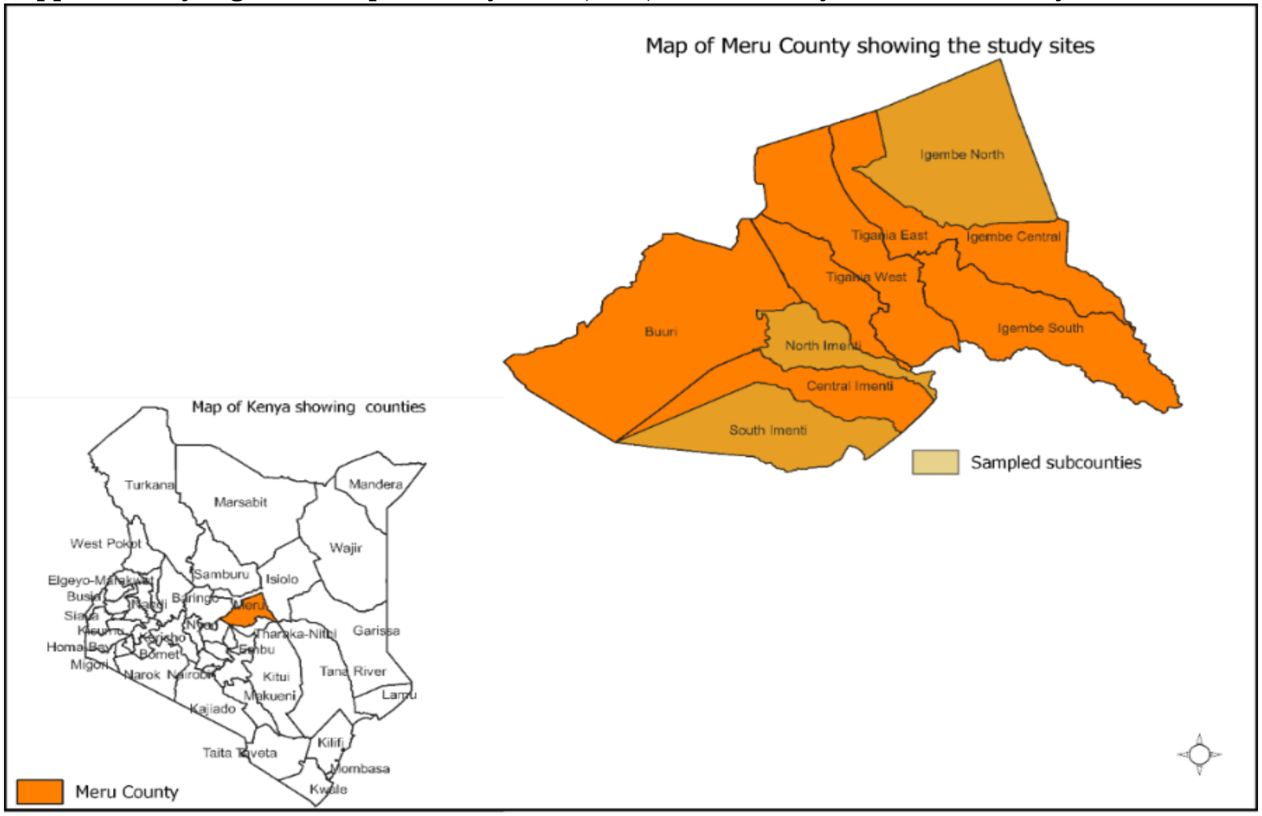

According to Kenyan government policy, sputum microscopy for TB diagnosis and TB drugs for treatment are provided free of charge through a vertical programme subsidy in all public facilities.[2] Patients attending dispensaries and health centres are only required to pay for consultation fees and other laboratory tests. At the hospital level all other services apart from sputum tests, are charged. Chest radiograph and spirometry are only available at hospital level.

Meru County has endemic poverty with few households having electricity and most using open fires for cooking. Despite this, the burden of chronic respiratory diseases (CRD) in Meru, remains unknown. The trends in key demographic and healthcare indicators in Meru County and at the national level in Kenya (2012-2015)[3], are shown in Supplementary Table 1 below.

Supplementary Table 1: Trends in key demographic and healthcare indicators in Meru County and at the national level between 2012-2015

Population	Meru County, 2012	Meru County, 2015	National estimate, Kenya, 2015
Total Population	1,488,984	1,584,575	45,108,414
Male	736,264	783,532	22,422,667
Female	752,720	801,403	22,685,747
Children aged under five years old	212,925	226,594	6,936,691
Tuberculosis			
TB prevalence per 100,000 people	153	219	208
TB incidence per 100,000 people	78	117	79
Health facilities			
Public	103	139	4929
Non-governmental	2	5	347
Faith-based	56	58	1081
Private for profit	204	258	3797
Health personnel in public facilities			
Nurses per 100,000 people	55	66	55
Doctors per 100,000 people	15	17	10
Clinical officers per 100,000 people	14	22	21
Health financing			
Total government health spending per capita (KES)	923	806	1585
National Hospital Insurance Fund coverage as % of population	25	32.6	26.7

Supplementary File 2: Adaptation and piloting of the costing tool

Patients' data were collected first, by trained clinical officers who identified eligible participants during routine history-taking and physical examination, with each consenting participant assigned a unique ID. Twelve Clinical Officers were trained (Health Centre, n=2; Subcounty hospitals n=6; MTRH n=4). Seven of the clinical officers were female. The trainings were conducted in each facility, prior to data collection, over two consecutive days. Each training session was conducted between 7:00-8:00am to minimise disruptions in healthcare service delivery. During the training, the healthcare workers were issued with paper questionnaires containing details of the information needed and provided guidance on how to implement the questionnaires and complete data collection. The healthcare workers had opportunities to ask questions, which were clarified before data collection started. Clinical data based on history and physical examination were collected in consultation rooms using standard questionnaires **(Supplementary File 3).**

Clinical officers identified eligible participants presenting to healthcare services and linked them to a research field worker for consenting. They filled data for all consenting patients in the standard questionnaires **(Supplementary File 3)**. Six trained field workers (2 males, 4 female) administered exit interviews. Field worker trainings covered data collection procedures, effective interviewing skills, ethical conduct of scientific research, and data management, including quality assurance processes. Sociodemographic and clinical data were collected. Clinical data included CRD symptoms, clinical officer-defined diagnosis based on history and physical examination, and any recommended tests, investigations, or treatment.

On the same day, at the point of the participants leaving the facility, the field workers administered the remainder of the survey focused on participant costs of accessing care. The exit interview surveys lasted, on average, 22 minutes. Each participant was provided travel reimbursement of 300 Kenyan Shillings (USD 1.8), an amount which has been used before in study sites and is known to be locally acceptable and appropriate. Exit interview questionnaires (see **Supplementary File 4**) were adapted from the WHO TB Patient Costs Survey instrument[4], which uses an itemised costing approach and was previously adapted and used in the Kenya National TB Patient Cost Survey.[5] Participants estimated the: direct (out-of-pocket) medical costs, direct non-medical out-of-pocket costs, lost income, and coping strategies associated with their CRD symptoms, illness, and concomitant care-seeking; and the coverage and use of NHIF. Direct medical out-of-pocket costs included those for medical consultations, diagnostic tests (e.g., laboratory and radiological imaging), medicines, and any other medical procedures (e.g., bronchoscopy, pulmonary function testing). Direct non-medical out-of-pocket costs included one-way travel to the health centre, food, and accommodation, and were collected for both the participants and any persons accompanying them during healthcare seeking and facility visits.

Other data collected included socio-demographics, age, gender, occupation, educational attainment, monthly income, and monthly participants' household income, and coverage by and use of NHIF to pay for medical expenses during the specific healthcare facility visit. In order to generate a poverty score (see **Supplementary File 5**), we collected data on asset ownership including household furniture, electronic equipment, livestock, and amenities such as electricity and piped water; estimates of weekly household incomes. Household income, expenditure and consumption on food, rent, travel, amenities, and leisure, were also collected to inform the denominator used to calculate CHE incurrence.

The exit interview questionnaires (n=18) were piloted in Mutionjiri health centre and Miathene Subcounty hospitals, both in non-study subcounties. Additionally, clinical officers in all sampled facilities piloted the tools on patients (n=20), providing feedback to the study team. Data were collected using SAMSUNG Galaxy tablets (Model SM-T580) and transmitted on the same day to a secure server on the Open Data Kit (ODK) platform. All data were reviewed by the Principal Investigator (SM) within a day of collection to ensure accuracy.

Supplementary File 3: Patient journey mapping tool for clinical consultation and health facility service access

1.0	Date of interview	DAY [__][__] MONTH [__][__]<br>Year[__][__][__][__]
1.1	Interviewer code / Initials	[__][__]
1.2	Name of facility (facility code)	( )
1.3	Sub-county name (code)	( )

Confirm inclusion criteria:

1.4. Please confirm these details about patients

**Does the patient have any of these symptoms?**	**Yes**	**No**	
Cough	1	2	
Difficulty breathing	1	2	
Wheezing	1	2	
Chest pains	1	2	
Blood in sputum	1	2	

Proceed to consenting

**INTRODUCTION**: Hello, we are doing a study to see how much time it takes patients who have lung problems to go through all the service points within this facility. Our staff would like to record the time taken for each activity you go through today till you leave the facility to go home. Should you leave the facility for another activity, e.g. to go to the shops or other services e.g. x-ray, we will also record the time it takes before you resume other services at the facility. **We will <u>NOT</u> take your name, test results, or any personal information.** You can identify our staff by the green name badges they are wearing. If you need care tomorrow, we wish to record the time you will take in getting various services again. If you do not with to participate in this activity, please feel free to say so. This will not affect the care you receive in this facility today or any other day.
May I record the time it takes to complete the activities you go through at the facility today?

1- Yes 2-No			
**2. STATION 1:**			
**2.1. ID number**<br>*Give patient ID badge*	___ ___ ___	**Date (dd/mm/yy)**	___ ___/___ ___/___ ___
**2.2. Time of arrival:**	___ ___: ___ ___	**2.3. Time of departure**	___ ___: _____ _____
**2.4. Purpose of visit:**	**___ ___**<br>**2.5. Respiratory symptoms**<br>**NOTES:**	**2.6. Age of patient** (write the age)<br>☐☐<br>**2.7. Gender:**<br>1- Male<br>2- Female	**2.8.** Patient is alone/ accompanied?<br>1- Alone<br>2- Accompanied by another person(s) - specify number
**Station 1**	**RECEPTION**	**Indicate station number:**	
**Step 1**	**Enter activity e.g. payment/ registration/**		
TIME IN	___:___	TIME OUT	_____:____
Payment for outpatient services/ card<br>Confirm from Appendix 3	Enter all activities involving patient care in step 1 (E.g. recording name)		

b, all the activities involved at this station			
**STEP 2**	**Enter activity e.g. Triaging**		
TIME IN	___:___	TIME OUT	___:___
Registration	Enter all activities that happen in step 2.		
**Station 2**	**CONSULTATION- please indicate room number**		
TIME IN	___:___		______:_____
Please indicate the following<br>2.1. History of cough:<br>1- <2 weeks<br>2- 2-7 weeks<br>3- 8 weeks<br>4- >8 weeks<br>2.2. Physical examination: Other symptoms<br>1- Difficulty in breathing<br>2- Wheeze			

3- Chest tightness

4- Blood in sputum

67_Other (please specify)

2.3. Diagnosis made at **this** first consultation?

1- Yes

2- No (skip to 2.6)

2.4. Specify diagnosis

1- TB

2- Post-TB lung disease

3- Asthma

4- COPD/ Emphysema

5- Bronchiectasis

6- Acute causes

67__Other (Specify)

2.5. Was patient prescribed medication?

1- Yes (Specify________________,________________, ______________ skip to 2.8.)

2- No

2.6. Any tests recommended?

1- Yes

2- No (Skip to 2.8)

2.7. Tests recommended

1- Sputum test

2- Chest x-ray

3- CT scan

67_ Other-specify

2.8. Other decision made?

1- Referral to another service point within facility (please specify)

2- Referral to other facility e.g. for x-ray (please retain this form)

3- Admission

67_ Other (specify)

2.9. Was client given counselling/ health education?

1- Yes

2- No

TIME OUT___:____

**STATION 3 TEST: LAB (please indicate room number)**			
TIME IN			

3.1. Tests conducted:

1- Gene Xpert

2- AFB test

67_Other (Specify0

3.2. Is the gene Xpert test conducted at the healthcare facility or referred to another facility?

1- Conducted at facility

2- Referred (specify date when sample is likely to be dispatched_____/_____/2019)

**If Gene Xpert is conducted out of the facility, please retain this questionnaire and inform the research team member.**<br><br>TIME OUT____:_____			
**STATION 4 TEST: X-RAY (please indicate room number)**			
TIME IN	___:____		
This is applicable only to patients taking x-rays within the facility.<br><br>Was x-ray conducted<br>1- Yes<br>2- No<br><br>TIME OUT_______:_________<br>**Please indicate where patient is referred next**			
**STATION 5: DIAGNOSIS- : please Indicate room number-**			
TIME IN			
**This is only applicable to patients sent for tests**<br><br>Please indicate date of diagnosis____/_____/_____ (Day/Month/ Year)<br><br>**5.1. For Xray tests and Gene Xpert tests, please indicate date when the test was done, based on documentation from Xray or lab**			

1- Date of x-ray

2-Date of Gene Xpert test

3-Date of AFB test

5.2. Specify diagnosis

1- TB
2- Post-TB lung disease
3- Asthma
4- COPD/ Emphysema
5- Bronchiectasis
6- Acute causes

67__Other (Specify)

5.3. Was patient prescribed medication?

1- Yes (Specify_______________,_______________, ________________)
2- No

5.4. Any **further** tests recommended?

1- Yes (Specify)
2- No (Please indicate the next service point for patient)

5.5. Other decision made?

1- Referral to other facility(specify)
2- Referral to Medical Officer/ Pediatrician for further consultation (**Applicable only to Sub county and Meru Teaching and Referral Hospital)**
3- Admission

67_ Other (specify)

TIME OUT_____________:__________

6. Medical Officer/ Pediatrician Only(please indicate room number)

TIME IN_________:________

6.1. Please describe the differential diagnosis for this case

6.2. Any further tests recommended?

1- Yes
2- No (skip to 6.3)

Please specify

6.3. Medical prescription?

Please list prescribed medication

6.4. Is patient referred to another service point within facility?

1- Yes (specify_____________________________)
2- No

TIME OUT___________:__________

6.5 NOTE FOR CONSULTATION

Please note final decision made about patient:

1: Await test results (Date?)

2: Diagnosis made

3: Treatment decision

a. Prescribe drugs for facility pharmacy
b. Referral to another facility (indicate name of facility)

5: Next appointment (Date)

6: Linkage to CHV?

STATION 7: PHARMACY

Please indicate Room number:

FIRST ENCOUNTER- CONFIRMATION OF DRUGS IN STOCK

**TIME IN**	**____:_____**	

Pharmacy:

7.1. Please list all drugs prescribed for patient

**Drugs prescribed**	**Availability**<br>1- Yes<br>2- No
1.	
2.	
3.	

4.	

TIME OUT_____:_____________

8. CASH OFFICE

TIME IN	_________:_______		

**Drugs purchased**	Cost
1.	
2.	
3.	
4.	
Total cost	

TIME OUT____________:__________

**9. PHARMACY**	**COLLECTION OF DRUGS**		
TIME IN	________:________ _		

<table>
<tr><td colspan="4">EXIT POINT

Confirm that this is the last service point for the patient today.

Please note the time and, request the patient to leave the tool with you.</td></tr>
<tr><td>TIME OF EXIT</td><td></td><td></td><td></td></tr>
<tr><td colspan="2"></td><td></td><td></td></tr>
</table>

Supplementary File 4: Exit interview questionnaires

INTRODUCTION SECTION: Health facility and patient details

**0.1. Sub county name [code]**	
**0.2. Date of interview**	**[dd/mm/ yyyy]**
0.3. Day of the week	1- Monday<br>2- Tuesday<br>3- Wednesday<br>4- Thursday<br>5- Friday<br>6- Saturday
**0.4. Health Facility Name [code]**	**1-Meru Teaching and Referral Hospital**<br>**2-Kanyakine Subcounty Hospital**<br>**3-Mitunguu Health Centre**<br>**4-Mutuati Subcounty Hospital**<br>**5-Laare Health Centre**
**0.5. Category of treating facility:**<br>**1. Health centre**<br>**2. Subcounty hospital**<br>**3. County hospital**	
**0.6. Patient name**	
**0.7. Patient registration number in facility register**	
**0.8. Sex: 1. Male 2. Female**	
**0.9. Age of patient (years)**	
**0.10. Area of residence:**<br>**1- Sub-count**<br>**2- Location**<br>**3- Sublocation**	

**0.11.**	**Time of interview [Start/ End]**	
**0.12.**	**Interviewer name (Code)**	
**0.13.**	**Results Code**	Completed- 01<br>Partially completed- 02

SECTION 1: SOCIO-DEMOGRAPHIC DATA

1.1.	What is your marital status	1- Monogamous married<br>2- Married Polygamous<br>3- Living together<br>4- Separated<br>5- Divorced<br>6- Widow/ widower<br>7- Never married
1.2.	What level of education did you reach, and grade completed?	1- Pre-primary<br>2- Primary<br>3- Post Primary, vocational<br>4- Secondary / A level<br>5- College/ middle level<br>6- University undergraduate<br>7- University, postgraduate<br>8- Madrassa<br>96—Other (Specify)<br>99__Don't know<br>For all current students (1,2,3, 4 skip to section 2)

1.3.	What type of work do you do as your main job/ business (the last 7 days?) **Describe** the main occupation in at least 2 words e.g. Primary school teacher, General shopkeeper, college teacher, computer programmer etc.	(Write a description here)<br>(For student, skip)
1.4.	Who is your main employer for the job/ business?	1- National government agencies<br>2- County government<br>3- Private sector enterprise<br>4- International organizations/ NGO<br>5- Faith-based organization<br>6- Self-employed- modern<br>7- Self- employed- informal<br>8- Small scale agriculture (employed)<br>9- Self-small-scale agriculture<br>10- Pastoralist activities (employed)<br>11- Self-pastoralist activities<br>12- Individual/ private household<br>13- Schools' board of employees<br>96_Other-specify
1.5.	What is your working pattern in your main job/ business?	1- Regular (skip to 1.8)<br>2- Casual
1.6.	For how many days did you do casual job in the last one month?	Specify
1.7.	What is the average daily wage you received during the days	(Estimate cash value for any in-kind payment received)

	worked as casual labour in the last one month?	
1.8.	How much were you paid as salary the last one month?	Specify
1.9.	On average, how much money in Kenyan shillings, does your household spend per week on	1- Food<br>2- Rent<br>3- Travelling<br>4- Medication<br>5- Leisure<br>Other, specify
1.10.	Does your household have any of the following? (tick all that apply)	1. A table<br>2. A chair<br>3. A sofa<br>4. A bed<br>5. A cupboard<br>6. A clock<br>7. A non-mobile telephone<br>8. A mobile telephone<br>9. A radio<br>10. Electricity (If no, skip to question 1.11)<br>11. A solar panel<br>12. A television<br>13. A refrigerator<br>14. A microwave oven<br>15. A DVD player<br>16. A cassette or CD player

1.11.	What means of transport do you mainly use?	1- Walk<br>2- Bicycle/ Motorbike/ bodaboda<br>3- Own bicycle<br>4- Tuk tuk<br>5- Matatu<br>6- Bus<br>7- Employer-provided<br>8- Private vehicle<br>9- Not applicable
1.12.	Do you own agricultural land?	1- Yes<br>2- No
1.13.	How many acres of land do you own?	1- Acres (specify)<br>2- Plot size (Square feet)<br>99_Don't Know
1.14.	Do you own any farm animals	1- Yes<br>2- No
1.15.	Which farm animals do you own (specify number)?	1- Local cattle<br>2- Exotic/ grade cattle<br>3- Horses/Donkeys<br>4- Goats/sheep/pigs<br>5- Chicken/ turkeys/ ducks<br>96_Other- specify
1.16.	Do you own your current dwelling?	1- Yes<br>2- No

1.17.	Do you own the land on which the dwelling is built?	1- Yes<br>2- No
1.18.	Does your household receive any form of assistance/ cash transfer from the government?	1- Yes<br>2- No (Skip to Section 2)<br>99_dont know (Skip to section 2)
1.19.	For what reason does your household receive a cash transfer or social assistance?	1- Orphaned children 18 years and below<br>2- Elderly persons<br>3- Person with severe disability<br>4- Urban food subsidy<br>5- Health voucher<br>6- Food/ cash for work<br>7- School feeding<br>8- Hunger/ safety net program<br>96_Other (Specify)

SECTION 2: DECISION TO ATTEND HEALTH FACILITY AND DIAGNOSTIC SERVICES RECEIVED

2. **This section contains questions about the experiences of patients with chronic cough in the healthcare facility.**

2.1.	How long have you been coughing, or had difficulty in breathing before coming here today?	1- Less than 2 weeks<br>2- 2-4 weeks<br>3- More than 4 weeks<br>99_ Don't know
2.2.	Is this the first time you have visited a health facility because of coughing or breathing difficulty??	1- Yes (skip to 2.4.)<br>2- No
2.3.	Where else did you go, for healthcare before coming to this facility, for this coughing or breathing problem?	1- Self-medication (drugs over the counter, borrow friends or relatives)

			2- Visited another public facility<br>3- Visited a private facility<br>4- Visited traditional doctor<br>5- Faith healing<br>6- CHV<br>96_Other (specify)	
2.4.	Who advised you to come to this facility?		1- Made own decision<br>2- Friends/ family<br>3- A community health volunteer/ worker<br>4- Referral from another facility<br>96_ Other (specify)	
**Experience with diagnostic tests**				
2.5.	For this problem (CRS), were you asked to undergo any tests?		1- Yes<br>2- No (Skip to section 3)	
2.6.	Please explain whether you have been tested			
	Test	Test requested<br>1- Yes<br>2- no	Test completed<br>1- Yes<br>2- No	Reasons for not completing<br>1- Cannot afford<br>2- Service not available in facility<br>3- Have been referred to another facility<br>4- Don't think it is important<br>96_ Other- specify

	1. Sputum tests			
	2. Chest X ray			
	3. CT scan			
	4. Blood test			
	5. Spirometry			
	6. Other			
2.7.	Have you received the results of the test?			
	Test	1- Yes	2-No (Skip to 2.11)	
	1. Sputum tests			
	2. Chest x-ray			
	3. CT scan			
	4. Blood tests			
	5. Spirometry			
	6. Other			
2.8.	Did the doctor say what the diagnosis was?			
	1. Yes<br>2. No (skip to 3.1.)			
2.10.	What was the diagnosis?			
	1. TB<br>2. Post-TB lung disease<br>3. Asthma<br>4. COPD/ Emphysema<br>5. Bronchiectasis<br>6. Acute causes<br>7. Other (specify)			
2.11.	If ‘No’ in 2.7, did someone in this facility tell you when you should expect to receive the results for the tests that you have done today for your chest/ breathing problem?			
	1- No they didn’t tell me			

	2- Later today<br>3- Tomorrow<br>4- Within a week<br>5- More than a week<br>96_Other, specify	

SECTION 3: EXPERIENCE ABOUT TREATMENT RECEIVED FOR THIS HOSPITAL VISIT

3.1.	Did you receive any treatment today for this breathing/ coughing problem?	
	1- Yes<br>2- No [skip to 3.3.]	
3.2.	What treatment did you receive in this facility for your breathing/ coughing problem?	
	1- Drugs for TB treatment<br>2- Other antibiotics<br>3- Bronchodilators<br>4- Inhaled steroids<br>5- Oral steroids<br>6- Inhalers<br>96_ Other (Specify)	
3.3.	What decision has been made about your breathing or coughing problem?	
	1- Have been asked to come back another day (skip to 3.5)<br>2- Have been referred to another facility<br>96_Other (specify)	
3.4.	If referred to another facility, where have you been referred to?	
	1- Public subcounty hospital	

	2- Public county referral hospital<br>3- Private provider facility<br>4- Private, retail pharmacy<br>5- Other<br>(Skip to 3.7)	
3.5.	Do you plan to come back to this facility on the appointed day?	
	1- Yes (Skip to section 4)<br>2- No<br>99_ Don't know	
3.6.	If 'No' in 3.5, what are the main reasons you may not come back on the appointed day?	
	1- I would have other things to do (e.g. work, travel)<br>2- I cannot afford fare<br>3- It depends on partners' decision<br>4- I don't think it is necessary/ hope to feel better<br>5- I plan to go elsewhere<br>96_Other (Specify)	
3.7.	Do you plan to seek healthcare anywhere for this coughing/ breathing problem?	
	1- Yes<br>2- No	
3.8.	Where else do you plan to seek for healthcare?	
	1- Public facility<br>2- Private facility<br>3- Retail pharmacy<br>4- Traditional doctor<br>5- Self-medication<br>6- Faith healing	

	96_Other- specify	
3.9.	Why do you prefer the provider mentioned in 3.8 above?	
	1- Availability of diagnostic tests<br>2- Availability of medicines<br>3- Cheaper prices<br>4- Closer to home<br>5- Availability of doctors<br>96_Other (specify)	

SECTION 4: OWNERSHIP OF HEALTH INSURANCE AND PAYMENT FOR EXPENSES INCURRED FOR THIS HEALTH FACILITY VISIT

4.1.	Are you enrolled in any health insurance scheme?	
	1- Yes<br>2- No (skip to 4.3)	
4.2.	Which insurance scheme are you enrolled in?	
	1- National Hospital Insurance Fund (NHIF)<br>2- Community Based Health Insurance (CBHI)<br>3- Private health insurance schemes<br>4- NHIF + other insurance (applicable to either Private or CBHI)<br>96_Other (Specify)<br><br>(Skip to 4.5)	
4.3.	Do you know if you are eligible for NHIF?	
	1- Yes<br>2- No (Skip to 4.5)	

4.4.	Why don’t you have NHIF insurance?<br>1- Lack of information<br>2- NHIF is unaffordable<br>3- Services offered in the benefit package are not adequate<br>4- Choice of facility is limited<br>5- Other

Direct Medical costs incurred for this health facility visit

4.5. Please describe the expenses that you have incurred for this health facility visit.

**4.5.1. Medical out-of-pocket payments, (Total per visit)**<br>**(A)**					
Consultation fee<br><br>A1	Lab tests<br><br>A2	Radiography and other imaging<br><br>A3	Other procedures[1]<br><br>A4	Medicines<br><br>A5	Medical payments, total in Shillings<br><br>(sum) ΣA1 to 5<br><br>(If patient does not remember specifics of A1 to A5, indicate the **TOTAL** amount spent)

[1] **Based on the facility survey, indicate any other procedures that the patient undergoes**

Direct non-medical expenses for hospital visit for patients/ persons accompanying them

**4.5.2. Non-medical out-of-pocket payments for patient (Total per visit)**<br><br>**(B)**				
Trave<br>l<br><br>B1	Food<br>costs<br>during<br>visit<br><br>B2	Accommodation<br><br>B3	Other<br>expenses<br>e.g.<br>phone<br>services<br><br>B4	Non-<br>medical<br>out-of-<br>pocket<br>payments<br>(Total)<br><br>ΣB1 to 4<br><br>(If patient<br>does not<br>remember<br>specifics<br>of A1 to<br>A5,<br>indicate<br>the<br>**TOTAL**<br>amount<br>spent)

**4.5.3. Non-medical out-of-pocket payments for person accompanying patient, (Total per visit)**<br><br>**(C)**				
Trave<br>l<br><br>C1	Food<br>costs<br>durin<br>g visit	Accommodatio<br>n<br><br>C3	Other<br>expenses<br>e.g.	Non-<br>medical<br>out-of-<br>pocket

	C2		phone services<br><br>C4	payments (Total)<br><br>ΣC1 to C4<br><br>(If patient does not remember specifics of C1 to C4, indicate the **Total** amount spent

Payment for Healthcare Services using NHIF (Skip to 4.5.8, for those without NHIF)

4.5.4.	(For those with NHIF)<br><br>Did you use NHIF to cover for any medical expenses for this coughing/ breathing problem?	Instruction: emphasis should be for this hospital visit
	1- Yes<br><br>2- No (Skip to 4.5.7)	
4.5.5.	Which services were paid for by the NHIF?	(Specify amount)
	1- Consultation<br><br>2- Tests (lab)<br><br>3- X-ray / CT scan	

	4- Medicines<br><br>Don't know	
4.5.6.	In total, how much money was paid for by the NHIF?	(Specify amount)<br><br>**Skip to section 4.6 (Economic costs)**
4.5.7.	Why do you think your medical expenses for this hospital visit were not covered by the NHIF?	
	1- Not fully paid up<br>2- Forgot card/ other identification requirements at home<br>3- Did not ask/ was never asked for card<br>4- Didn't know services covered by NHIF<br>5- Other- specify	

4.5.8. Total Direct Expenses incurred for this treatment episode (This section to be filled by the field worker based on responses indicated in Questions 4.5.1. to 4.5.6.).

Out-of-pocket payments (A+B+ C)<br><br>(Gross)<br><br>(D)	Health Insurance reimbursement or waiver<br><br>(E)	Net out of Pocket payments for the treatment episode (Difference between Gross OOP and Heath insurance reimbursement (D-E)
Total out-of-pocket payments<br><br>(ΣA1 to 6) + (ΣB1 to 4) + (ΣC1 to 4)	Health insurance reimbursement, specify:<br><br>1- NHIF<br>2- Private<br>3- Community-based<br>4- Waivered	

4.5.9. Impact of facility visit on individual and household income:

a) What is your household's daily income? *(Explain household to mean all the people who live under one room and share meals from one pot).*

b) How many days of your individual income would you estimate this visit cost you?

c) Have you had to do any of the following to pay for this health facility visit ?

Actions taken to pay for Hospital visit	1- Yes<br>2- No
1- Use savings	
2- Take out a loan	
3- Borrow money	
4- Sell property e.g. household item, livestock or other asset	
5- Do additional work to earn some more money	
6- Default on regular payment (e.g. child school fees)	

Supplementary File 5: Details of Poverty score derived from Principal Component Analysis (PCA)

Eleven asset and utility variables from the latest published Kenya National Household Survey were included in the study and considered by the research team for inclusion in the Principal Component Analysis (PCA). All variables were binary and did not require normalization, transformation or standardization. Ownership of a particular asset and/or utility was given the value "1" and lack of ownership was given the value "0", with greater ownership of assets or utilities assumed to indicate lower poverty and higher wealth. The 11 asset and utility variables included ownership of a table, a chair, a sofa, a bed, a cupboard, a clock, a non-mobile fixed-line phone, a mobile phone, a radio, household access to electricity, a solar panel, a television, a refrigerator, a microwave, a DVD player, a cow, and an exotic cow.

We followed recommended steps to perform the PCA[6, 7] including generating a covariance matrix, creating and evaluating Principal Components and corresponding Eigenvalues, measuring sampling adequacy, revising and restricting included variables to improve sampling adequacy, and converting the selected Principal Component into a poverty score.

First, a covariance matrix of all 11 variables was generated, which although heterogeneous, showed high levels of correlation.

Second, an eigen decomposition of the covariance matrix was created to generate Principal Components (Eigenvectors) and Eigenvalues. Principal Components were shown in descending order of their Eigenvalues with only Principal Components with an Eigenvalue of more than 1 being considered as a suitable Component. In this initial analysis, seven Principal Components had Eigenvalues over one, representing nearly two thirds of the sample variance (Cumulative proportional variance 0.6514).

Third, a Kaiser-Meyer-Olkin measure of sampling adequacy[7] was performed, which showed an unacceptably low overall adequacy of 0.6931 with some individual variables having sampling adequacy below <0.5.

Fourth, based on this inadequate sampling, it was decided to restrict the number of included assets and utility variables to improve the sampling adequacy to an acceptable level (≥0.7). This was done iteratively until a reduced selection of variables with the highest sampling adequacy was reached. The final six asset and utility variables included in the PCA were a sofa, a cupboard, a clock, a radio, a television, and a DVD player. Principal component 1 had an Eigenvalue of 2.65 and accounted for nearly half of the sample variance (Cumulative proportional variance 0.4416). Principal components 2 to 6 all had Eigenvalues of less than one indicating unsuitability to be considered as Principal Components. Kaiser-Meyer-Olkin measures of sampling adequacy were good for each individual item (sofa = 0.8191, cupboard = 0.8399, clock = 0.8188, radio = 0.8233, television = 0.7791, DVD player = 0.7954) and demonstrated a good overall sampling adequacy of 0.8097.

Finally, Principal Component One of this final PCA was converted to a continuous wealth score variable, which was inverted to become a poverty score (i.e. higher scores associated with more poverty/impoverishment) and further converted into poverty quintiles to facilitate inclusion as an independent variable in univariable and multivariable logistic regression analyses and aid interpretation of the regression model outputs, including by policy makers and implementers.[8, 9]

Supplementary File 6: Interaction outputs corresponding to Table 4 from main manuscript using Stata mfpigen function

An interaction between dissaving and gender, healthcare facility level, and education level was identified and dissaving was subsequently not included in the multivariable regression model.

variable 1	function 1	variable 2	function 2	dev. diff.	d.f.	P	Sel
i.agegroups	Factor	i.genderfe~e	Factor	2.7076	2	0.2583	6
	Factor	i.educatio~l	Factor	3.7759	6	0.7070	6
	Factor	i.finalpov~e	Factor	9.3352	9	0.4069	6
	Factor	i.patient_~d	Factor	4.0356	3	0.2576	6
	Factor	i.insuranc~r	Factor	3.2060	3	0.3609	6
	Factor	i.multiple~g	Factor	4.4166	2	0.1099	6
	Factor	i.facility~l	Factor	4.8855	6	0.5586	6
i.genderfe~e	Factor	i.educatio~l	Factor	0.5614	2	0.7553	6
	Factor	i.finalpov~e	Factor	6.1698	4	0.1868	6
	Factor	i.patient_~d	Factor	0.3587	1	0.5492	6
	Factor	i.insuranc~r	Factor	0.4347	1	0.5097	6
	Factor	i.multiple~g	Factor	4.0325	1	0.0446	6
	Factor	i.facility~l	Factor	0.3038	2	0.8591	6
i.educatio~l	Factor	i.finalpov~e	Factor	5.4931	8	0.7038	6
	Factor	i.patient_~d	Factor	1.8577	2	0.3950	6
	Factor	i.insuranc~r	Factor	3.2509	2	0.1968	6
	Factor	i.multiple~g	Factor	7.9953	2	0.0184	6
	Factor	i.facility~l	Factor	1.1447	4	0.8871	6
i.finalpov~e	Factor	i.patient_~d	Factor	4.8609	4	0.3019	6
	Factor	i.insuranc~r	Factor	3.1784	4	0.5284	6
	Factor	i.multiple~g	Factor	2.0107	4	0.7338	6
	Factor	i.facility~l	Factor	9.0179	7	0.2514	6
i.patient_~d	Factor	i.insuranc~r	Factor	1.9110	1	0.1668	6
	Factor	i.multiple~g	Factor	0.0082	1	0.9279	6
	Factor	i.facility~l	Factor	1.4163	2	0.4925	6
i.insuranc~r	Factor	i.multiple~g	Factor	0.0448	1	0.8324	6
	Factor	i.facility~l	Factor	2.7621	2	0.2513	6
i.multiple~g	Factor	i.facility~l	Factor	7.5197	1	0.0061	6

Legend: agegroups = age groups, genderfe~e = gender, educatio~l = education level, facility~l = three-factor healthcare facility level term, finalpov~e = poverty quintile, patient_~d = patient accompanied by carer, insuranc~r = insurance coverage, multiple~g = multiple dissaving

Thhe interaction test was run again removing dissaving and showed no further significant interactions between the independent variables:

variable 1	function 1	variable 2	function 2	dev. diff.	d.f.	P	Sel
i.agegroups	Factor	i.genderfe~e	Factor	2.6365	2	0.2676	5
	Factor	i.educatio~l	Factor	4.0565	6	0.6690	5
	Factor	i.finalpov~e	Factor	9.0399	9	0.4336	5
	Factor	i.patient_~d	Factor	3.8824	3	0.2744	5
	Factor	i.insuranc~r	Factor	3.1980	3	0.3621	5
	Factor	i.facility~l	Factor	4.3864	6	0.6245	5
i.genderfe~e	Factor	i.educatio~l	Factor	0.9005	2	0.6375	5
	Factor	i.finalpov~e	Factor	6.0595	4	0.1947	5
	Factor	i.patient_~d	Factor	0.2190	1	0.6398	5
	Factor	i.insuranc~r	Factor	0.4095	1	0.5222	5
	Factor	i.facility~l	Factor	0.4012	2	0.8182	5
i.educatio~l	Factor	i.finalpov~e	Factor	5.3784	8	0.7165	5
	Factor	i.patient_~d	Factor	1.8672	2	0.3931	5
	Factor	i.insuranc~r	Factor	3.1187	2	0.2103	5
	Factor	i.facility~l	Factor	1.3969	4	0.8447	5
i.finalpov~e	Factor	i.patient_~d	Factor	4.5362	4	0.3383	5
	Factor	i.insuranc~r	Factor	2.2800	4	0.6844	5
	Factor	i.facility~l	Factor	9.3889	7	0.2259	5
i.patient_~d	Factor	i.insuranc~r	Factor	1.6062	1	0.2050	5
	Factor	i.facility~l	Factor	1.1923	2	0.5509	5
i.insuranc~r	Factor	i.facility~l	Factor	2.9546	2	0.2283	5

Legend: facility~l = three-factor healthcare level term, agegroups = age groups, genderfe~e = gender, educatio~l = education level, finalpov~e = poverty quintile, patient_~d = patient accompanied by carer, insuranc~r = insurance coverage

Supplementary File 7a: Table showing mean direct medical costs amongst only participants who received or completed the test or procedure

Direct medical costs of participants who received or completed test or procedure (95%CI)	All participants (n=296)	Catastrophic health expenditure		
		No (n=220)	Yes (n=76)	p value
Mean consultation costs in Kenya Shillings (n=261)	92 (85-100)	81 (73-89)	126 (110-143)	<0.001
Mean consultation costs in USD (n=296)	0.7 (0.6-0.8)	0.6 (0.5- 0.7)	1.0 (0.9- 1.1)	
Mean costs of drugs in Kenya Shillings (n=82)	265 (225-304)	250 (210-291)	289 (210-367)	0.44
Mean costs of drugs in USD (n=82)	2.1 (1.8-2.4)	2.0 (1.6-2.4)	2.3 (1.6-2.9)	
Mean radiography costs in Kenya Shillings (n=38)	774 (686-861)	721 (683-760)	804 (664-944)	0.59
Mean radiography costs in USD (n=38)	6.0 (5.4-6.7)	5.6 (5.3-5.9)	6.3 (5.2-7.4)	
Mean laboratory costs in Kenya Shillings (n=95)	415 (311-519)	291 (230-353)	564 (351-777)	0.0042
Mean laboratory costs in USD (n=95)	3.2 (2.4-4.1)	2.3 (1.8-2.8)	4.4 (2.7- 6.1)	
Mean other procedure costs in Kenya Shillings (n=8)	106 (5.2-207)	50 (50-50)	500 (500-500)	0.0082
Mean other procedure costs in USD (n=8)	0.82 (0.04-1.6)	0.4 (0.4-0.4)	3.9 (3.9-3.9)	

Supplementary File 7b: Table showing median healthcare costs, lost income, and catastrophic health expenditure (n=296)

	**All participants (n=296)**	**Catastrophic health expenditure**		
		**No (n=220)**	**Yes (n=76)**	**p value**
Direct medical costs of participant cohort in Kenya Shillings (IQR)				
Median consultation costs	50 (50-100)	50 (50-100)	150 (50-200)	<0.001
Median costs of drugs	0 (0-45)	0 (0-0)	15 (0-235)	<0.001
Median radiography costs	0 (0-0)	0 (0-0)	0 (0-700)	<0.001
Median laboratory costs	0 (0-120)	0 (0-0)	300 (0-400)	<0.001
Median other procedure costs	0 (0-0)	0 (0-0)	0 (0-0)	0.40
Direct non-medical costs of participant cohort				
Median food costs	0 (0-0)	0 (0-0)	0 (0-0)	0.18
Median travel costs	200 (100-400)	200 (60-400)	400 (200-750)	<0.001
Median accommodation costs	0 (0-0)	0 (0-0)	0 (0-0)	0.59
Median other costs	0 (0-0)	0 (0-0)	0 (0-0)	0.45

Direct non-medical costs of carer/accompanying person across participant cohort				
Median food costs	0 (0-0)	0 (0-0)	0 (0-0)	0.090
Median travel costs	0 (0-0)	0 (0-0)	0 (0-400)	<0.001
Total direct medical costs of participant cohort				
Median total direct medical costs	95 (50-500)	50 (70-200)	815 (400-1300)	<0.001
Total direct non-medical costs of participant cohort (total direct non-medical costs of participant cohort and carer/accompanying person)				
Median total direct non-medical costs	230 (120-514)	200 (80-400)	620 (400-1200)	<0.001
Total health expenditure of participant cohort (total direct medical costs of participant cohort + total direct non-medical costs of participant cohort and carer/accompanying person)				
Median total direct costs	480 (210-1005)	325 (160-635)	1685 (890-2400)	<0.001
Lost income and days of work of participant cohort				
Median lost income	27 (25-123)	25 (12-74)	74 (25-238)	<0.001
Total expenditure of participant cohort (total health expenditure of participant cohort + lost income of participant cohort)				
Median total costs	591 (262-1274)	418 (195-799)	1767 (1058-2819)	<0.001

Supplementary File 8: Sociodemographic and clinical characteristics, costs and coping strategies by three healthcare levels visited and by five healthcare facilities visited

Supplementary Table 1a: Sociodemographic and health characteristics of the study cohort and by three healthcare levels (n=296)

	All participants (n=296)	Participants seeking care at health centre, Level 3 care (n=164)	Participants seeking care at Subcounty Hospital, Level 4 care (n=69)	Participants seeking care at County Hospital, Level 5 care (n=63)	p value
Sociodemographic factors					
Age in years					
Median	34	33	35	35	0.11
(95% CI)	(32-36)	(29-36)	(30-40)	(30-40)	
Age groups					
18-29 years	103 / 296	69 / 164	19 / 69	15 / 63	0.04
% (95%CI)	35 (30-40)	42 (35-50)	28 (18-39)	24 (15-36)	
30-44 years	117 / 296	57 / 164	30 / 69	30 / 63	
% (95%CI)	40 (34-45)	35 (28-42)	43 (32-55)	48 (36-60)	
45-59 years	60 / 296	30 / 164	15 / 69	15 / 63	
% (95%CI)	20 (16-25)	18 (13-25)	22 (14-33)	24 (15-36)	
60 years and above	16 / 296	8 / 164	5 / 69	3 / 63	
% (95%CI)	5 (0.3-8.5)	5 (2.5-9)	7 (3-16)	5 (2-13)	
Gender (%, 95% CI)					
Female	173 / 296	112 / 164	34 / 69	27 / 63	<0.001
% (95%CI)	58 (53-64)	68 (61-75)	49 (38-61)	43 (31-55)	
Male	123 / 296	52 / 164	35 / 69	36 / 63	
% (95%CI)	42 (36-47)	32 (25-39)	51 (39-72)	57 (45-69)	
Education level					

Up to primary school completion	163 / 296	96 / 164	40 / 69	27 / 63	0.13
% (95%CI)	55 (49-61)	59 (51-66)	58 (46-69)	43 (31-55)	
Up to secondary school completion	94 / 296	49 / 164	21 / 69	24 / 63	
% (95%CI)	32 (27-37)	30 (23-37)	30 (21-42)	38 (27-50)	
Above secondary school*	39 / 296	19 / 164	8 / 69	12 / 63	
% (95%CI)	13 (10-18)	12 (8-17)	12 (6-21)	19 (11-30)	
Mean monthly participant income**	4684	2295	3105	12630	0.0014
(95%CI)	(2633-6734)	(1316-3274)	(1590-4620)	(3651-21609)	
Mean monthly participant household income**	17482	13697	11690	33679	<0.001
(95% CI)	(14955-20009)	(11168-16225)	(9413-13966)	(25467-41892)	
Participant is not primary household income earner***	248 / 296	145 / 164	56 / 69	47 / 63	0.42
% (95%CI)	84 (79-88)	88 (83-93)	81 (70-89)	75 (63-84)	
Participant is primary household income earner	48 / 296	19 / 164	13 / 69	16 / 63	
% (95%CI)	16 (12-21)	12 (7.5-17)	19 (11-30)	25 (16-37)	
Mean monthly household expenditure****	12848	12205	11246	16276	<0.001
(95%CI)	(11923-13773)	(11087-13323)	(9475-13017)	(14085-18469)	
Asset-based poverty score					
First quintile (least poor)	60 / 296	35 / 164	14 / 69	11 / 63	0.069
% (95%CI)	20 (16-25)	21 (16-28)	20 (12-31)	17 (10-29)	
Second quintile	60 / 296	41 / 164	14 / 69	5 / 63	

% (95%CI)	20 (16-25)	25 (19-32)	20 (12-31)	7.9 (3.4-17)	
Third quintile	58 / 296	36 / 164	15 / 69	7 / 63	
% (95%CI)	20 (15-24)	22 (16-29)	22 (14-32)	11 (5.5-21)	
Fourth quintile	59 / 296	24 / 164	11 / 69	24 / 63	
% (95%CI)	20 (16-25)	15 (10-21)	16 (9.0-26)	38 (27-50)	
Fifth quintile (most poor)	59 / 296	28 / 164	15 / 69	16 / 63	
% (95%CI)	20 (16-25)	17 (12-24)	22 (14-33)	25 (16-37)	
NHIF membership/registration					
NHIF members/registered	84 / 296	67 / 223	17 / 73	47 / 143	0.063
% (95%CI)	28 (24-34)	30 (24-36)	23 (15-34)	33 (26-41)	
NHIF not members/registered	212 / 296	156 / 223	56 / 73	96 / 143	
% (95%CI)	72 (66-76)	70 (64-76)	77 (66-85)	67 (59-74)	
Health factors					
Accompanied while seeking care					
No	237 / 296	151 / 164	53 / 69	33 / 63	<0.001
% (95%CI)	80 (75-84)	92 (87-95)	77 (66-85)	52 (40-74)	
Yes	59 / 296	13 / 164	16 / 73	30 / 63	
% (95%CI)	20 (16-25)	8 (5-13)	23 (15-34)	48 (36-60)	
Final diagnosis					
Acute URTI or LRTI	188 / 296	126 / 164	39 / 69	23 / 63	<0.001
% (95%CI)	64 (58-69)	77 (70-83)	57 (45-68)	37 (26-49)	
No diagnosis	67 / 296	17 / 164	22 / 69	28 / 63	
% (95%CI)	23 (18-28)	10 (7-16)	32 (22-44)	44 (33-57)	
Asthma	16 / 296	8 / 164	4 / 69	4 / 63	
% (95%CI)	5 (3-9)	5 (2-9)	6 (2-14)	6 (2-15)	
Chronic bronchitis	14 /296	9 / 164	3 / 69	2 / 63	

% (95%CI)	5 (3-8)	5 (3-10)	4 (1-12)	3 (1-11)	
TB	4 / 296	3 / 164	0 / 69	1 / 63	
% (95%CI)	1 (0-3)	2 (0-5)	0 (0-0)	2 (0-8)	
COPD	2 / 296	0 / 164	0 / 69	2 / 63	
% (95%CI)	0.5 (0-2)	0 (0-0)	0 (0-0)	3 (1-11)	
Post-TB Lung Disease	2 / 296	1 / 164	1 / 69	0 / 63	
% (95%CI)	0.5 (0-2)	1 (0-3)	1.5 (0-8)	0 (0-0)	
Bronchiectasis	2 / 296	0 / 164	0 / 69	2 / 63	
% (95%CI)	0.5 (0-2)	0 (0-0)	0 (0-0)	3 (1-11)	
Congestive cardiac failure	1 / 296	0 / 164	0 / 69	1 / 63	
% (95%CI)	0.5 (0-2)	0 (0-0)	0 (0-0)	1.5 (0-8)	
Incurred catastrophic health expenditure*****	76 / 296	11 / 164	27 / 69	38 / 63	0.57
% (95%CI)	26 (21-31)	6.5 (3.8-11)	39 (28-51)	60 (48-71)	

Legend: p values compare participants by the healthcare level at which they sought care. For non-parametric continuous variables including age, participant income, household income, and household expenditure, 95% bootstrapped confidence intervals were generated and compared using Kruskal Wallis Test. For the remaining binary categorical variables, 95% Wilson confidence intervals of proportions were generated and compared using one way ANOVA test.

**Including higher college and University* ***In Kenyan Shillings* ****Participant income contributes >50% of household income*

*****Total monthly household expenditure including food, travel, rent, leisure, amenities, and health of other household members*

******The catastrophic health expenditure threshold is calculated as total health expenditure (total direct medical costs of participant + total direct non-medical costs of participant and carer/accompanying person) of >10% of total monthly household expenditure.*

Supplementary Table 1b: Sociodemographic and health characteristics of the study cohort and by five healthcare facilities visited (n=296)

	All participants (n=296)	Participants seeking care at Mitunguu health centre, Level 3 care (n=85)	Participants seeking care at Laare health centre, Level 3 care (n=79)	Participants seeking care at Mutuati Subcounty Hospital, Level 4 care (n=38)	Participants seeking care at Kanyakine Subcounty Hospital, Level 4 care (n=31)	Participants seeking care at Meru Teaching and Referral Hospital, Level 5 care (n=63)	p value
Sociodemographic factors							
Age in years							
Median	34	35	30	32	40	35	0.017
(95% CI)	(32-36)	(30-40)	(25-35)	(29-34)	(35-45)	(30-40)	
Age groups							
18-29 years	103 / 296	31 / 85	38 / 79	12 / 38	7 / 31	15 / 63	0.08
% (95%CI)	35 (30-40)	36 (27-47)	48 (37-59)	32 (19-47)	23 (11-40)	24 (15-36)	
30-44 years	117 / 296	31 / 85	26 / 79	18 / 38	12 / 31	30 / 63	
% (95%CI)	40 (34-45)	36 (27-47)	33 (23-44)	47 (32-63)	39 (24-56)	48 (36-60)	
45-59 years	60 / 296	17 / 85	13 / 79	5 / 38	10 / 31	15 / 63	
% (95%CI)	20 (16-25)	20 (13-30)	16 (10-26)	13 (6-27)	32 (19-50)	24 (15-36)	
60 years and above	16 / 296	6 / 85	2 / 79	3 / 38	2 / 31	3 / 63	
% (95%CI)	5 (0.3-8.5)	7 (3-15)	3 (1-9)	8 (3-21)	6 (2-21)	5 (2-13)	
Gender (%, 95% CI)							
Female	173 / 296	60 / 85	52 / 79	21 / 38	13 / 31	27 / 63	<0.001
% (95%CI)	58 (53-64)	71 (60-79)	66 (55-75)	55 (40-70)	42 (26-59)	43 (31-55)	
Male	123 / 296	25 / 85	27 / 79	17 / 38	18 / 31	36 / 63	
% (95%CI)	42 (36-47)	29 (21-40)	34 (25-45)	45 (30-69)	58 (41-74)	57 (45-69)	

Education level							
Up to primary school completion	163 / 296	49 / 85	47 / 79	27 / 38	13 / 31	27 / 63	0.093
% (95%CI)	55 (49-61)	58 (47-68)	59 (48-70)	71 (55-83)	42 (26-59)	43 (31-55)	
Up to secondary school completion	94 / 296	26 / 85	23 / 79	8 / 38	13 / 31	24 / 63	
% (95%CI)	32 (27-37)	31 (22-41)	29 (20-40)	21 (11-36)	42 (26-59)	38 (27-50)	
Above secondary school*	39 / 296	10 / 85	9 / 79	3 / 38	5 / 31	12 / 63	
% (95%CI)	13 (10-18)	12 (7-20)	11 (6-20)	8 (3-21)	16 (7-33)	19 (11-30)	
Mean monthly participant income**	4684	1748	2884	1600	4951	12630	<0.001
(95%CI)	(2633-6734)	(177-2318)	(1047-4721)	(373-2827)	(1888-8013)	(3443-21817)	
Mean monthly participant household income**	17482	12714	14754	11941	11382	33679	<0.001
(95%CI)	(14955-20009)	(10242-15187)	(10150-19358)	(9127-14755)	(7534-15229)	(25717-41641)	
Participant is not primary household income earner***	248 / 296	78 / 85	67 / 79	34 / 38	22 / 31	47 / 63	0.83
% (95%CI)	84 (79-88)	92 (74-96)	85 (75-91)	89 (76-96)	71 (53-84)	75 (63-84)	
Participant is primary household income earner	48 / 296	7 / 85	12 / 79	4 / 38	9 / 31	16 / 63	
% (95%CI)	16 (12-21)	8.0 (4.0-16)	15 (9.0-25)	11 (4.0-24)	29 (16-47)	25 (16-37)	
Mean monthly household expenditure****	12848	11439	13029	12512	9695	16277	<0.001

(95%CI)	(11923-13773)	(10353-12526)	(11022-15035)	(9718-15305)	(8144-11246)	(14141-18413)	
Asset-based poverty score							
First quintile (least poor)	60 / 296	18 / 85	17 / 79	8 / 38	6 / 31	11 / 63	0.12
% (95%CI)	20 (16-25)	21 (14-31)	22 (14-32)	21 (11-36)	19 (9.0-36)	17 (10-29)	
Second quintile	60 / 296	20 / 85	21 / 79	10 / 38	4 / 31	5 / 63	
% (95%CI)	20 (16-25)	24 (16-34)	27 (18-37)	26 (15-42)	13 (5.0-29)	8.0 (3.5-17)	
Third quintile	58 / 296	18 / 85	18 / 79	9 / 38	6 / 31	7 / 63	
% (95%CI)	20 (15-24)	21 (14-31)	23 (15-33)	24 (13-39)	19 (9.0-36)	11 (5.5-21)	
Fourth quintile	59 / 296	14 / 85	10 / 79	5 / 38	6 / 31	24 / 63	
% (95%CI)	20 (16-25)	16 (10-26)	13 (7.0-22)	13 (5.8-27)	19 (9.0-36)	38 (27-50)	
Fifth quintile (most poor)	59 / 296	13 / 85	13 / 79	6 / 38	9 / 31	16 / 63	
% (95%CI)	20 (16-25)	18 (11-27)	16 (10-26)	16 (7.5-30)	29 (16-47)	25 (16-37)	
NHIF membership/registration							
NHIF members/registered	84 / 296	26 / 85	17 / 79	4 / 38	11 / 31	26 / 63	0.086
% (95%CI)	28 (24-34)	31 (22-41)	22 (14-32)	11 (4-25)	35 (21-55)	41 (30-54)	
NHIF not members/registered	212 / 296	59 / 85	62 / 79	34 / 38	20 / 31	37 / 63	
% (95%CI)	72 (66-76)	69 (59-78)	78 (68-86)	89 (75-96)	65 (47-79)	59 (46-70)	
Health factors							
Accompanied while seeking care							
No	237 / 296	83 / 85	68 / 79	31 / 38	22 / 31	33 / 63	<0.001

% (95%CI)	80 (75-84)	98 (92-100)	86 (77-92)	82 (67-91)	71 (53-84)	52 (40-74)	
Yes	59 / 296	2 / 85	11 / 79	7 / 38	9 / 31	30 / 63	
% (95%CI)	20 (16-25)	2 (0-8)	14 (8-23)	18 (9-33)	29 (16-47)	48 (36-60)	
Final diagnosis							
Acute URTI or LRTI	188 / 296	67 / 85	59 / 79	25 / 38	14 / 31	23 / 63	<0.001
% (95%CI)	64 (58-69)	79 (69-86)	75 (64-83)	66 (50-79)	45 (29-62)	37 (26-49)	
No diagnosis	67 / 296	15 / 85	2 / 79	10 / 38	12 / 31	28 / 63	
% (95%CI)	23 (18-28)	18 (11-27)	3 (1-9)	26 (15-42)	39 (24-56)	44 (33-57)	
Asthma	16 / 296	3 / 85	5 / 79	2 / 38	2 / 31	4 / 63	
% (95%CI)	5 (3-9)	4 (1-10)	6 (3-14)	5 (1-17)	6 (2-21)	6 (2-15)	
Chronic bronchitis	14 /296	0 / 85	9 / 79	0 / 38	3 / 31	2 / 63	
% (95%CI)	5 (3-8)	0 (0-0)	11 (6-20)	0 (0-0)	10 (3-25)	3 (1-11)	
TB	4 / 296	0 / 85	3 / 79	0 / 38	0 / 31	1 / 63	
% (95%CI)	1 (0-3)	2 (0-5)	4 (1-11)	0 (0-0)	0 (0-0)	2 (0-8)	
COPD	2 / 296	0 / 85	0 / 79	0 / 38	0 / 31	2 / 63	
% (95%CI)	0.5 (0-2)	0 (0-0)	0 (0-0)	0 (0-0)	0 (0-0)	3 (1-11)	
Post-TB Lung Disease	2 / 296	0 / 85	1 / 79	1 / 38	0 / 31	0 / 63	
% (95%CI)	0.5 (0-2)	0 (0-0)	1 (0-7)	3 (0.5-13)	0 (0-0)	0 (0-0)	
Bronchiectasis	2 / 296	0 / 85	0 / 79	0 / 38	0 / 31	2 / 63	
% (95%CI)	0.5 (0-2)	0 (0-0)	0 (0-0)	0 (0-0)	0 (0-0)	3 (1-11)	
Congestive cardiac failure	1 / 296	0 / 85	0 / 79	0 / 38	0 / 31	1 / 63	
% (95%CI)	0.5 (0-2)	0 (0-0)	0 (0-0)	0 (0-0)	0 (0-0)	1.5 (0-8)	
Incurred catastrophic health expenditure*****	76 / 296	5 / 85	6 / 79	12 / 38	15 / 31	38 / 63	0.62
% (95%CI)	26 (21-31)	6.0 (2.5-13)	7.5 (3.5-16)	32 (19-47)	48 (32-65)	60 (48-71)	

Legend: p values compare participants by the specific healthcare facilities at which they sought care. For non-parametric continuous variables including age, participant income, household income, and household expenditure, 95% bootstrapped confidence intervals were generated and compared using Kruskal Wallis Test. For the remaining binary categorical variables, 95% Wilson confidence intervals of proportions were generated and compared using one way ANOVA test.

**Including higher college and University*

***In Kenyan Shillings*

****Participant income contributes >50% of household income*

*****Total monthly household expenditure including food, travel, rent, leisure, amenities, and health of other household members*

******The 10% catastrophic health expenditure threshold is calculated as total health expenditure (total direct medical costs of participant + total direct non-medical costs of participant and carer/accompanying person) of >10% of total monthly household expenditure.*

Supplementary Table 2a: Healthcare costs, lost income, and catastrophic health expenditure by three healthcare levels visited (n=296)

	All participants (n=296)	Participants seeking care at health centre (n=164)	Participants seeking care at Subcounty Hospital (n=69)	Participants seeking care at County Hospital (n=63)	p value
Direct medical costs of participant cohort					
Mean consultation costs (95%CI)	82 (74-89)	47 (43-51)	67 (60-73)	187 (175-200)	<0.001
Mean medicines costs (95%CI)	73 (56-91)	0 (0-0)	210 (164-257)	114 (71-157)	<0.001
Mean radiography costs (95%CI)	99 (68-130)	4.3 (0-13)	20 (0-49)	433 (324-542)	<0.001
Mean laboratory costs (95%CI)	133 (93-174)	54 (23-84)	114 (70-157)	362 (202-522)	<0.001
Mean other procedure costs (95%CI)	3 (0-6)	2 (1-4)	7 (0-22)	0 (0-0)	0.16
Direct non-medical costs of participant cohort					
Mean food costs (95%CI)	16 (10-22)	14 (7-20)	21 (8-34)	17 (4-29)	0.64
Mean travel costs (95%CI)	315 (272-357)	194 (164-223)	335 (280-390)	609 (468-750)	<0.001
Mean accommodation costs (95%CI)	0.4 (0-1)	1 (0-2)	0 (0-0)	0 (0-0)	0.67

Mean other costs (95%CI)	2.5 (1-4)	2 (0-3)	1 (0-3)	6.5 (1-12)	0.017
Direct non-medical costs of carer/accompanying person across participant cohort					
Participant not accompanied	237 / 296	151 / 164	53 / 69	33 / 63	<0.001
% (95%CI)	80 (75-84)	92 (87-95)	77 (66-85)	52 (40-64)	
Participant accompanied	59 / 296	13 / 164	16 / 69	30 / 63	
% (95%CI)	20 (16-25)	8 (5-13)	23 (15-34)	48 (36-60)	
Mean food costs (95%CI)	6 (2-10)	3 (0-6)	7 (0-17)	13 (0-25)	0.084
Mean travel costs (95%CI)	84 (55-113)	17 (6.5-27)	89 (41-137)	254 (151-357)	<0.001
Total direct medical costs of participant cohort					
Mean total direct medical costs (95%CI)	390 (324-457)	107 (68-146)	418 (354-483)	1097 (893-1300)	<0.001
Total direct non-medical costs of participant cohort (total direct non-medical costs of participant cohort and carer/accompanying person)					
Mean total direct non-medical costs (95%CI)	425 (361-489)	230 (195-264)	453 (360-547)	901 (692-1110)	<0.001
Total health expenditure of participant cohort (total direct medical costs of participant cohort + total direct non-medical costs of participant cohort and carer/accompanying person)					

Mean total direct costs<br>(95%CI)	815<br>(704-927)	337<br>(284-390)	872<br>(747-996)	1998<br>(1678-2318)	<0.001
Lost income and days of work of participant cohort					
Mean lost income<br>(95%CI)	247<br>(153-341)	105<br>(45-165)	252<br>(51-453)	610<br>(271-949)	<0.001
Mean days of work lost<br>(95%CI)	1.7<br>(1.5-1.9)	1.1<br>(1.0-1.3)	2.3<br>(1.7-2.9)	2.4<br>(1.8-3.0)	<0.001
Total expenditure of participant cohort (total health expenditure of participant cohort + lost income of participant cohort)					
Mean total costs<br>(95%CI)	1062<br>(896-1228)	442<br>(360-524)	1124<br>(907-1341)	2608<br>(2087-3129)	<0.001

Legend: All costs are given in Kenyan Shillings. p values compare participants depending on the healthcare level at which they sought care (i.e. health centre, subcounty hospital, hospital). For non-parametric continuous variables including direct medical costs of participant (consultation, medicines, radiography, laboratory, other procedure), direct non-medical costs of participant (food, travel, accommodation, other including mobile telecommunications airtime), direct non-medical costs of carer/accompanying person (food, travel), total direct medical costs of participant, total direct non-medical costs of participant and carer/accompanying person, days of work lost, lost income of participant, and total expenditure, 95% bootstrapped confidence intervals were generated and compared using the Kruskal Wallis test. For the remaining binary categorical variable, participant accompanied, 95% Wilson confidence intervals of proportions were generated and compared using one way ANOVA.

Supplementary Table 2b: Healthcare costs, lost income, and catastrophic health expenditure by five healthcare facilities visited (n=296)

	All participants (n=296)	Participants seeking care at Mitunguu health centre, Level 3 care (n=85)	Participants seeking care at Laare health centre, Level 3 care (n=79)	Participants seeking care at Mutuati Subcounty Hospital, Level 4 care (n=38)	Participants seeking care at Kanyakine Subcounty Hospital, Level 4 care (n=31)	Participants seeking care at Meru Teaching and Referral Hospital, Level 5 care (n=63)	p value
Direct medical costs of participant cohort							
Mean consultation costs	82	50	44	46	92	187	<0.001
(95%CI)	(74-89)	(44-57)	(39-48)	(42-50)	(84-100)	(175-200)	
Mean medicines costs	73	0	0	280	125	114	<0.001
(95%CI)	(56-91)	(0-0)	(0-0)	(218-342)	(71-180)	(71-157)	
Mean radiography costs	99	8	0	0	45	433	<0.001
(95%CI)	(68-130)	(0-24)	(0-0)	(0-0)	(0-105)	(324-542)	
Mean laboratory costs	133	25	85	73	163	362	<0.001
(95%CI)	(93-174)	(0-67)	(42-128)	(36-111)	(81-245)	(202-522)	
Mean other procedure costs	3	0	4	0	16	0	0.0025
(95%CI)	(0-6)	(0-0)	(1-8)	(0-0)	(0-47)	(0-0)	

Direct non-medical costs of participant cohort							
Mean food costs (95%CI)	16 (10-22)	7 (1-13)	21 (9-33)	14 (0-29)	29 (6-52)	17 (4-29)	0.045
Mean travel costs (95%CI)	315 (272-357)	173 (132-215)	216 (178-254)	265 (203-326)	421 (330-511)	609 (468-750)	<0.001
Mean accommodation costs (95%CI)	0.4 (0-1)	1 (0-2)	0 (0-0)	1 (0-4)	0 (0-0)	0 (0-0)	0.6
Mean other costs (95%CI)	2.5 (1-4)	2.5 (0-5)	1 (0-2)	0 (0-0)	3 (0-7)	6.5 (1-12)	0.02
Direct non-medical costs of carer/accompanying person across participant cohort							
Participant not accompanied	237 / 296	83 / 85	68 / 79	31 / 38	22 / 31	33 / 63	<0.001
% (95%CI)	80 (75-84)	98 (92-100)	86 (77-92)	82 (67-91)	71 (53-84)	52 (40-64)	
Participant accompanied	59 / 296	2 / 85	11 / 79	7 / 38	9 / 31	30 / 63	
% (95%CI)	20 (16-25)	2 (0-8)	14 (8-23)	18 (9-33)	29 (16-47)	48 (36-60)	
Mean food costs (95%CI)	6 (2-10)	1 (0-2)	6 (0-13)	5 (0-15)	10 (0-28)	13 (0-25)	0.17
Mean travel costs (95%CI)	84 (55-113)	0 (0-0)	35 (15-56)	42 (8.5-76)	146 (45-248)	254 (151-357)	<0.001

Total direct medical costs of participant cohort							
Mean total direct medical costs	390	83	133	399	441	1097	<0.001
(95%CI)	(324-456)	(21-146)	(89-176)	(323-476)	(334-550)	(893-1300)	
Total direct non-medical costs of participant cohort (total direct non-medical costs of participant cohort and carer/accompanying person)							
Mean total direct non-medical costs	425	183	280	327	609	901	<0.001
(95%CI)	(361-489)	(138-228)	(230-331)	(235-418)	(441-777)	(692-1110)	
Total health expenditure of participant cohort (total direct medical costs of participant cohort + total direct non-medical costs of participant cohort and carer/accompanying person)							
Mean total direct costs	815	267	413	726	1051	1998	<0.001
(95%CI)	(704-927)	(187-346)	(347-479)	(602-850)	(825-1276)	(1678-2318)	

Lost income and days of work of participant cohort							
Mean lost income (95%CI)	247 (153-341)	49 (35-63)	165 (42-288)	216 (0-543)	295 (145-446)	610 (271-949)	<0.001
Mean days of work lost (95%CI)	1.7 (1.5-1.9)	1 (0.87-1.1)	1.4 (1.1-1.7)	1.7 (1.2-2.1)	3.1 (2.0-4.2)	2.4 (1.8-3.0)	<0.001
Total expenditure of participant cohort (total health expenditure of participant cohort + lost income of participant cohort)							
Mean total costs (95%CI)	1062 (8961228)	316 (234-397)	578 (432-725)	942 (628-1257)	1346 (1107-1586)	2608 (2087-3129)	<0.001

Legend: All costs are given in Kenyan Shillings. p values compare participants depending on the healthcare facility at which they sought care. For non-parametric continuous variables including direct medical costs of participant (consultation, medicines, radiography, laboratory, other procedure), direct non-medical costs of participant (food, travel, accommodation, other including mobile telecommunications airtime), direct non-medical costs of carer/accompanying person (food, travel), total direct medical costs of participant, total direct non-medical costs of participant and carer/accompanying person, days of work lost, lost income of participant, and total expenditure, 95% bootstrapped confidence intervals were generated and compared using the Kruskal Wallis test. For the remaining binary categorical variable, participant accompanied, 95% Wilson confidence intervals of proportions were generated and compared using one way ANOVA.

Supplementary Table 3a: Coping strategies and NHIF coverage and use by healthcare level visited (n=296)

	All participants (n=296)	Participants seeking care at health centre (n=164)	Participants seeking care at Subcounty Hospital (n=69)	Participants seeking care at County Hospital (n=63)	p value
Coping strategies (%)					
Use savings	173 / 296	88 / 164	42 / 69	43 / 63	0.28
% (95%CI)	58 (53-64)	54 (46-61)	61 (49-72)	68 (56-78)	
Borrow	91 / 296	51 / 164	19 / 69	21 / 63	0.67
% (95%CI)	31 (26-36)	31 (25-39)	28 (18-39)	33 (23-45)	
Money from other sources	22 / 296	12 / 164	5 / 69	5 / 63	0.81
% (95%CI)	7 (5-11)	7 (4-12)	7 (3-16)	8 (3-17)	
Additional work	20 / 296	9 / 164	4 / 69	7 / 63	0.42
% (95%CI)	7 (4.5-10)	5 (3-10)	6 (2-14)	11 (5-21)	
Sell property	11 / 296	3 / 164	2 / 69	5 / 63	0.028
% (95%CI)	4 (2-7)	2 (1-5)	4 (1-12)	8 (3-17)	
Loans	7 / 296	2 / 164	3 / 69	2 / 63	0.97
% (95%CI)	2 (1-5)	1 (0-4)	4 (1-12)	3 (1-11)	
Default on rent	1 / 296	0 / 164	0 / 69	1 / 63	0.096
% (95%CI)	0.5 (0-2)	0 (0-0)	0 (0-0)	2 (0-8)	
Number of coping strategies used (%)					
None	14 / 296	13 / 64	1 / 69	0 / 63	0.005
% (95%CI)	4.5 (3-8)	8 (5-13)	1 (0-8)	0 (0-0)	
1	245 / 296	138 / 164	60 / 69	47 / 63	0.16
% (95%CI)	83 (78-87)	84 (78-89)	87 (77-93)	75 (63-84)	
2 or more	37 / 296	13 / 164	8 / 69	16 / 63	<0.001
% (95%CI)	12.5 (9-17)	8 (5-13)	12 (6-21)	25 (16-37)	

National Hospital Insurance Fund (NHIF) member (%)					
Yes	84 / 296	43 / 164	15 / 69	26 (41)	0.063
% (95%CI)	28 (24-34)	26 (20-33)	22 (14-33)	41 (30-54)	
No	212 / 296	121 / 164	54 / 69	37 / 63	
% (95%CI)	72 (66-76)	74 (67-80)	78 (67-86)	59 (46-70)	
If yes NHIF, was NHIF used to pay for care? (n=84)					
Yes, NHIF was used to pay for care	11 / 84	0 / 43	1 / 15	10 / 26	<0.001
% (95%CI)	13 (7-12)	0 (0-0)	7 (1-30)	38 (22-56)	
No, NHIF was not used to pay for care	73 / 84	43 / 43	14 / 15	16 / 26	
% (95%CI)	87 (78-93)	100 (92-100)	93 (70-99)	62 (43-78)	
If no, main reason for not using NHIF to pay for care (n=73)					
Did not ask or was not asked about NHIF	30 / 73	22 / 43	5 / 14	3 / 16	0.17
% (95%CI)	41 (31-53)	51 (37-65)	36 (16-61)	19 (6.5-43)	
Not up-to-date with NHIF premium payments	22 / 73	12 / 43	5 / 14	5 / 16	
% (95%CI)	30 (21-41)	28 (17-43)	36 (16-61)	31 (14-56)	
Facility not signed up to NHIF and/or NHIF card not taken	16 / 73	8 / 43	1 / 14	7 / 16	
% (95%CI)	22 (14-33)	19 (10-33)	7 (1-31)	44 (23-67)	
Forgot card or other required identification	3 / 73	1 / 43	1 / 15	1 / 16	
% (95%CI)	4 (1-11)	2 (0-12)	7 (1-31)	6 (1-28)	
Did not know services covered by NHIF	2 / 73	0 / 43	2 / 14	0 / 16	
% (95%CI)	3 (0.5-9)	0 (0-0)	14 (4-40)	0 (0-0)	
If yes, monetary value covered by NHIF when used to pay for care (n=11)					
Mean Kenya Shillings covered by NHIF	1278	0	140*	1392	0.11

(95%CI)	(506-2050)	(0)	(140-140)	(432-2352)	
If no NHIF, main reason for not having NHIF (n=212)					
NHIF is unaffordable	92 / 212	49 / 121	20 / 54	23 / 37	0.08
% (95%CI)	43 (37-50)	40 (32-49)	37 (25-50)	62 (46-76)	
Unaware of NHIF	56 / 212	36 / 121	17 / 54	3 / 37	
% (95%CI)	26 (21-33)	30 (22-38)	32 (21-45)	8 (3-21)	
Lack of information	44 / 212	25 / 121	11 / 54	8 / 37	
% (95%CI)	21 (16-27)	21 (14-29)	20 (12-33)	22 (11-37)	
Services/package offered is inadequate	2 / 212	1 / 121	0 / 54	1 / 37	
% (95%CI)	1 (0-3)	1 (0-5)	0 (0-0)	3 (0-14)	
Choice of facility is limited	0 / 212	0 / 121	0 / 54	0 / 37	
% (95%CI)	0 (0-0)	0 (0-0)	0 (0-0)	0 (0-0)	
Other**	18 / 296	10 / 121	6 / 54	2 / 37	
% (95%CI)	8.5 (5-13)	8 (5-15)	11 (5-22)	5 (1-18)	

*Legend: p values compare participants depending on the healthcare level at which they sought care (i.e. health centre, subcounty hospital, hospital). For the single non-parametric continuous variable of Kenya shillings covered by NHIF, a 95% bootstrapped confidence interval was generated and compared using Kruskal Wallis test. All other variables in the table were binary categorical variables for which 95% Wilson confidence intervals of proportions were generated and compared using one way ANOVA test. * Only a single participant ** Still plan to enrol (n=9), issues with required documents (n=2), issues with identity card (n=3), stopped submitting NHIF annual premiums (n=2), no specific reason (n=2)*

Supplementary Table 3b: Coping strategies and NHIF coverage and use by five healthcare facilities visited (n=296)

	All participants (n=296)	Participants seeking care at Mitunguu health centre, Level 3 care (n=85)	Participants seeking care at Laare health centre, Level 3 care (n=79)	Participants seeking care at Mutuati Subcounty Hospital, Level 4 care (n=38)	Participants seeking care at Kanyakine Subcounty Hospital, Level 4 care (n=31)	Participants seeking care at Meru Teaching and Referral Hospital, Level 5 care (n=63)	p value
Coping strategies (%)							
Use savings	173 / 296	45 / 85	36 / 79	25 / 38	17 / 31	43 / 63	0.06
% (95%CI)	58 (53-64)	53 (42-63)	54 (43-65)	66 (50-79)	55 (38-71)	68 (56-78)	
Borrow	91 / 296	28 / 85	23 / 79	9 / 38	10 / 31	21 / 63	0.91
% (95%CI)	31 (26-36)	33 (24-43)	29 (20-40)	24 (13-39)	32 (19-50)	33 (23-45)	
Money from other sources	22 / 296	5 / 85	7 / 79	2 / 38	3 / 31	5 / 63	0.67
% (95%CI)	7 (5-11)	6 (3-13)	9 (4-17)	5 (1-17)	10 (3-25)	8 (3-17)	
Additional work	20 / 296	7 / 85	2 / 79	1 / 37	3 / 31	7 / 63	0.27
% (95%CI)	7 (4.5-10)	8 (4-16)	3 (1-9)	3 (0-13)	10 (3-25)	11 (5-21)	
Sell property	11 / 296	1 / 85	2 / 79	2 / 38	2 / 31	5 / 63	0.02
% (95%CI)	4 (2-7)	1 (0-6)	3 (1-9)	3 (0-13)	6 (2-21)	8 (3-17)	
Loans	7 / 296	0 / 85	2 / 79	0 / 38	3 / 31	2 / 63	0.07
% (95%CI)	2 (1-5)	0 (0-0)	3 (1-9)	0 (0-0)	10 (3-25)	3 (1-11)	
Default on rent	1 / 296	0 / 85	0 / 79	0 / 38	0 / 31	1 / 63	0.13
% (95%CI)	0.5 (0-2)	(0-0)	(0-0)	(0-0)	0 (0-0)	2 (0-8)	
Number of coping strategies used (%)							
None	14 / 296	6 / 85	7 / 79	1 / 38	0 / 31	0 / 63	0.01
% (95%CI)	4.5 (3-8)	7 (3-15)	9 (4-17)	3 (0-13)	0 (0-0)	0 (0-0)	
1	245 / 296	73 / 85	65 / 79	36 / 38	24 / 31	47 / 63	0.09
% (95%CI)	83 (78-87)	86 (77-92)	83 (72-89)	95 (83-99)	77 (60-89)	75 (63-84)	

2 or more	37 / 296	6 / 85	7 / 79	1 / 38	7 / 31	16 / 63	<0.001
% (95%CI)	12.5 (9-17)	7 (3-15)	9 (4-17)	3 (0-13)	23 (11-40)	25 (16-37)	
National Hospital Insurance Fund (NHIF) member (%)							
Yes	84 / 296	26 / 85	17 / 79	4 / 38	11 / 31	26 (41)	0.09
% (95%CI)	28 (24-34)	31 (22-41)	22 (14-32)	11 (4-24)	35 (21-53)	41 (30-54)	
No	212 / 296	59 / 85	62 / 79	34 / 38	20 / 31	37 / 63	
% (95%CI)	72 (66-76)	69 (59-78)	78 (68-86)	89 (76-96)	65 (47-79)	59 (46-70)	
If yes NHIF, was NHIF used to pay for care? (n=84)							
Yes, NHIF was used to pay for care	11 / 84	0 / 26	0 / 17	1 / 4	10 / 26	10 / 26	<0.001
% (95%CI)	13 (7-12)	0 (0-0)	0 (0-0)	25 (1-30)	38 (22-56)	38 (22-56)	
No, NHIF was not used to pay for care	73 / 84	26 / 26	17 / 17	3 / 4	16 / 26	16 / 26	
% (95%CI)	87 (78-93)	100 (78-93)	100 (92-100)	75 (70-99)	62 (43-78)	62 (43-78)	
If no, main reason for not using NHIF to pay for care (n=73)							
Did not ask or was not asked about NHIF	30 / 73	12 / 26	10 / 17	0 / 3	5 / 11	3 / 16	0.26
% (95%CI)	41 (31-53)	46 (29-65)	59 (36-78)	0 (0-0)	45 (21-72)	19 (6.5-43)	
Not up-to-date with NHIF premium payments	22 / 73	9 / 26	3 / 17	2 / 3	3 / 11	5 / 16	
% (95%CI)	30 (21-41)	35 (19-54)	18 (6-41)	67 (21-94)	27 (10-57)	31 (14-56)	
Facility not signed up to NHIF and/or NHIF card not taken	16 / 73	4 / 26	4 / 17	0 / 3	1 / 11	7 / 16	
% (95%CI)	22 (14-33)	15 (6-34)	24 (10-47)	0 (0-0)	9 (2-38)	44 (23-67)	
Forgot card or other required identification	3 / 73	1 / 26	0 / 17	1 / 3	0 / 11	1 / 16	
% (95%CI)	4 (1-11)	4 (1-19)	0 (0-0)	33 (6-79)	0 (0-0)	6 (1-28)	
Did not know services covered by NHIF	2 / 73	0 / 26	0 / 17	0 / 3	2 / 11	0 / 16	
% (95%CI)	3 (0.5-9)	0 (0-0)	0 (0-0)	0 (0-0)	18 (5-48)	0 (0-0)	

If yes, monetary value covered by NHIF when used to pay for care (n=11)							
Mean Kenya Shillings covered by NHIF	1278	0	0	140*	0	1392.00	0.11
(95%CI)	(506-2050)	(0-0)	(0-0)	(140-140)	(0-0)	(432-2352)	
If no NHIF, main reason for not having NHIF (n=212)							
NHIF is unaffordable	92 / 212	28 / 59	21 / 62	10 / 34	10 / 20	23 / 37	0.21
% (95%CI)	43 (37-50)	47 (35-60)	34 (23-46)	29 (17-46)	50 (30-70)	62 (46-76)	
Unaware of NHIF	56 / 212	19 / 59	17 / 62	12 / 34	5 / 20	3 / 37	
% (95%CI)	26 (21-33)	32 (22-45)	27 (18-40)	35 (21-52)	25 (11-47)	8 (3-21)	
Lack of information	44 / 212	9 / 59	16 / 62	10 / 34	1 / 20	8 / 37	
% (95%CI)	21 (16-27)	15 (8-27)	26 (17-38)	29 (17-46)	5 (1-24)	22 (11-37)	
Services/package offered is inadequate	2 / 212	1 / 59	0 / 62	0 / 34	0 / 20	1 / 37	
% (95%CI)	1 (0-3)	2 (0-9)	0 (0-0)	0 (0-0)	0 (0-0)	3 (0-14)	
Choice of facility is limited	0 / 212	0 / 59	0 / 62	0 / 34	0 / 20	0 / 37	
% (95%CI)	0 (0-0)	0 (0-0)	0 (0-0)	0 (0-0)	0 (0-0)	0 (0-0)	
Other**	18 / 296	2 / 59	8 / 62	2 / 34	4 / 20	2 / 37	
% (95%CI)	8.5 (5-13)	3 (1-12)	13 (7-23)	6 (2-19)	20 (8-42)	5 (1-18)	

*Legend: p values compare the participants depending on the healthcare facility at which they sought care. For the single non-parametric continuous variable of Kenya shillings covered by NHIF, a 95% bootstrapped confidence interval was generated and compared using Kruskal Wallis test. All other variables in the table were binary categorical variables for which 95% Wilson confidence intervals of proportions were generated and compared using one way ANOVA test. * Only a single participant ** Still plan to enrol (n=9), issues with required documents (n=2), issues with identity card (n=3), stopped submitting NHIF annual premiums (n=2), and no specific reason (n=2).*

Supplementary File 9: A priori sensitivity univariable and multivariable logistic regression analyses

Sensitivity multivariable logistic regression analyses were performed using both three healthcare levels and five healthcare facility levels (instead of only using three healthcare levels shown in Table 4's multivariable logistic regression analysis in the main manuscript) at which participants sought care against the 10% CHE threshold with i) the addition of lost income to the numerator as per WHO TB Patient Cost Surveys[4] and ii) the inclusion of *only* direct medical costs of participants and *not* non-medical costs of participants and any accompanying person in the numerator. Each regression analysis is accompanied by the Stata output of interaction testing using the mfpigen function.

Supplementary Table 4: Univariable and multivariable logistic regression of health and social factors associated with catastrophic health expenditure including by five healthcare facilities

This supplementary table is equivalent to Table 4 in the main manuscript and uses the same 10% CHE threshold, which include both direct medical and direct non-medical costs in the numerator to calculate CHE but uses a five-factor term for healthcare facilities visited rather than a three-factor term for healthcare level visited.

	**Catastrophic health expenditure**			
	**Unadjusted univariable odds ratio (95%CI)**	**p value**	**Adjusted multivariable odds ratio (95%CI)**	**p value**
Age groups				
18-29 years	REF		REF	
30-44 years	**3.7 (2.9-4.6)**	**<0.001**	**2.5 (1.0-6.2)**	**0.044**
45-59 years	**3.4 (1.7-6.7)**	**0.001**	2.0 (0.69-5.7)	0.21
60 years and above	2.3 (0.69-7.5)	0.18	1.0 (0.19-5.4)	0.99
Gender				
Male	REF		REF	
Female	0.69 (0.35-2.0)	0.71	**2.0 (0.95-4.0)**	**0.07**
Education level				
Up to primary school completion	REF		REF	
Up to secondary school completion	**1.3 (1.2-1.5)**	**<0.001**	0.89 (0.38-2.1)	0.78
Above secondary school	**1.7 (0.99-2.8)**	**0.053**	1.4 (0.50-4.2)	0.50
Smoker				
Never smoker	REF		__	

Current or past smoker	0.94 (0.23-2.8)	0.93		
Primary income earner				
No	REF		—	
Yes	1.1 (0.69-1.7)	0.7		
Accompanied during healthcare seeking and visits				
No	REF		REF	
Yes	**6.5 (4.1-10)**	**<0.001**	**3.0 (1.4-6.7)**	**0.006**
Asset-based poverty score				
First quintile (least poor)	REF		REF	
Second quintile	1.4 (0.76-2.4)	0.30	2.1 (0.70-6.1)	0.099
Third quintile	0.99 (0.77-1.3)	0.91	0.73 (0.22-2.4)	0.60
Fourth quintile	1.2 (0.96-1.4)	0.13	0.49 (0.16-1.5)	0.20
Fifth quintile (poorest)	**2.2 (1.1-4.7)**	**0.035**	2.0 (0.71-5.6)	0.16
NHIF member				
Yes	REF		REF	
No	1.3 (0.94-1.8)	0.11	1.7 (0.72-3.8)	0.24
Number of coping strategies used				
None or single coping strategy used	REF		—	
Multiple coping strategies used	**3.0 (1.4-6.4)**	**0.004**		
Diagnosis				
Any diagnosis	REF		—	
No diagnosis	1.6 (0.48-5.1)	0.47		

Healthcare facility and level at which seeking care				
Mitungu Health Centre (Level 3)	REF		REF	
Laare Health Centre (Level 3)	**1.4 (1.3-1.6)**	**<0.001**	1.1 (0.29-3.9)	0.91
Mutuati Subcounty Hospital (Level 4)	**8.1 (6.5-10)**	**<0.001**	**6.8 (2.0-23)**	**0.002**
Kanyakine Subcounty Hospital (Level 4)	**18 (15-23)**	**<0.001**	**17 (4.7-63)**	**<0.001**
Meru Teaching and Referral Hospital (Level 5)	**30 (23-41)**	**<0.001**	**29 (8-99)**	**<0.001**

Legend: The catastrophic health expenditure threshold is calculated as total health expenditure (total direct medical costs of participant + total direct non-medical costs of participant and carer/accompanying person) of >10% of total monthly household expenditure. Interaction testing of the model of factors associated with 10% catastrophic health expenditure demonstrated an interaction between coping strategies, healthcare facility used, and educational level and coping strategies was dropped from the multivariable model (interaction testing shown below).

Interaction testing, including by five health facilities and coping strategies, is shown below:

variable 1	function 1	variable 2	function 2	dev. diff.	d.f.	P	Sel
i.agegroups	Factor	i.genderfe~e	Factor	2.1592	2	0.3397	6
	Factor	i.educatio~l	Factor	4.1734	6	0.6532	6
	Factor	i.finalpov~e	Factor	8.9061	9	0.4460	6
	Factor	i.patient_~d	Factor	4.1096	3	0.2499	6
	Factor	i.insuranc~r	Factor	3.1284	3	0.3722	6
	Factor	i.multiple~g	Factor	3.8989	2	0.1423	6
	Factor	i.facility~r	Factor	17.2622	10	0.0688	6
i.genderfe~e	Factor	i.educatio~l	Factor	0.6617	2	0.7183	6
	Factor	i.finalpov~e	Factor	6.7731	4	0.1484	6
	Factor	i.patient_~d	Factor	0.3508	1	0.5536	6
	Factor	i.insuranc~r	Factor	0.5167	1	0.4723	6
	Factor	i.multiple~g	Factor	3.7985	1	0.0513	6
	Factor	i.facility~r	Factor	0.7044	4	0.9508	6
i.educatio~l	Factor	i.finalpov~e	Factor	5.1704	8	0.7392	6
	Factor	i.patient_~d	Factor	1.7238	2	0.4224	6
	Factor	i.insuranc~r	Factor	3.1889	2	0.2030	6
	Factor	i.multiple~g	Factor	7.1591	2	0.0279	6
	Factor	i.facility~r	Factor	11.4459	5	0.0432	6
i.finalpov~e	Factor	i.patient_~d	Factor	4.8976	4	0.2980	6
	Factor	i.insuranc~r	Factor	3.0814	4	0.5443	6
	Factor	i.multiple~g	Factor	1.8143	4	0.7699	6
	Factor	i.facility~r	Factor	13.3581	13	0.4205	6
i.patient_~d	Factor	i.insuranc~r	Factor	1.9268	1	0.1651	6
	Factor	i.multiple~g	Factor	0.0002	1	0.9875	6
	Factor	i.facility~r	Factor	4.1578	3	0.2449	6
i.insuranc~r	Factor	i.multiple~g	Factor	0.0748	1	0.7844	6
	Factor	i.facility~r	Factor	6.9905	2	0.0303	6
i.multiple~g	Factor	i.facility~r	Factor	6.3689	1	0.0116	6

Legend: facility~r = five-factor healthcare facility term, agegroups = age groups, genderfemale = gender, educatio~l = education level, finalpov~e = poverty quintile, patient_~d = patient accompanied by carer, insuranc~r = insurance coverage, multiple~g = coping strategies

Following the above, interaction testing was repeated, by five health facilities but excluding coping strategies, and is shown below:

variable 1	function 1	variable 2	function 2	dev. diff.	d.f.	P	Sel
i.agegroups	Factor	i.genderfe~e	Factor	2.0504	2	0.3587	5
	Factor	i.educatio~l	Factor	4.4142	6	0.6208	5
	Factor	i.finalpov~e	Factor	8.6864	9	0.4667	5
	Factor	i.patient_~d	Factor	3.9893	3	0.2626	5
	Factor	i.insuranc~r	Factor	3.1039	3	0.3759	5
	Factor	i.facility~r	Factor	17.1531	10	0.0710	5
i.genderfe~e	Factor	i.educatio~l	Factor	0.9094	2	0.6346	5
	Factor	i.finalpov~e	Factor	6.7632	4	0.1489	5
	Factor	i.patient_~d	Factor	0.2431	1	0.6220	5
	Factor	i.insuranc~r	Factor	0.4973	1	0.4807	5
	Factor	i.facility~r	Factor	0.8172	4	0.9361	5
i.educatio~l	Factor	i.finalpov~e	Factor	5.0256	8	0.7548	5
	Factor	i.patient_~d	Factor	1.6963	2	0.4282	5
	Factor	i.insuranc~r	Factor	3.0950	2	0.2128	5
	Factor	i.facility~r	Factor	12.0096	5	0.0347	5
i.finalpov~e	Factor	i.patient_~d	Factor	4.7474	4	0.3142	5
	Factor	i.insuranc~r	Factor	2.3973	4	0.6631	5
	Factor	i.facility~r	Factor	13.6398	13	0.3997	5
i.patient_~d	Factor	i.insuranc~r	Factor	1.6887	1	0.1938	5
	Factor	i.facility~r	Factor	3.9719	3	0.2645	5
i.insuranc~r	Factor	i.facility~r	Factor	6.8558	2	0.0325	5

Legend: facility~r = five-factor healthcare facility term, agegroups = age groups, genderfemale = gender, educatio~l = education level, finalpov~e = poverty quintile, patient_~d = patient accompanied by carer, insuranc~r = insurance coverage

Supplementary Table 5: "Medical costs only" sensitivity analysis showing univariable and multivariable logistic regression of health and social factors associated with catastrophic health expenditure by three health system levels

This supplementary table is equivalent to Table 4 in the main manuscript but uses a different CHE threshold, which included only direct medical costs (and excluded non-medical costs) in the numerator to calculate CHE.

	**Catastrophic health expenditure at 10% of monthly total household expenditure threshold using medical costs only**				
	**n/N (%)**	**Unadjusted univariable odds ratio (95%CI)**	**p value**	**Adjusted multivariable odds ratio (95%CI)**	**p value**
Proportion with catastrophic health expenditure (%)	32/296 (11)	_		_	
Age groups					
18-29 years	6/103 (6.0)	REF		REF	
30-44 years	16/117 (14)	**2.5 (1.6-4.0)**	**<0.001**	1.0 (0.29-3.8)	0.95
45-59 years	9/60 (15)	**2.8 (1.4-5.8)**	**0.004**	1.2 (0.17-7.8)	0.89
60 years and above	1/16 (6.0)	**1.1 (0.086-14)**	**0.94**	0.52 (0.036-7.5)	0.63
Gender					
Male	13/123 (11)	REF		REF	
Female	19/173 (11)	1.1 (0.21-5.5)	0.93	2.5 (0.59-11)	0.21
Education level					
Up to primary school completion	20/163 (12)	REF		REF	
Up to secondary school completion	10/94 (11)	1.2 (0.86-1.6)	0.35	0.75 (0.49-1.1)	0.18
Above secondary school	2/39 (5.0)	0.51 (0.14-1.9)	0.31	**0.30 (0.11-0.82)**	**0.019**

Smoker					
Never smoker	28/253 (11)	REF		_	
Current or past smoker	4/43 (9.0)	0.71 (0.17-2.9)	0.63		
Primary income earner					
No	26/248 (10)	REF		_	
Yes	6/48 (13)	1.4 (0.56-3.7)	0.46		
Accompanied during healthcare seeking and visits					
No	18/237 (7.5)	REF		REF	
Yes	14/59 (24)	**3.9 (2.2-7.2)**	**<0.001**	1.7 (0.92-3.0)	0.092
Asset-based poverty score					
First quintile (least poor)	3/60 (5.0)	REF		REF	
Second quintile	6/60 (10)	**2.2 (1.2-4.1)**	**0.015**	**2.4 (1.2-4.6)**	**<0.001**
Third quintile	3/58 (5.0)	1.2 (0.38-4.0)	0.74	1.2 (0.75-1.8)	0.5
Fourth quintile	5/59 (8.5)	1.9 (0.90-4.2)	0.091	0.28 (0.03-3.1)	0.3
Fifth quintile (poorest)	15/59 (25)	**6.2 (2.8-14)**	**<0.001**	1.7 (0.70-4.0)	0.25
NHIF member					
Yes	9/84 (11)	REF		REF	
No	23/212 (11)	**1.0 (1.01-1.04)**	**0.001**	1.4 (0.66-2.8)	0.40
Number of coping strategies used					
None or single coping strategy used	22/259 (8.5)	REF		__	
Multiple coping strategies used	10/37 (27)	**3.7 (2.7-5.0)**	**<0.001**		
Diagnosis					
Any diagnosis	22/229 (9.5)	REF		__	
No diagnosis	10/67 (15)	1.6 (0.40-6.9)	0.49		
Health system level at which seeking care					

Health centre	4/164 (2.5)	REF		REF	
Sub-county hospital	9/69 (13)	**7.0 (5.3-9.3)**	**<0.001**	**6.7 (5.3-8.6)**	**<0.001**
County hospital	19/63 (30)	**25 (14-45)**	**<0.001**	**29 (21-41)**	**<0.001**

Legend: In this sensitivity analysis, the catastrophic health expenditure threshold is calculated as total direct medical costs of participant of >10% of total monthly household expenditure. In contrast to the main analysis in Table 4 of the manuscript, the direct non-medical costs of the participant and the person or carer accompanying them such as food, travel, and accommodation, are not included in the calculation. Interaction testing of the model of factors associated with catastrophic health expenditure demonstrated an interaction between coping strategies and poverty level and coping strategies was dropped from the multivariable model. The interaction testing for this model is not shown in this supplementary file.

Supplementary Table 6: "Medical costs only" sensitivity analysis showing univariable and multivariable logistic regression of health and social factors associated with catastrophic health expenditure by five healthcare facilities

	Catastrophic health expenditure at 10% of monthly total household expenditure threshold using medical costs only				
	**n/N (%)**	**Unadjusted univariable odds ratio (95%CI)**	**p value**	**Adjusted multivariable odds ratio (95%CI)**	**p value**
Proportion with catastrophic health expenditure (%)	32/296 (11)	_		_	
Age groups					
18-29 years	6/103 (6.0)	REF		REF	
30-44 years	16/117 (14)	**2.5 (1.6-4.0)**	**<0.001**	1.1 (0.25-4.5)	0.95
45-59 years	9/60 (15)	**2.8 (1.4-5.8)**	**0.004**	1.0 (0.19-5.3)	0.99
60 years and above	1/16 (6.0)	**1.1 (0.086-14)**	**0.94**	0.44 (0.011-17)	0.66
Gender					
Male	13/123 (11)	REF		REF	
Female	19/173 (11)	1.1 (0.21-5.5)	0.93	2.8 (0.67-12)	0.16
Education level					
Up to primary school completion	20/163 (12)	REF		REF	
Up to secondary school completion	10/94 (11)	1.2 (0.86-1.6)	0.35	**0.63 (0.42-0.94)**	**0.023**
Above secondary school	2/39 (5.0)	0.51 (0.14-1.9)	0.31	**0.25 (0.087-0.74)**	**0.012**
Smoker					
Never smoker	28/253 (11)	REF		_	
Current or past smoker	4/43 (9.0)	0.71 (0.17-2.9)	0.63		

Primary income earner					
No	26/248 (10)	REF		_	
Yes	6/48 (13)	1.4 (0.56-3.7)	0.46		
Accompanied during healthcare seeking and visits					
No	18/237 (7.5)	REF		REF	
Yes	14/59 (24)	**3.9 (2.2-7.2)**	**<0.001**	1.5 (0.81-2.9)	0.19
Asset-based poverty score					
First quintile (least poor)	3/60 (5.0)	REF		REF	
Second quintile	6/60 (10)	**2.2 (1.2-4.1)**	**0.015**	**4.1 (1.5-12)**	**0.007**
Third quintile	3/58 (5.0)	1.2 (0.38-4.0)	0.74	1.3 (0.17-10)	0.79
Fourth quintile	5/59 (8.5)	1.9 (0.90-4.2)	0.091	1.0 (0.35-3.0)	0.96
Fifth quintile (poorest)	15/59 (25)	**6.2 (2.8-14)**	**<0.001**	**6.7 (2.1-21)**	**0.001**
NHIF member					
Yes	9/84 (11)	REF		REF	
No	23/212 (11)	**1.0 (1.01-1.04)**	**0.001**	1.4 (0.76-2.6)	0.28
Number of coping strategies used					
None or single coping strategy used	22/259 (8.5)	REF		—	
Multiple coping strategies used	10/37 (27)	**3.7 (2.7-5.0)**	**<0.001**		
Diagnosis					
Any diagnosis	22/229 (9.5)	REF		—	
No diagnosis	10/67 (15)	1.6 (0.40-6.9)	0.49		
Healthcare facility and level at which seeking care					
Mitungu Health Centre (Level 3)	1/85 (1.0)	REF		REF	
Laare Health Centre (Level 3)	3/79 (4.0)	**3.5 (2.9-4.2)**	**<0.001**	**3.4 (2.3-5.0)**	**<0.001**
Mutuati Subcounty Hospital (Level 4)	3/38 (8.0)	**7.8 (6.0-10)**	**<0.001**	**7.1 (4.7-11)**	**<0.001**

Kanyakine Subcounty Hospital (Level 4)	6/31 (19)	**30 (17-50)**	**<0.001**	**31 (16-60)**	**<0.001**
Meru Teaching and Referral Hospital (Level 5)	19/63 (30)	**57 (28-118)**	**<0.001**	**75 (36-155)**	**<0.001**

Legend: In this sensitivity analysis, the catastrophic health expenditure threshold is calculated as total direct medical costs of participant of >10% of total monthly household expenditure. In contrast to the main analysis in Table 4 of the manuscript, the direct non-medical costs of the participant and the person or carer accompanying them such as food, travel, and accommodation, are not included in the calculation. Interaction testing of factors associated with catastrophic health expenditure found a significant interaction between coping strategies and poverty level and coping strategies was dropped from the multivariable model. The interaction testing for this model is not shown in this supplementary file.

Supplementary Table 7: Total costs sensitivity analysis showing univariable and multivariable logistic regression of health and social factors associated with catastrophic health expenditure by healthcare level

In this sensitivity analysis, the 10% catastrophic health expenditure threshold is calculated as total direct medical costs of participant, lost income of the participant, and total direct non-medical costs of the participant and any person/carer accompanying them (including food, travel, and accommodation) of >10% of total monthly household expenditure.

	**Catastrophic health expenditure at 10% of household monthly expenditure using total costs (medical costs, non-medical costs and lost income)**				
	**n/N (%)**	**Unadjusted univariable odds ratio (95%CI)**	**p value**	**Adjusted multivariable odds ratio (95%CI)**	**p value**
Catastrophic health expenditure	94/296 (32)				
Age groups					
18-29 years	23/103 (22)	REF		REF	
30-44 years	34/117 (38)	**2.8 (1.7-4.7)**	**<0.001**	1.7 (0.53-5.3)	0.38
45-59 years	22/60 (37)	**2.5 (1.5-4.3)**	**0.001**	1.4 (0.43-4.6)	0.58
60 years and above	4/16 (25)	1.3 (0.50-3.4)	0.60	0.76 (0.21-2.8)	0.67
Gender					
Male	46/123 (37)	REF		REF	
Female	48/173 (28)	**0.64 (0.42-0.98)**	**0.039**	**1.4 (1.1-1.8)**	**0.011**
Education level					
Up to primary school completion	47/163 (29)	REF		REF	
Up to secondary school completion	30/94 (32)	**1.5 (1.1-2.0)**	**0.003**	1.1 (0.95-1.4)	0.15
Above secondary school	17/39 (44)	**2.8 (1.1-6.8)**	**0.027**	1.3 (0.43-4.2)	0.61

Smoker					
Never smoker	76/253 (30)	REF		_	
Current or past smoker	18/43 (42)	1.3 (0.75-2.3)	0.33		
Primary income earner					
No	64/248 (26)	REF		REF	
Yes	30/48 (63)	**4.3 (3.0-6.2)**	**<0.001**	**6.1 (3.8-9.7)**	**<0.001**
Accompanied during healthcare seeking and visits					
No	58/237 (24)	REF		REF	
Yes	36/59 (61)	**5.3 (4.0-7.1)**	**<0.001**	**2.7 (1.9-3.9)**	**<0.001**
Asset-based poverty score					
First quintile (least poor)	17/60 (28)	REF		REF	
Second quintile	17/60 (28)	1.2 (0.54-2.5)	0.69	**1.9 (1.3-2.8)**	**0.002**
Third quintile	12/58 (21)	0.79 (0.48-1.3)	0.37	0.78 (0.55-1.1)	0.19
Fourth quintile	20/59 (34)	1.3 (0.88-1.9)	0.20	0.49 (0.14-1.7)	0.27
Fifth quintile (poorest)	28/59 (47)	**2.2 (0.1-4.3)**	**0.027**	2.0 (0.56-7.5)	0.28
NHIF member					
Yes	29/84 (35)	REF		_	_
No	65/212 (31)	1.1 (0.62-1.9)	0.79		
Number of coping strategies used (%)					
None or single coping strategy used	72/259 (28)	REF		_	_
Multiple coping strategies used	22/37 (59)	**3.7 (1.4-9.7)**	**0.009**		
Diagnosis					
Any diagnosis	68/229 (30)	REF		_	_
No diagnosis	26/67 (39)	1.5 (0.53-4.1)	0.45		
Health system level at which seeking care					
Health centre	16/164 (10)	REF		REF	

Sub-county hospital	34/69 (49)	**9.1 (8.4-9.7)**	**<0.001**	**9.9 (8.5-12)**	**<0.001**
County hospital	44/63 (70)	**20 (19-21)**	**<0.001**	**22 (14-35)**	**<0.001**

Legend: In this sensitivity analysis, the catastrophic health expenditure threshold is calculated as total direct medical costs of participant, lost income of the participant, and total direct non-medical costs of the participant and any person/carer accompanying them (including food, travel, and accommodation) of >10% of total monthly household expenditure. Interaction testing of the factors associated with 10% catastrophic health expenditure found a significant interaction between coping strategies and education level and coping strategies was dropped from the multivariable model. The interaction testing for this model is not shown in this supplementary file.

Supplementary Table 8: Total costs sensitivity analysis showing univariable and multivariable logistic regression of health and social factors associated with catastrophic health expenditure by five healthcare facilities

	Catastrophic health expenditure at 10% of monthly total household expenditure threshold using total costs (medical costs, non-medical costs and lost income)				
	**n/N (%)**	**Unadjusted univariable odds ratio (95%CI)**	**p value**	**Adjusted multivariable odds ratio (95%CI)**	**p value**
Catastrophic health expenditure	94/296 (32)				
Age groups					
18-29 years	23/103 (22)	REF		REF	
30-44 years	34/117 (38)	**2.8 (1.7-4.7)**	**<0.001**	1.7 (0.73-3.8)	0.23
45-59 years	22/60 (37)	**2.5 (1.5-4.3)**	**0.001**	1.2 (0.51-3.0)	0.65
60 years and above	4/16 (25)	1.3 (0.50-3.4)	0.60	0.74 (0.16-3.4)	0.45
Gender					
Male	46/123 (37)	REF		REF	
Female	48/173 (28)	**0.64 (0.42-0.98)**	**0.039**	**1.5 (1.0-2.1)**	**0.041**
Education level					
Up to primary school completion	47/163 (29)	REF		REF	
Up to secondary school completion	30/94 (32)	**1.5 (1.1-2.0)**	**0.003**	1.0 (0.51-2.0)	0.96
Above secondary school	17/39 (44)	**2.8 (1.1-6.8)**	**0.027**	1.3 (0.53-2.9)	0.61
Smoker					
Never smoker	76/253 (30)	REF		_	_
Current or past smoker	18/43 (42)	1.3 (0.75-2.3)	0.33		

Primary income earner					
No	64/248 (26)	REF		REF	
Yes	30/48 (63)	**4.3 (3.0-6.2)**	**<0.001**	**5.5 (2.4-13)**	**<0.001**
Accompanied during healthcare seeking and visits					
No	58/237 (24)	REF		REF	
Yes	36/59 (61)	**5.3 (4.0-7.1)**	**<0.001**	**2.4 (1.5-4.0)**	**0.001**
Asset-based poverty score					
First quintile (least poor)	17/60 (28)	REF		REF	
Second quintile	17/60 (28)	1.2 (0.54-2.5)	0.69	**2.1 (1.5-2.9)**	**<0.001**
Third quintile	12/58 (21)	0.79 (0.48-1.3)	0.37	0.79 (0.51-1.2)	0.27
Fourth quintile	20/59 (34)	1.3 (0.88-1.9)	0.20	0.50 (0.22-1.1)	0.099
Fifth quintile (poorest)	28/59 (47)	**2.2 (0.1-4.3)**	**0.027**	2.0 (0.53-7.8)	0.30
NHIF member					
Yes	29/84 (35)	REF		_	_
No	65/212 (31)	1.1 (0.62-1.9)	0.79		
Number of coping strategies used (%)					
None or single coping strategy used	72/259 (28)	REF		_	_
Multiple coping strategies used	22/37 (59)	**3.7 (1.4-9.7)**	**0.009**		
Diagnosis					
Any diagnosis	68/229 (30)	REF		_	_
No diagnosis	26/67 (39)	1.5 (0.53-4.1)	0.45		
Healthcare facility and level at which seeking care					
Mitungu Health Centre (Level 3)	5/85 (6)	REF		REF	
Laare Health Centre (Level 3)	8/79 (10)	**2.7 (2.5-2.9)**	**<0.001**	**2.0 (1.6-2.4)**	**<0.001**
Mutuati Subcounty Hospital (Level 4)	5/38 (13)	**9.8 (9-10.5)**	**<0.001**	**9.7 (8.4-11)**	**<0.001**

Kanyakine Subcounty Hospital (Level 4)	11/31 (35)	**30 (25-36)**	**<0.001**	**26 (22-32)**	**<0.001**
Meru Teaching and Referral Hospital (Level 5)	21/63 (33)	**37 (31-44)**	**<0.001**	**36 (30-43)**	**<0.001**

Legend: In this sensitivity analysis, the catastrophic health expenditure threshold is calculated as total direct medical costs of participant, lost income of the participant, and total direct non-medical costs of the participant and any person/carer accompanying them (including food, travel, and accommodation) of >10% of total monthly household expenditure. Interaction testing of the factors associated with catastrophic health expenditure found a significant interaction between coping strategies and education level and coping strategies was dropped from the multivariable model. The interaction testing for this model is not shown in this supplementary file.

Supplementary File 10: post-hoc exploratory, descriptive analysis

Post-hoc supplementary, descriptive analysis were performed and compared catastrophic health expenditure at 10% threshold, medical costs, non-medical costs, and lost income across three healthcare levels and across five healthcare facilities using Kruskal Wallis or ANOVA where appropriate.

Supplementary Figure 2a: Catastrophic health expenditure at 10% of total monthly household expenditure threshold by facility level

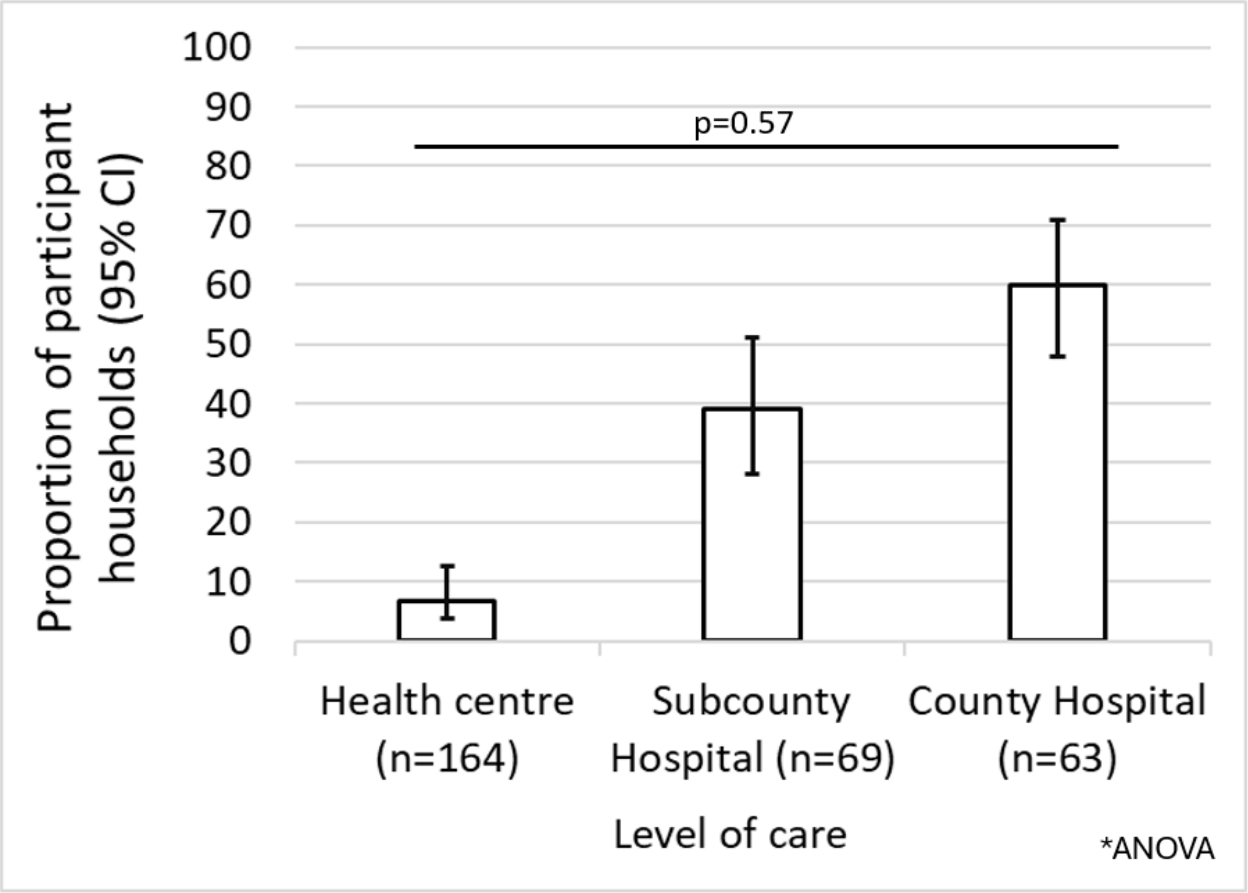

Supplementary Figure 2b: Mean direct medical costs, direct non-medical costs, and lost income of participants by facility level

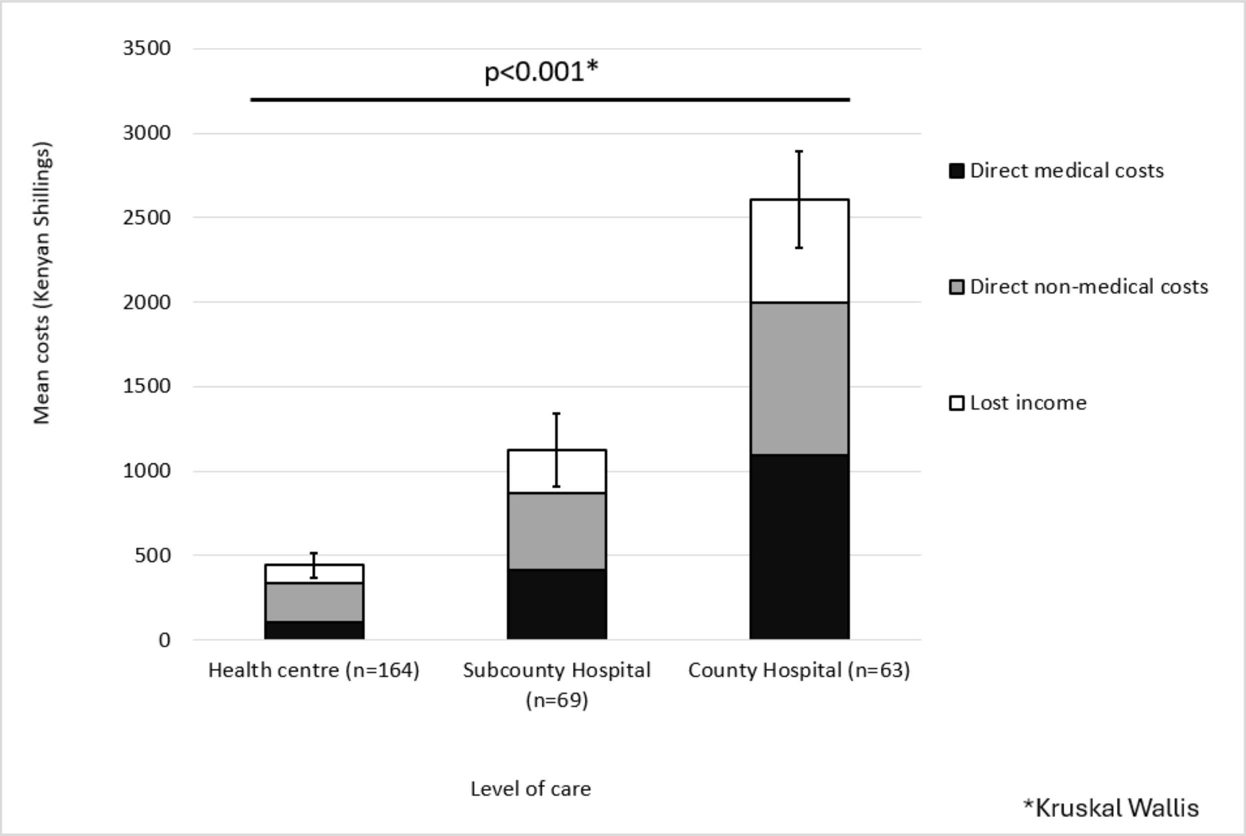

Supplementary Figure 2c: Direct medical costs, direct non-medical costs, and lost income as a proportion of total costs of participants by facility level

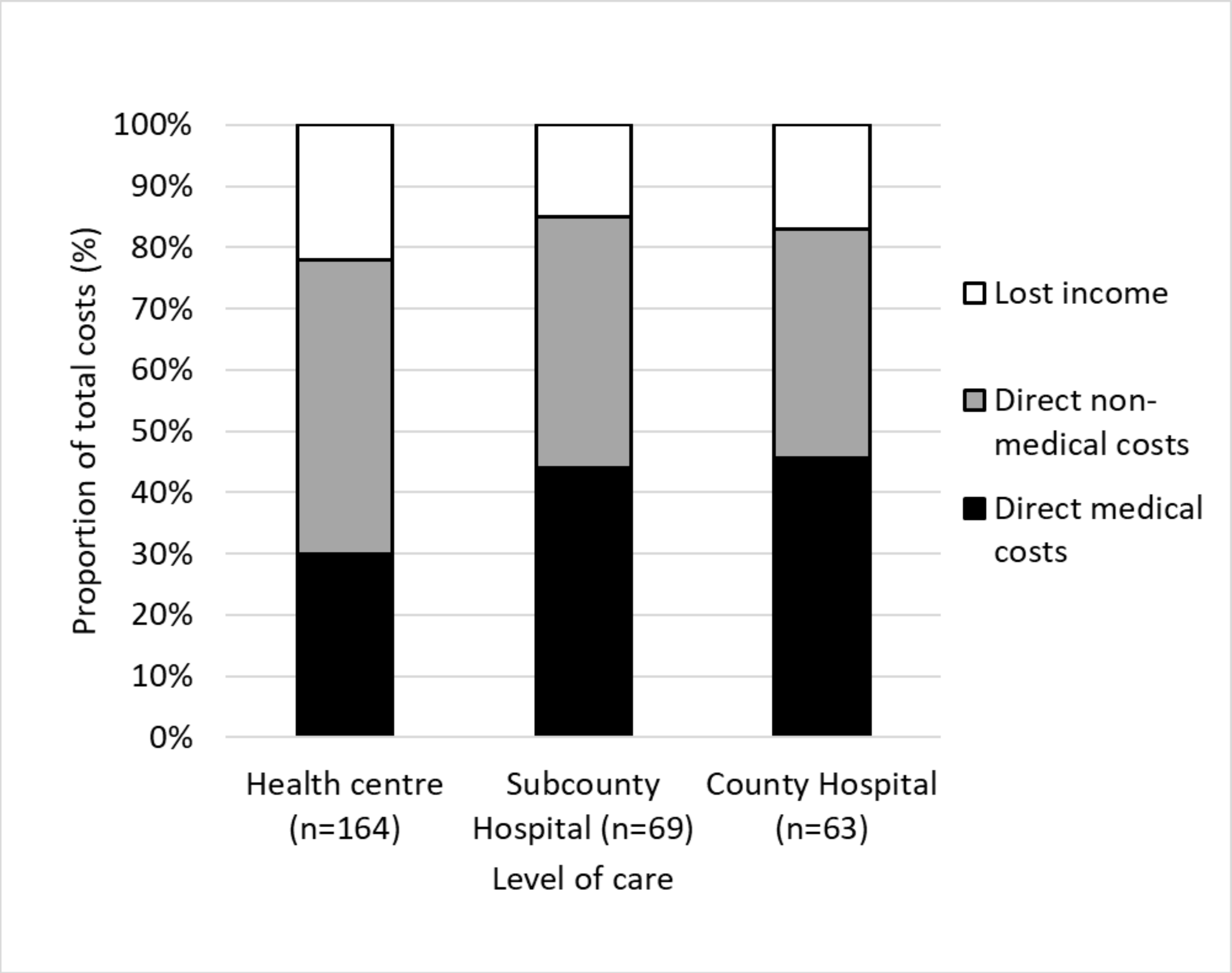

Supplementary Figure 3a: Catastrophic health expenditure at 10% of total monthly household expenditure threshold by healthcare facility

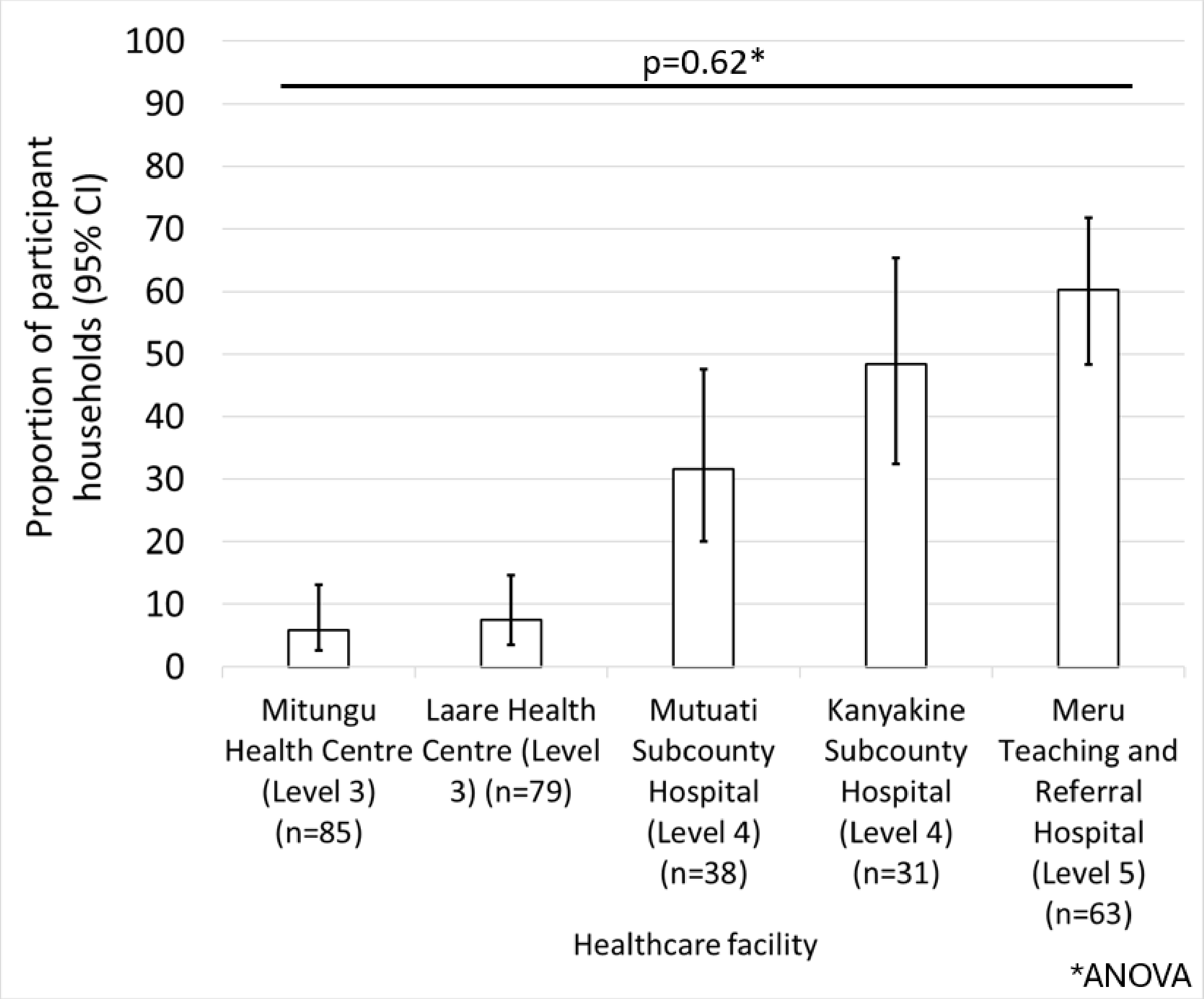

Supplementary Figure 3b: Mean direct medical costs, direct non-medical costs, and lost income of participants by healthcare facility

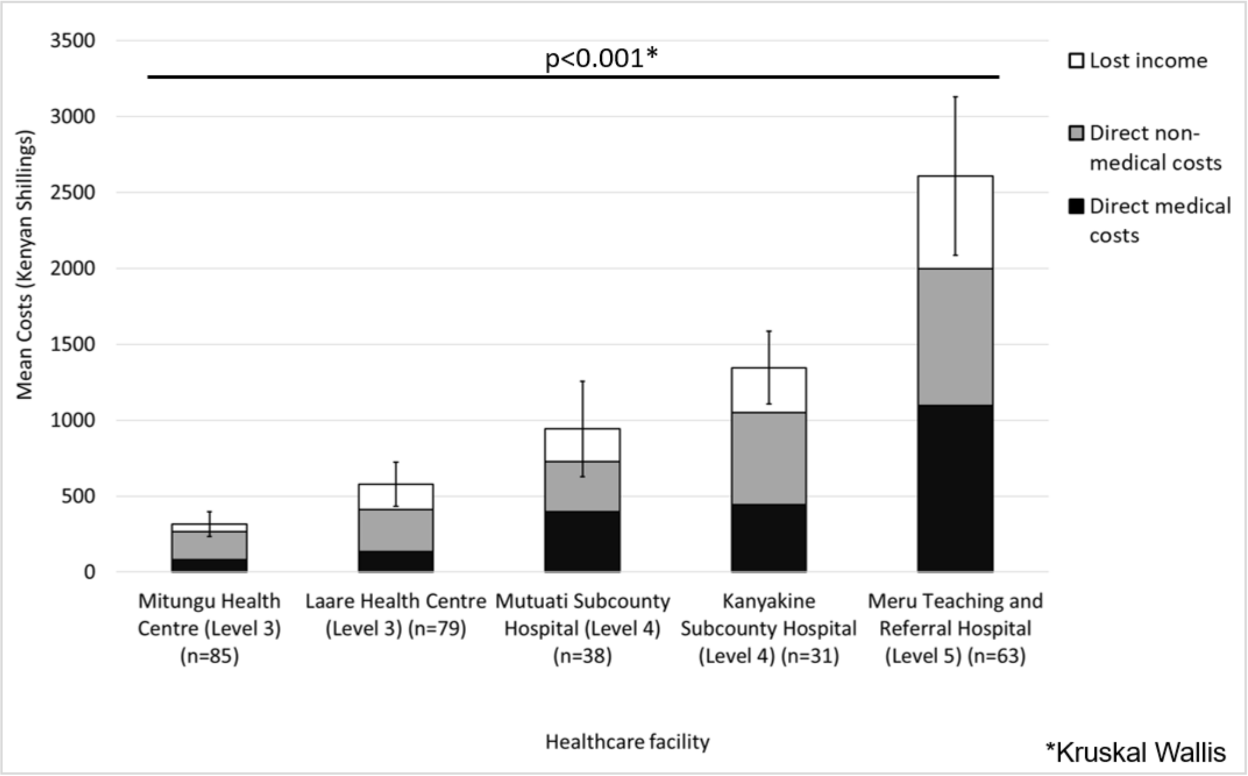

Supplementary Figure 3c: Direct medical costs, direct non-medical costs, and lost income as a proportion of total costs of participants by healthcare facility

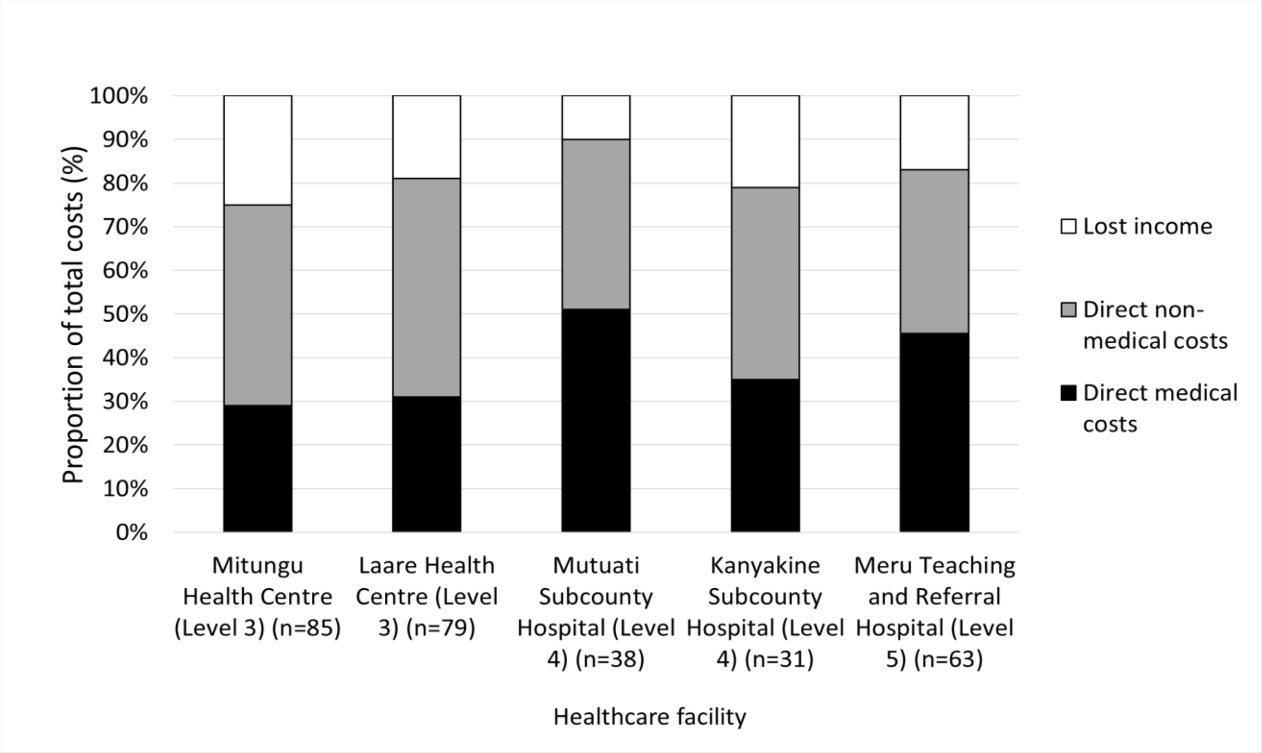

References

1. Kenya National Bureau of Statistics. Kenya Population and Housing Census Volume I: Population by County and Sub-County. In: Kenya National Bureau of Statistics, editor. Nairobi2019.
2. Mauch V, Woods N, Kirubi B, Kipruto H, Sitienei J, Klinkenberg E. Assessing access barriers to tuberculosis care with the tool to Estimate Patients' Costs: pilot results from two districts in Kenya. BMC public health. 2011;11(1):1-9.
3. Ministry of Health. Meru County: Health at a glance. In: Ministry of Health, editor. 2015.
4. World Health Organization. Tuberculosis patient cost surveys: a handbook. Geneva: World Health Organization; 2017.
5. Ministry of Health. The First Kenya Tuberculosis Patient Cost Survey. Nairobi:: National Tuberculosis, Leprosy, and Lung Diseases Program; 2017.
6. Jolliffe IT, Cadima J. Principal component analysis: a review and recent developments. Philosophical transactions of the royal society A: Mathematical, Physical and Engineering Sciences. 2016;374(2065):20150202.
7. Cerny BA, Kaiser HF. A study of a measure of sampling adequacy for factor-analytic correlation matrices. Multivariate behavioral research. 1977;12(1):43-7.
8. Wingfield T, Tovar MA, Huff D, Boccia D, Montoya R, Ramos E, et al. The economic effects of supporting tuberculosis-affected households in Peru. European respiratory journal. 2016;48(5):1396-410.
9. Rocha C, Montoya R, Zevallos K, Curatola A, Ynga W, Franco J, et al. The innovative socio-economic interventions against tuberculosis (ISIAT) project: an operational assessment. The International journal of tuberculosis and lung disease. 2011;15(6):S50-S7.
