# Supplementary material for: Catastrophic health expenditure, social protection coverage, and financial coping strategies in adults with symptoms of chronic respiratory diseases in Kenya: a cross-sectional study
Source: Lancet Glob Health. 2025 Jun 25;13(7):e1301–13. doi: 10.1016/S2214-109X(25)00061-0 (PMC12208783; doi:10.1016/S2214-109X(25)00061-0)
Supplement: Equitable Partnership Declaration [file mmc2.pdf]

THE LANCET
Global Health

Supplementary appendix 2

This Equitable Partnership Declaration (EPD) was submitted by the authors, and we reproduce it as supplied. It has not been peer reviewed. *The Lancet*'s editorial processes have not been applied to the EPD.

Supplement to: Mulupi S, Waithera C, Tomeny EM, et al. Catastrophic health expenditure, social protection coverage, and financial coping strategies in adults with symptoms of chronic respiratory diseases in Kenya: a cross-sectional study. *Lancet Glob Health* 2025; **13:** e1301–13.

Equitable Partnership Declaration

Researcher considerations

1. Please detail the involvement that researchers who are based in the region(s) of study had during a) study design; b) clinical study processes, such as processing blood samples, prescribing medication, or patient recruitment; c) data interpretation; and d) manuscript preparation, commenting on all aspects. If they were not involved in any of these aspects, please explain why.

   This question is intended for international partnerships; if all your authors are based in the area of study, this question is not applicable.

   This should include a thorough description of their leadership role(s) in the study. Are local researchers named in the author list or the acknowledgements, or are they not mentioned at all (and, if not, why)? Please also describe the involvement of early career researchers based in the location of the study. Some of this information might be repeated from the Contributors section in the manuscript. Note: we adhere to [ICMJE authorship criteria] when deciding who should be named on a paper.

**a) Study design:**<br><br>Researchers based in Kenya, (the study site) led in study design
**b) Clinical study processes:**<br><br>Researchers based in Kenya led the entire process of data collection and stakeholder engagement
**c) Data interpretation:**<br><br>Researchers based in Kenya were centrally involved in all the data analysis processes
**d) Manuscript preparation:**<br><br>SM-an early career researcher wrote the first draft, all authors reviewed and contributed

2. Were the data used in your study collected by authors named on the paper, or have they been extracted from a source such as a national survey? ie, is this a secondary analysis of data that were not collected by the authors of this paper. If the authors of this paper were not involved in data collection, how were data interpreted with sufficient contextual knowledge?

   The Lancet Global Health *believe contextual understanding is crucial for informed data analysis and interpretation.*

Data used in this study were collected by authors named. Majority (6 out of 10) live in Kenya and have sufficient contextual knowledge of the Kenyan health system and study sites.

3. How was funding used to remunerate and enhance the skills of researchers and institutions based in the area(s) of study? And how was funding used to improve research infrastructure in the area of study?

   Potentially effective investments into long-term skills and opportunities within institutions could include training or mentorship in analytical techniques and manuscript writing, opportunities to lead all or specific aspects of the study, financial remuneration rather than requiring volunteers, and other professional development and educational opportunities.

   Improvements to research infrastructure could be funding of extended trial designs (such as platform trials) and use of master protocols to enable these designs, establishment of long-term contracts for research staff, building research facilities, and local control of funding allocation.

**Skills:** The funding in this study was used primarily to train SM for a doctoral degree. Other research processes e.g. training in data collection, analysis, research ethics, stakeholder engagement, have enhanced skills of the research team.
**Research infrastructure:** Funding was used to enhance local institution infrastructure in KEMRI-CRDR e.g. through purchase of printers, internet communication facilities, data collection equipment- eg digital voice recorders, computers, purchase of software.

4. How did you safeguard the researchers who implemented the study?

   Please describe how you guaranteed safe working conditions for study staff, including provision of appropriate personal protective equipment, protection from violence, and prevention of overworking.

This research was conducted in compliance of highest international ethical standards All staff were trained and regularly reminded on applications of ethical principals; The research team worked from 8AM-5 PM, were vaccinated against Hep B and rabies- common risks in our environment; and provided protective wear. The recruitment process was gender-sensitive. Research team members were provided leave days, and did not work during weekends.

<u>*Benefits to the communities and regions of study*</u>

5. How does the study address the research and policy priorities of its location?

   How were the local priorities determined and then used to inform the research question? Who decided which priorities to take forward? Which elements of the study address those priorities?

Universal health coverage is a key agenda for the government of the day- this study focused on respiratory diseases and health financing- both top priorities for Kenya. The study site- Meru, was selected based on burden of TB. The study question was decided following gaps in literature on health financing and risks of out of pocket expenditures for diseases like asthma.

6. How will research products be shared in the community of study?

For instance, will you be providing written or oral layperson summaries for non-academic information sharing? Will study data be made available to institutions in the region(s) of study? The Lancet Global Health *encourages authors to translate the summary (abstract) into relevant languages after paper editing; do you intend to translate your summary?*

Study findings will be translated to simple english and disseminated in local language- Swahili to reach both clinical and lay audiences. We are actively engaging diverse policy communities in the research findings through policy forums, conferences and meetings.

7. How were individuals, communities, and environments protected from harm?

a) *How did you ensure that sensitive patient data was handled safely and respectfully? Was there any potential for stigma or discrimination against participants arising from any of the procedures or outcomes of the study?*

All data were handled confidentially- our publication does not contain any identifiable data- electronic data were stored in password protected computers accessible only by the study team

b) *Might any of the tests be experienced as invasive or culturally insensitive?*

N/A

c) *How did you determine that work was sensitive to traditions, restrictions, and considerations of all cultural and religious groups in the study population?*

This study is one of the substudies in a larger study exploring readiness of Kenyan health system to respond to asthma. Other substudies using qualitative methods showed that asthma is a stigmatised condition. Our engagements were respectful and confidential.

d) *Were biowaste and radioactive waste disposed of in accordance with local laws?*

N/A

e) *Were any structures built that would have impacted members of the community or the environment (such as handwashing facilities in a public space)? If so, how did you ensure that you had appropriate community buy-in?*

No

f) *How might the study have impacted existing health-care resources (such as staff workloads, use of equipment that is typically employed elsewhere, or reallocation of public funds)?*

The engagements in this study prioritised well being of people who had come to health facilities for services- research team collected all data in this study. Healthcare workers were consulted at times that they were not serving patients. We did not use any equipment/ reallocate public funds.

8. Finally, please provide the title (eg, Dr/Prof, Mr/Mrs/Ms/Mx), name, and email address of an author who can be contacted about this statement. This can be the corresponding author.

Name: Dr Tom Wingfield.
Email: Tom.Wingfield@lstmed.ac.uk
